# Tissue-Specific Gene Repositioning by Muscle Nuclear Membrane Proteins Enhances Repression of Critical Developmental Genes during Myogenesis

**DOI:** 10.1016/j.molcel.2016.04.035

**Published:** 2016-06-16

**Authors:** Michael I. Robson, Jose I. de las Heras, Rafal Czapiewski, Phú Lê Thành, Daniel G. Booth, David A. Kelly, Shaun Webb, Alastair R.W. Kerr, Eric C. Schirmer

**Affiliations:** 1 The Wellcome Trust Centre for Cell Biology and Institute of Cell Biology, University of Edinburgh, Edinburgh EH9 3BF, UK

## Abstract

Whether gene repositioning to the nuclear periphery during differentiation adds another layer of regulation to gene expression remains controversial. Here, we resolve this by manipulating gene positions through targeting the nuclear envelope transmembrane proteins (NETs) that direct their normal repositioning during myogenesis. Combining transcriptomics with high-resolution DamID mapping of nuclear envelope-genome contacts, we show that three muscle-specific NETs, NET39, Tmem38A, and WFS1, direct specific myogenic genes to the nuclear periphery to facilitate their repression. Retargeting a NET39 fragment to nucleoli correspondingly repositioned a target gene, indicating a direct tethering mechanism. Being able to manipulate gene position independently of other changes in differentiation revealed that repositioning contributes ⅓ to ⅔ of a gene’s normal repression in myogenesis. Together, these NETs affect 37% of all genes changing expression during myogenesis, and their combined knockdown almost completely blocks myotube formation. This unequivocally demonstrates that NET-directed gene repositioning is critical for developmental gene regulation.

## Highlights

• Tissue-specific NETs direct repositioning of critical muscle genes during myogenesis
• Expression changes for NET-repositioned genes depend on cell differentiation state
• Isolating position from differentiation reveals its contribution to gene expression
• Three NETs together affect 37% of all genes normally changing in myogenesis

## Graphical Abstract

**Figure:**
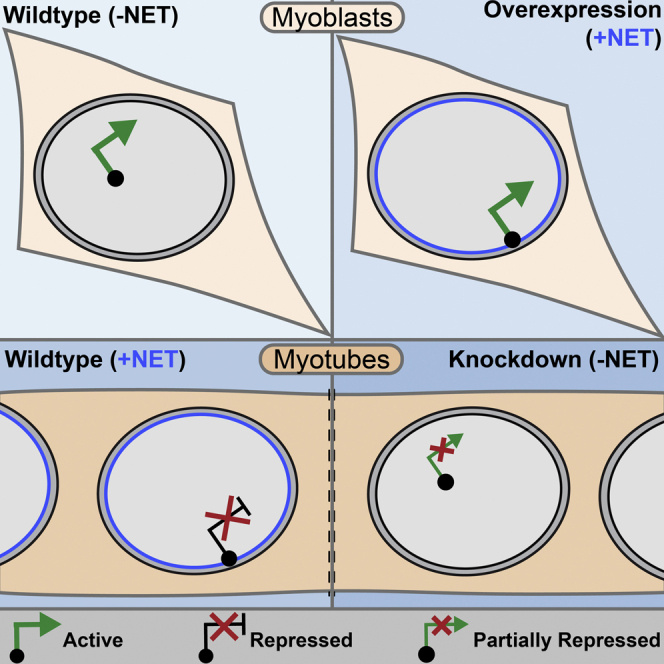

## Teaser

Muscle-specific nuclear envelope transmembrane proteins (NETs) optimize myogenic gene expression by physically recruiting genes to the periphery and enhancing their repression. Specifically manipulating the position of endogenous genes in myoblasts and myotubes indicates that peripheral localization enhances repression, but only in context of other changes in differentiation.

## Introduction

Repositioning certain developmentally important genes between the nuclear periphery and interior during differentiation correlates with changes in their expression state. For example, concomitant with their activation, the *MyoD, IgH*, and *Mash1* genes reposition from the periphery to the nuclear interior respectively during myogenic, B cell, and neuronal differentiation. Conversely, the *c-maf* locus repositions to the periphery as it is repressed during T cell differentiation (Wong et al., 2014, Zuleger et al., 2011). New genome-wide approaches have identified many developmentally important genes that change in their peripheral association during differentiation with correspondingly altered expression (Peric-Hupkes et al., 2010). These findings raise the questions: how are gene positioning patterns established and regulated during development, and, to what extent does altered nuclear position contribute to changes in a genes expression?

Although little is known about the regulation of specific developmental gene repositioning to the periphery, nuclear envelope (NE) proteins clearly direct establishment of genome-wide patterns of peripheral heterochromatin organization. Peripheral heterochromatin is disrupted in mice lacking the NE transmembrane protein (NET) LBR and lamin A (Solovei et al., 2013). Lamins also contribute to more specific gene repositioning in *C. elegans* muscle, but the mechanism remains elusive (Mattout et al., 2011). Similarly, in mouse fibroblasts, lamin B1 and the NET LAP2β function with transcriptional regulators to peripherally target the *IgH* and *Cyp3a* loci, but it is unclear how the *IgH* locus is specifically released in pro-B cells (Zullo et al., 2012). However, the near ubiquitous expression of lamins, LAP2β and LBR and the general nature of these interactions argue for other mechanisms to explain tissue-specific aspects of gene positioning.

Determining peripheral positioning’s contribution to regulation of gene expression is stymied by the fact that gene repositioning in development is accompanied by changes in transcriptional regulators, epigenetic marks, and other genome changes associated with differentiation. Circumventing this by directing reporter genes to the NE in the absence of differentiation used artificial locus-NE protein interactions (Finlan et al., 2008, Kumaran and Spector, 2008, Reddy et al., 2008); however, these studies yielded inconsistent results concerning the effect of repositioning on the expression of tethered reporters. Random genome-wide insertions of reporter genes yielded significant repression when integrated into peripherally localized genome regions (Akhtar et al., 2013). More recently, TALEN-driven chromatin unfolding sufficed to release endogenous developmental genes from the periphery, suggesting position may in some cases reflect a gene’s state of folding (Therizols et al., 2014). However, it is unclear if chromatin unfolding can drive repositioning for a range of developmental genes or if other more specific directed mechanisms normally function in development. Addressing these questions requires identification of the endogenous proteins responsible for peripheral gene localization and their manipulation in both differentiated and undifferentiated cells.

We recently identified several tissue-specific NETs that direct several chromosomes to the nuclear periphery in fibroblasts (Zuleger et al., 2013). We hypothesized that chromosome repositioning reflects a summation of individual specific gene repositioning events directed by tissue-specific NETs during differentiation and that repositioning contributes to gene regulation. Combining transcriptome analysis in myogenesis with NET knockdowns (KDs) and global gene positioning changes determined by DamID (Vogel et al., 2007), we for the first time show the NET dependence of specific gene repositioning and its consequences for gene repression in myogenesis. Critically, the ability to specifically target the NET independently of other changes in myogenesis demonstrates that gene repositioning adds another layer to gene regulation during development.

## Results

### NET39 Directs Chromosome 8 Repositioning In Myogenesis

**Figure 1:**
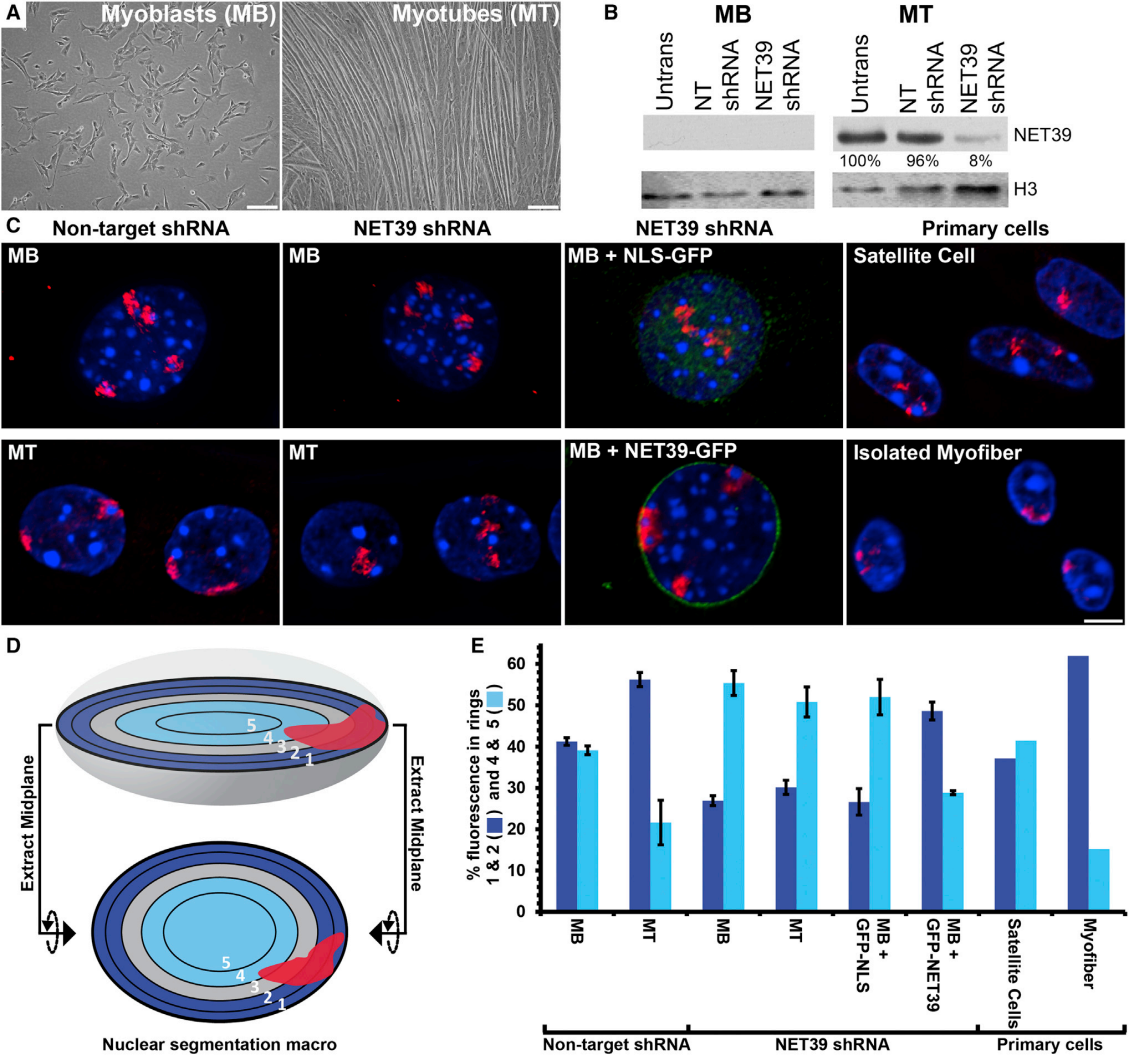
NET39 Determines the Position of Chromosome 8 during Myogenesis (A) Micrographs showing normal C2C12 in vitro myogenic differentiation with undifferentiated MBs forming multi-nucleated MTs. (B) Western blot confirming the depletion of NET39 in NET39-shRNA treated MTs. (C) Representative images of the position of chromosome 8 in indicated samples. The scale bar represents 5 μm. (D) Schematic describing analysis of chromosome position at the nuclear midplane. (E) Quantification of chromosome 8 position in indicated samples. The error bars represent the SD of the means of two biological repeats of at least 50 nuclei each. For primary cells, single experiments were performed and so the error bars are absent. See also Figure S1.

NET39 is induced early in myogenesis (Liu et al., 2009) and could reposition a subset of chromosomes to the nuclear periphery in fibroblasts (Zuleger et al., 2013). This suggested that NET39—and potentially other muscle NETs—might direct gene and chromosome positioning changes during myogenesis. Accordingly, we investigated its contribution to chromosome positioning using the C2C12 system, in which proliferating mouse myoblasts (MBs) can be induced to differentiate into myotubes (MTs) (Yaffe and Saxel, 1977) (Figure 1A). Using whole chromosome fluorescence in situ hybridization (FISH), we first tested whether exogenous NET39 expression could increase peripheral localization of four chromosomes (8, 10, 11, and 16), finding chromosome 8 strongly affected (Figure S1A). Stable NET39 KD C2C12 lines were generated and transient NET39 overexpression was used to next test NET39 function during myogenesis. The target small hairpin (sh)RNA reduced NET39 protein levels by >90% in the MTs and to levels undetectable by western blot in the MBs (Figure 1B).

Chromosome 8 normally repositions from the interior in MBs to the nuclear periphery of MTs during myogenesis (Figure 1C, non-target shRNA). Repositioning was quantified using an algorithm that takes a midplane image and erodes the total nuclear area from the periphery by 20% in five consecutive steps to generate five concentric rings of equal area (Chubb et al., 2002) (Figure 1D). Although the large chromosome volume was distributed over several rings, this analysis confirmed a significant increase in the percentage of chromosome signal at the periphery with a mean of 56% in the most peripheral two rings (Figure 1E). NET39 KD not only abolished differentiation-associated repositioning, but also strongly reduced peripheral chromosome 8 in MBs indicating even basal NET39 levels undetectable by western blot (but detected by quantitative [q]RT-PCR, data not shown) can influence a chromosomes position (Figure 1E).

The repositioning activity lost by NET39 KD could be rescued by exogenously expressing human GFP-NET39, but not GFP-NLS in the NET39 KD MBs in the absence of differentiation (Figure 1E). All changes induced by altering NET39 levels were highly statistically significant using the Kolmogorov-Smirnov (KS) test (Figure S1B). Compellingly, similar chromosome 8 repositioning was observed in primary muscle cells with strong peripheral association in primary myofibers freshly isolated from mouse extensor digitorum longus (EDL) muscle paralleling C2C12 MTs, but not in primary satellite cells paralleling C2C12 MBs (Figures 1C and 1E, most right). These data clearly indicate that chromosome 8 repositions from the nuclear interior to the nuclear periphery during myogenesis and that this repositioning is largely driven by NET39.

### Global Determination of Gene Positioning Changes in Myogenesis

We next mapped global changes in genomic loci at the nuclear periphery during C2C12 myogenesis using DamID. A bacterial Dam methylase fused to lamin B1 preferentially methylated peripheral gene sequences which were isolated and identified by next generation sequencing (Vogel et al., 2007). To control for local variation in chromatin accessibility, soluble Dam methylase was expressed in parallel experiments. As MT fusion never reaches 100% in a population and both MTs and MBs were efficiently transduced, for MT preparations, MTs were isolated from remaining undifferentiated MBs by a short trypsin digestion step (Figures S2A and S2B). Genomic DNA was isolated from 3-day transduced cultures (taken at day 6 of MT differentiation), processed to enrich for Dam methylated DNA, and sequenced to yield 5.1- and 5.7-fold genome coverage in MBs and MTs, respectively.

**Figure 2:**
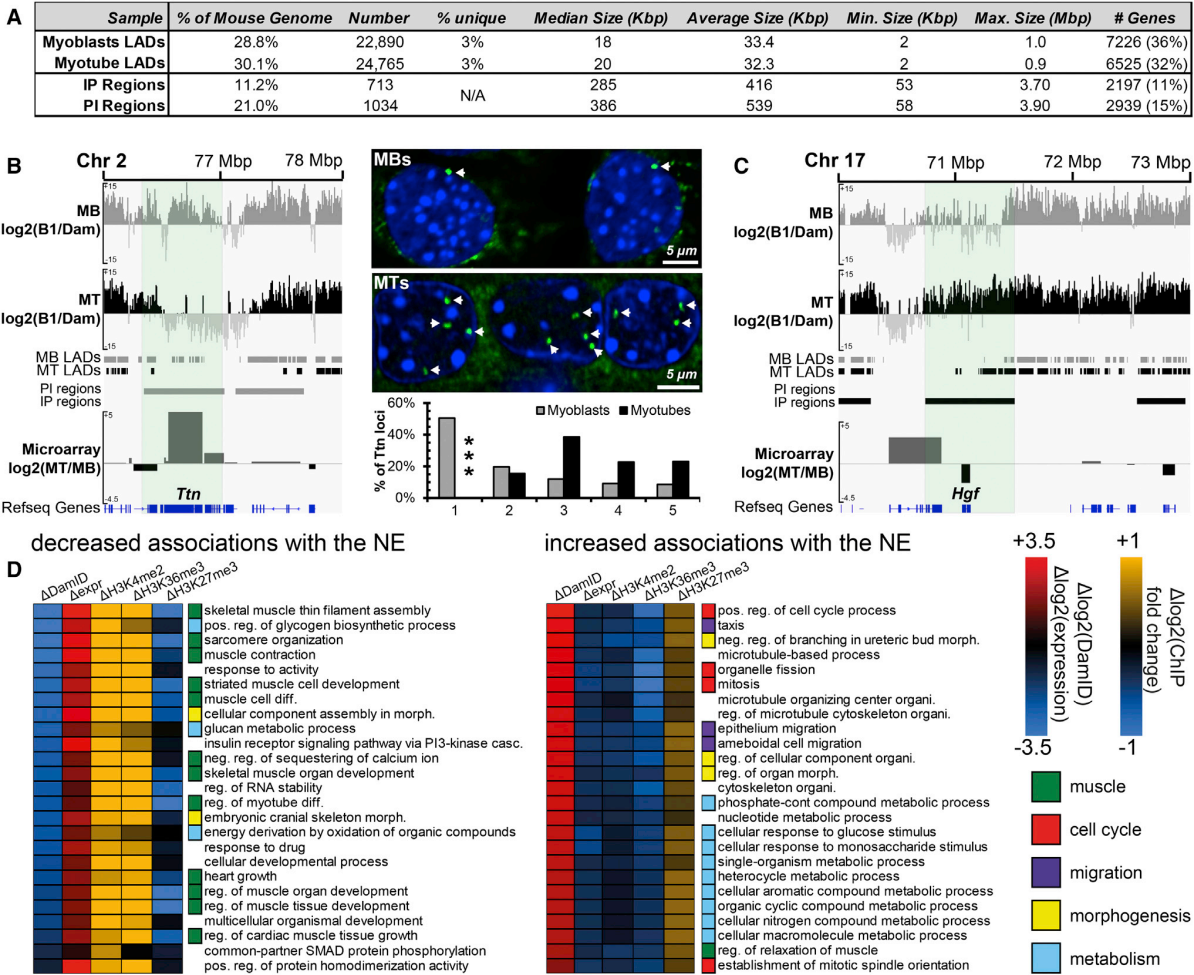
Global Identification of Loci Repositioning in C2C12 Myogenesis (A) Table summarizing parameters of identified LADs and regions showing increased (IP) and decreased (PI) association with the nuclear periphery. (B) Example genome browser view of the *Ttn* locus with representative images and direct quantification of the position of *Ttn* loci in 100 nuclei (green) by FISH in MBs and MTs. ^∗∗∗^p < 0.001 comparing locus position in MTs to MBs using χ^2^ test. (C) Genome browser view for the genomic region surrounding *Hgf* showing DamID signal intensities, identified LADs, IP and PI regions, and microarray gene expression changes for MBs and MTs. (D) Heatmap displaying average Δlog2 DamID, Δlog2 expression, and Δlog(fold change) for indicated histone modifications values between MTs and MBs for genes within GO term categories significantly enriched in PI activated genes and IP repressed genes. Myogenic alterations to histone modifications associated with transcriptionally active genes (H3K4me2 and H3K36me3) and the transcriptional repression-associated H3K27me3 were extracted from Asp et al. (2011). The Δlog2(DamID) value was calculated by subtracting the average log2(Lamin B1/Dam) value in a 100 kb window surrounding the gene in the MB sample from the MT sample. The ChIP-seq values were determined for each histone modification by subtracting the average signal across the gene body in the MB sample from the MT sample. See also Figures S2 and S3.

Log2(Lamin B1 Dam/soluble Dam) ratios were then generated and used to identify lamina associated domains (LADs) in MBs and MTs as described previously (Wu and Yao, 2013). Similar to an earlier DamID study differentiating embryonic stem cells into neuronal precursors (Peric-Hupkes et al., 2010), the majority of LADs identified in MBs were retained in MTs: 94% of the LAD coverage was shared between MTs and MBs with the remaining 6% roughly equally distributed between lost and newly formed LADs (Figure 2A). Although many LADs were shared between MBs and MTs, the signal intensity from log2(Lamin B1/Dam) ratios frequently differed considerably with discrete regions displaying a quantifiably increased or decreased ratio in MBs or MTs. This indicated a binary LAD or non-LAD definition is insufficient to identify regions with altered peripheral association between MBs and MTs. Since the log2(Lamin B1/Dam) signal represents the probability of peripheral localization (Peric-Hupkes et al., 2010), we instead developed a statistical method, described in detail in the Supplemental Information, to identify regions that exhibit significant changes in lamina association in MBs and MTs. In all, a tendency to relocate from the interior in MBs to the periphery in MTs (denoted “IP”) was observed for 713 regions that were often proximal to the edges of MB genome regions enriched in LADs, with an average size of 416 kb containing 2,197 genes. The opposite tendency (shifting from the periphery to the interior; “PI”) was observed for 1,034 regions with an average size of 539 kb containing 2,939 genes (Figures 2A and S3A). IP and PI regions can be observed in a chromosomal context in Figure S3A ideograms and all DamID identified genes are listed in Table S1.

Consistent with an earlier neuronal differentiation study (Peric-Hupkes et al., 2010), genes located within IP regions strongly tended to become repressed during differentiation instead of activated, though many were unchanged. Out of 2,197 genes in IP regions, 933 became at least 1.4-fold repressed, 991 were unchanged, and only 266 increased at least 1.4-fold in expression based on ENCODE C2C12 RNA-sequencing (seq) data (Yue et al., 2014). As our DamID time point was 2 days later in differentiation than available RNA-seq data, we performed expression analysis using microarrays to parallel the DamID. Consistent with similar published RNA-seq data (Figures S3B and S3C), microarray data revealed that genes in IP regions tend to be repressed, while genes in PI regions tend to be activated during differentiation. Titin, an important gene for muscle differentiation, was in this latter category (Figure 2B). Release of the Titin locus from the periphery during myogenesis was also evident from FISH, confirming both the DamID data and the statistical approach used to identify repositioning loci.

**Figure 3:**
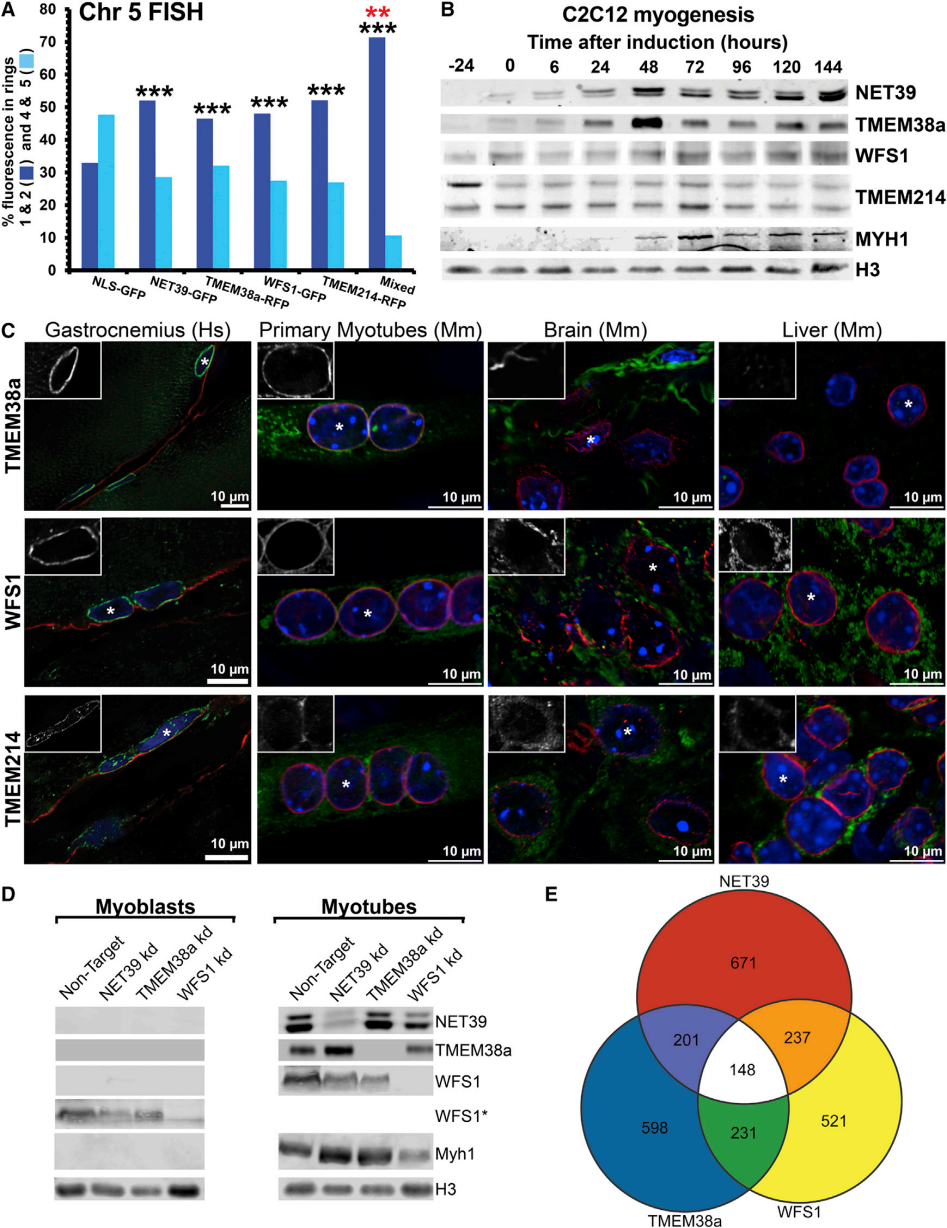
Muscle-Specific NETs Function Together to Reposition a Chromosome and Genes Affected by their KD (A) Quantification of the position of human chromosome 5 in at least 50 HT1080 fibroblast nuclei expressing a single or multiple (Mixed) NETs. ^∗∗∗^p < 0.001 comparing the position of chromosome 5 in the GFP-NET expressing cells to the NLS-GFP (black), and ^∗∗^p < 0.01 comparing mixed to individual NET expressing cells using KS tests (red). (B) Western blot time course of C2C12 differentiation for indicated antibodies. (C) Immunofluorescence staining of NETs (green), lamin A or B1 (red), and DNA (blue) in indicated tissue cryosections and fixed MTs generated in vitro from mouse EDL muscle-derived satellite cells. To identify myofibers in the gastrocnemius muscle section, dystrophin (red) was stained in lieu of lamins A and B1. The insert boxes show channels for NET staining individually for indicated nuclei (^∗^). (D) Western blot of pre- and post-differentiated control and NET-KD cell lines for indicated proteins. (E) Venn diagram of total gene expression changes exceeding log2 0.5 in absolute value between NET-KD MTs relative to empty-vector treated control MTs detected by microarray analysis performed in triplicate. See also Figure S4.

**Figure 4:**
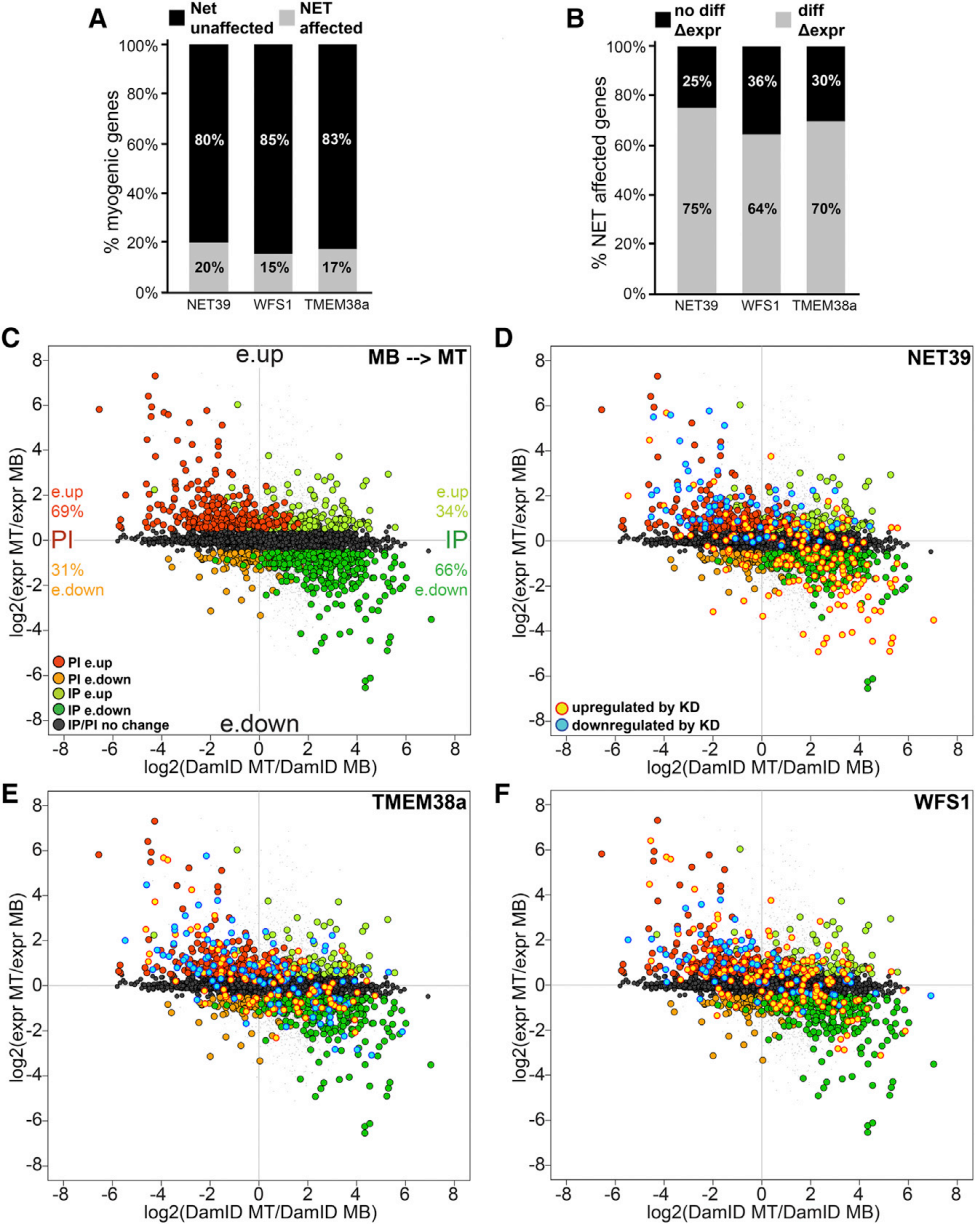
Global Correlations between Gene Positioning and Expression Directed by Muscle-Specific NETs (A) Fraction of total genes normally changing during myogenesis that are altered by NET depletion. (B) Fraction of total genes altered by NET depletion that normally change during myogenesis. (C) log2(expr MT/expr MB) myogenic gene expression changes inversely correlate with average log2(DamID MT/DamID MB) myogenic lamin B1 DamID signal intensities changes. Of IP genes with altered gene expression, 66% are repressed (e.down) and 34% are activated (e.up), while for PI genes, only 31% are repressed and 69% are activated. (D–F) Identical plots as (A) with genes highlighted if upregulated (yellow) or downregulated (blue) in indicated NET-depleted MTs relative to control MTs. The NET affected genes tend to anti-correlate, i.e., loss of the NET reduces a normal repression that occurs in myogenesis or reduces a normal increase in expression. All gene expression values are mean average changes of microarray triplicate samples. See also Figure 5.

Many PI regions contained important myogenic genes that need to be activated for muscle differentiation: *Ryr3* encodes a ryanodine receptor important for Ca^2+^ regulation, *Actc1* for muscle actin, and *Myo18b* for a muscle myosin (Figure S2C). Correspondingly, IP regions contained genes inhibitory to myogenesis that need to be repressed in MTs. For example, IP gene *Hgf* encodes hepatic growth factor that, although required for the activity of satellite cells, is inhibitory to myogenesis when present during differentiation (Figure 2C) (Dietrich et al., 1999, Yamada et al., 2010). Similarly, *Dbf4* and *Cdk14* products promote cell-cycle progression (Davidson et al., 2009, Kumagai et al., 1999) that, while required in cycling satellite cells, is detrimental in terminally differentiated MTs (Figure S2C). To globally associate repositioning with gene function, analysis of Gene Ontology (GO) term enrichment of repressed IP and activated PI genes was performed. The top 25 GO-terms for each are shown compared to a heatmap for their expression changes in the matched myogenic microarray samples and available chromatin immunoprecipitation (ChIP)-seq data for myogenesis (Asp et al., 2011) (Figure 2D). The expression changes for these PI region genes correlated with the loss of the repressive H3K27me3 mark and accumulation of active histone marks such as H3K4me2, H3K4me3, H3K9Ac, and H3K36me3 (Figure S2D). They also strongly associated with muscle functions and differentiation. In contrast, these IP genes correlated with loss of active histone marks and gain of H3K27me3, reflecting silencing of genes functioning in the cell cycle, cell migration, morphogenesis, and altered metabolism. There were no clear associations with GO-categories relating to myogenesis for IP and PI genes that were oppositely regulated in expression (Figures S3D–S3G). Hence, repositioning to and from the nuclear periphery during myogenesis is generally associated with repression of genes inhibitory to myogenesis and the activation of genes required for MTs, respectively. Processed DamID and microarray data used in Figures 2, 3, and 4 can be found in Table S1.

### Together, Multiple Muscle NETs Reposition Large Chromosomal Regions

Two liver-specific NETs functioned together to position chromosome 5 in human liver cells (Zuleger et al., 2013). Therefore, it seemed likely that additional muscle NETs might contribute to the global gene positioning changes between MBs and MTs revealed by DamID. Thus, NETs found in muscle NEs (Wilkie et al., 2011) were screened using the same strategy as described in Zuleger et al. (2013) (overexpression-induced repositioning of chromosome 5 in human fibroblasts). Tmem38a, WFS1, and Tmem214 were identified as additional candidates (Figure S4A). To test if these NETs can cooperate with NET39, WFS1 was fused to GFP and Tmem38a and Tmem214 were fused to RFP. The NETs were then expressed either alone or in combination. Only cells with both RFP and GFP were analyzed to ensure that at least two NETs were expressed in the combination sample. As expected, each muscle NET by itself significantly increased peripheral localization of chromosome 5 from ∼30% basal levels to ∼50% (Figure 3A). Strikingly, when all four muscle NETs were co-transfected, peripheral localization increased to over 70% and was significantly higher compared to any NET alone, indicating the repositioning activity of NETs is additive (Figure 3A).

These four proteins were next confirmed as muscle-specific NETs. By western blot, NET39 and Tmem38a proteins were absent from dividing MBs (−24 hr), but were weakly detectable in the confluent cultures required for induction of differentiation (0 hr). They were then strongly expressed by 48 hr after induction (Figure 3B), well before standard myogenic markers such as the myosin heavy chain (Myh1) are expressed. WFS1 and Tmem214 were already present in MBs, and WFS1 increased during differentiation while Tmem214 did not (Figure 3B). Hence, different subsets of muscle NETs capable of repositioning chromosomes are present as myogenesis progresses and so could contribute differently to genome organization. Tissue transcriptome/proteome (Uhlén et al., 2015) analysis of NET expression revealed NET39 and Tmem38a almost exclusively in heart and muscle, while WFS1 and Tmem214 were more widely expressed (Figure S4B). WFS1 and Tmem214 were previously reported to be in the ER (Kohara et al., 2014, Li et al., 2013, Takeda et al., 2001); so their endogenous distribution was investigated with antibodies in human gastrocnemius (calf muscle), primary mouse in vitro differentiated MTs, mouse liver, and brain sections. This revealed clear NE staining exclusively in muscle, but not other cell types (Figure 3C). Thus, even though these two proteins are widely expressed, they are NETs, at least among the tissues tested, only in muscle. NET39 (not tested here) was reported to specifically target to the NE in muscle (Liu et al., 2009). Hence, all four proteins are muscle-specific NETs and would only contribute their gene/chromosome-repositioning activity from the NE in muscle.

### Global Analysis of NET-Directed Gene Positioning and Expression

To identify genomic loci specifically recruited by the muscle-specific NETs, the generation of DamID maps from NET-Dam methylase fusions was attempted. However, these were unsuccessful, possibly because the methodology requires frequent methylated GATC sites within 2 kb of one another, which might be too short for the more specific interactions expected to be involved in tissue-specific gene repositioning. However, as genes relocating to or from the periphery tended to change expression, we reasoned that depletion of gene repositioning NETs would diminish gene expression changes in the MTs, thus revealing their genomic repositioning targets. Therefore, stable shRNA KD C2C12 lines for each NET were generated, differentiated into MTs, and subjected to gene expression analysis (Figure 3D).

Largely distinct sets of genes changed expression in KD lines for NET39, Tmem38a, and WFS1 (Figure 3E). Tmem214 KD (data not shown) exhibited considerable overlap with the other NETs, suggesting it affected gene repositioning through an indirect mechanism and therefore it was not considered further. Each NET affected expression of 15%–20% of all genes that changed expression in wild-type myogenesis (Figure 4A). When considered together, NET39, WSF1, and Tmem38a affected 37% of all genes that normally change in myogenesis. Conversely, ∼70% of genes altered by any individual NET changed expression during wild-type myogenesis (Figure 4B). Hence, NET depletion disproportionally affects the expression of distinct subsets of myogenic genes. Moreover, the changes induced by NET depletion were distinct from those previously reported in a MT-only KD of the transmembrane nucleoporin gp210 that had a profound effect on myogenesis (Figure S5) (D’Angelo et al., 2012). Postulating that gene expression defects resulted from a loss of NET-dependent repositioning in KD MTs, myogenic changes in gene expression and log2(Lamin B1/Dam) signal intensities were contrasted for genes within IP and PI regions (Figure 4C). This revealed a striking correlation in the directionality of changes in gene position and expression. Considering all genes changing significantly in both data sets, ∼70% had changes in the expected direction, i.e., IP correlating with repression and PI with activation (χ^*2*^ p = 1.6 × 10^−22^). Notably, the expression changes tended to be comparatively weak for the ∼30% of genes with repositioning in the unexpected opposite direction (KS test p = 2 × 10^−4^).

The genes that were altered in expression by NET KD were next overlaid onto these plots (Figures 4D–4F). 88% of gene expression effects induced by NET39 depletion were in a direction that countered normal myogenic changes. Genes normally repressed in MTs were upregulated with NET39 KD (yellow), while those normally induced were downregulated with NET39 KD (blue) (Figure 4D). Similar behavior was observed in ∼60% of myogenic IP and PI genes affected by WFS1 and Tmem38a KDs (Figures 4E and 4F).

### Muscle NETs Direct Myogenic Gene Repositioning and Repression

**Figure 5:**
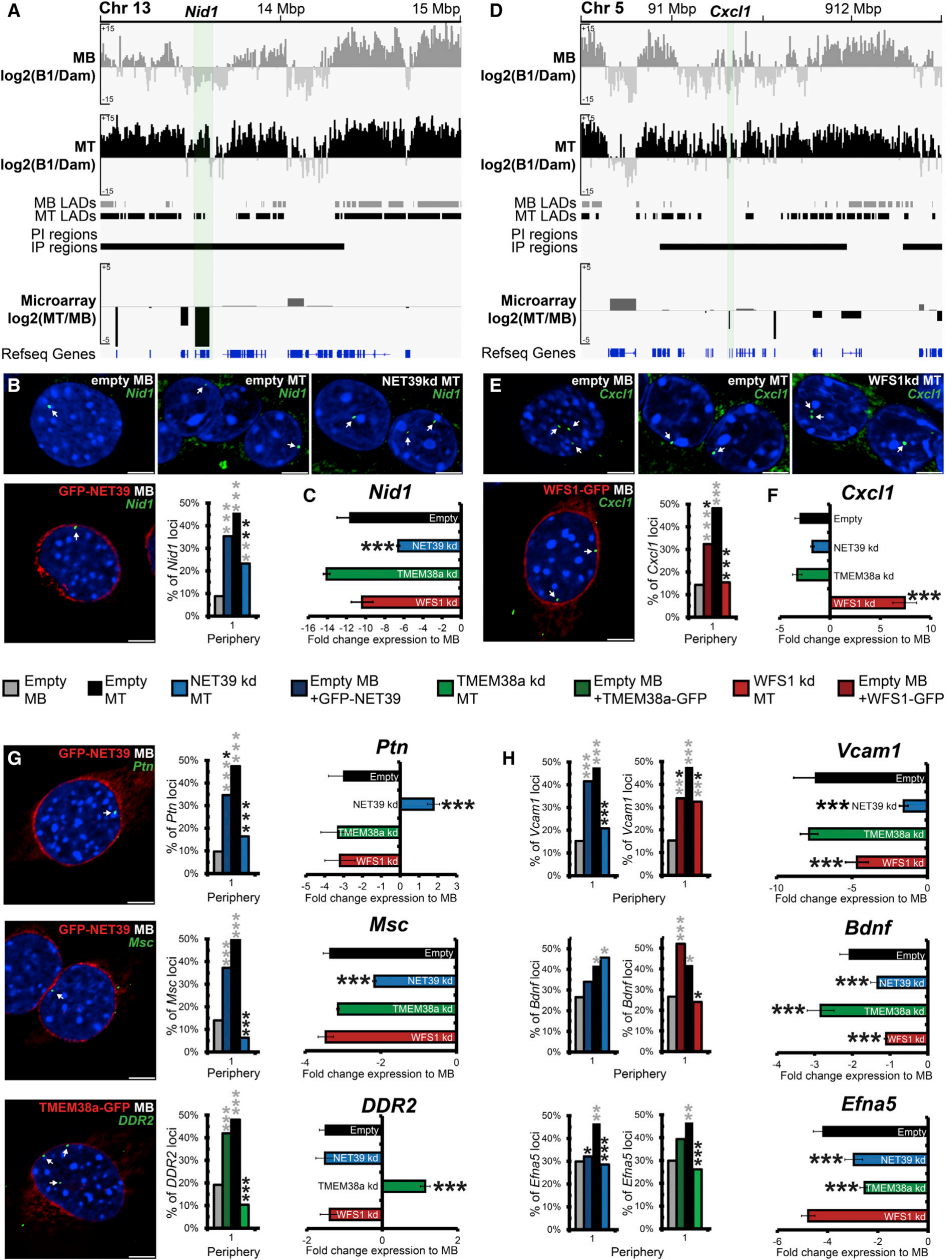
FISH and Microarray Analysis in NET KDs Confirms NET-Directed Repositioning and Expression Regulation (A and D) Genome browser views of myogenic DamID and gene expression changes of the *Nid1* (A) and *Cxcl1* loci (D). (B and E) FISH analysis of *Nid1* (B) and *Cxcl1* (E) loci position in control MBs and MTs, NET-depleted MTs, and NET-overexpressing MBs. (C and F) Histogram of *Nid1* (C) and *Cxcl1* (F) gene expression changes relative to MBs in control and NET-depleted MTs. (G and H) Similar microarray and FISH analysis of genes affected uniquely by depletion of a NET (*Ptn*, *Msc*, and *DDR2*) (G) and by depletion of multiple NETs (*Vcam1*, *Bdnf*, and *Efna5*) (H). For microarray data, error bars represent SD over three biological repeats, while statistics represent false discovery rates (FDR) between sample and an empty vector-treated control. For FISH, loci position was determined in 50–100 nuclei for each sample. For quantification statistics, the position of loci in the indicated sample was compared to the empty-vector MBs (gray asterisks) or MTs (black asterisks) using χ^2^ tests. ^∗^p < 0.05, ^∗∗^p < 0.01, and ^∗∗∗^p < 0.001. All FISH statistics are in Figure S6. The scale bars represent 5 μm.

Eight candidate genes from the intersect of the NET KD microarray and lamin B1 DamID data sets were chosen for further direct testing of NET-dependent peripheral gene repositioning by FISH. The *Nid1* gene is normally repressed early during myogenic differentiation, and the DamID data associated it with an IP region (Figure 5A). According to the microarray data, this repression of *Nid1* in MTs was strongly reduced in NET39 KD MTs (Figure 5C). FISH confirmed that *Nid1* is internal in MBs, peripheral in MTs, and fails to reposition to the periphery in NET39 KD MTs (Figure 5B). According to the microarray data, in the NET39 KD only ∼60% of the normal *Nid1* repression was achieved. This difference was significant (false discovery rate < 0.001), whereas *Nid1* repression was unaffected in WFS1 and Tmem38a KDs (Figure 5C). Critically, when NET39 was exogenously expressed in MBs, *Nid1* moved to the periphery (Figure 5B), clearly showing that NET39 is sufficient to direct *Nid1* repositioning even in the absence of myogenesis. However, this ectopically induced repositioning in MBs in the absence of differentiation was not associated with gene expression changes (Figure S6B), indicating that gene repositioning requires other aspects of the cellular milieu such as transcriptional repressors induced during differentiation for its effects on gene regulation.

The *Cxcl1* locus paralleled *Nid1*, except that it was specifically regulated by WFS1 (Figures 5D–5F). FISH for *Cxcl1* similarly confirmed the DamID results and determined that the gene repositioning depends specifically upon WFS1. Interestingly, WFS1 KD not only abolished MT-associated downregulation of *Cxcl1*, but also led to strong upregulation compared to MBs. Similar effects were observed for the *Ptn*, *Msc*, and *DDR2* loci that were dependent upon NET39 or Tmem38a for myogenesis-associated repositioning and repression (Figure 5G).

For *Vcam1*, *Bdnf*, and *Efna5*, more than one NET affected their expression (Figure 5H). Repression of *Vcam1* reached only ∼60% of normal levels with WFS1 KD and ∼30% with NET39 KD. The degree of diminished repression strongly correlated with the extent of loss of peripheral association in MTs, with WFS1 KD only partially impairing and NET39 KD effectively abolishing *Vcam1* repositioning. Both NET39 and WFS1 overexpression in MBs increased *Vcam1* peripheral positioning. A similar correlation for effect strength was observed for *Bdnf* and *Efna5*, except that NET39 had a stronger effect on expression than positioning of *Bdnf* and KDs were much stronger than overexpression in repositioning of *Efna5*. Statistics for the significance of these changes can be found in Figure S6A. Interestingly, *Cxcl1* and *Vcam1* do not target to the nuclear periphery in liver where, although expressed, WFS1 is exclusively ER localized (data not shown). Thus, every gene tested depended on the NET for myogenic repositioning and this correlated with changes in gene expression during myogenesis. Moreover, seven of eight genes tested could be redirected to the periphery in MBs just by overexpressing the NET in the absence of differentiation. In all cases, as for *Nid1*, inappropriate locus repositioning in MBs induced by NET overexpression did not alter gene expression (Figures S6B and S6C).

### NET39 Redirection of Locus Positioning

**Figure 6:**
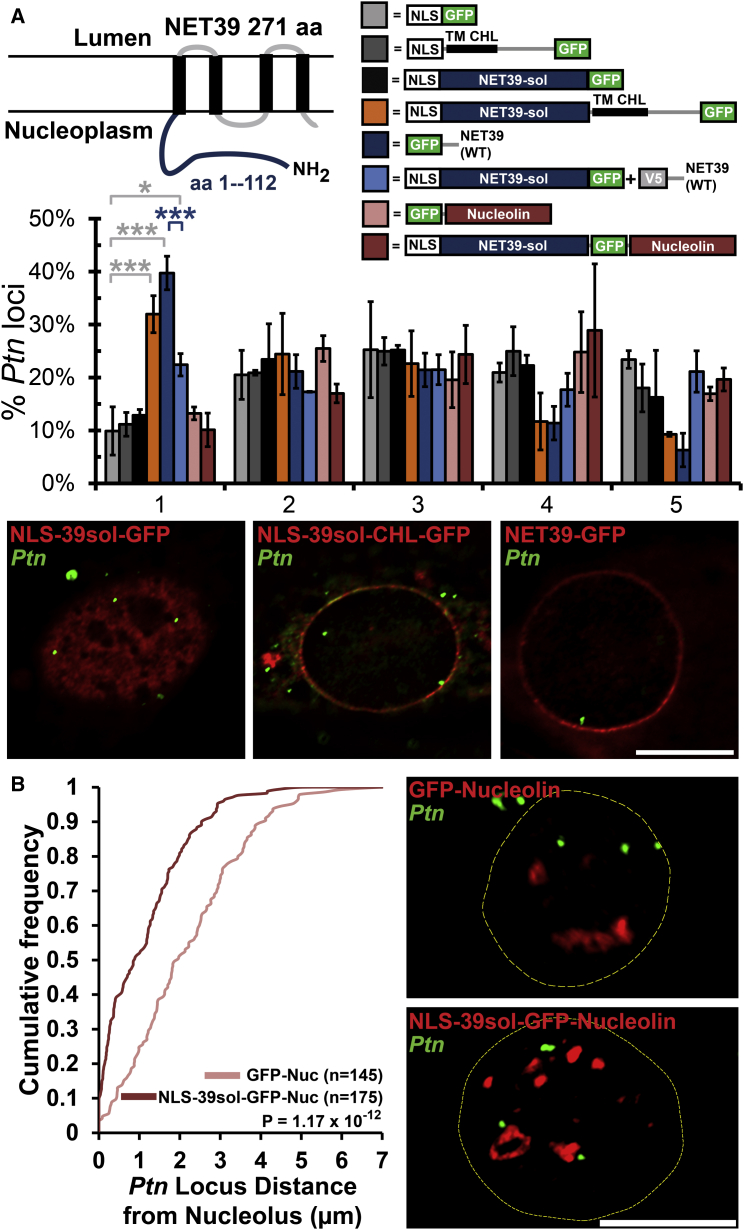
Direct Tethering Function of NET39 (A) Schematic diagrams of NET39 and fusion constructs with FISH analysis and representative images of *Ptn* loci positioning in indicated samples. The error bars represent SD of the mean over two biological repeats. ^∗∗∗^p < 0.001 and ^∗^p < 0.05 comparing samples to NLS-GFP expressing MBs by χ^2^ tests. (B) Representative images and cumulative frequency distribution of *Ptn* loci distance from the edge of nucleoli in indicated samples expressing either the NET39 soluble fragment fused to nucleolin or nucleolin-GFP over two summed biological repeats. For all images, *Ptn* is labeled green and GFP is labeled red. The scale bars represent 5 μm. See also Figure S7.

As the *Ptn* locus was specifically repositioned to the periphery during myogenesis by NET39, we next investigated the functional requirements for peripheral targeting. First, we tested if its N-terminal nucleoplasmic domain was sufficient to reposition the locus when expressed as a GFP-fused soluble fragment without the membrane anchor. Although this fragment alone was insufficient for repositioning, fusing it to a surrogate transmembrane domain from chicken hepatic lectin (CHL) could reposition the locus (Figure 6A). Interestingly, the GFP-fused NET39 soluble fragment could dominant-negatively compete the relocalization function of full-length NET39 tagged with the V5 epitope (Figure 6A). Similar results were also obtained with chromosome 8 repositioning as the readout (Figures S7A and S7B). This demonstrates that NET39-mediated repositioning is not due to its reported interaction with mTOR that was mapped within the soluble fragment (Liu et al., 2009). That only the NE-tethered NET39/NET39 fragment could reposition the locus strongly argues that NET39 functions as a direct tether for *Ptn* and other genomic loci.

To further test NET39 as a direct tether, the soluble fragment was redirected to a new nuclear location and cells analyzed for *Ptn* position. When the NET39 soluble nucleoplasmic fragment was fused to nucleolin, this fusion protein accumulated at the nucleolus and so did the *Ptn* locus (Figure 6B). In contrast, overexpression of nucleolin fused to GFP had no effect on *Ptn* localization. The number and area occupied by GFP-labeled nucleolar foci was unchanged between GFP-Nucleolin and NLS-39sol-GFP-Nucleolin, indicating increased *Ptn* association was not generally due to creation of larger or more numerous nucleoli (Figures S7C and S7D). Taken together, these data strongly indicate that NET39 functions as a tether that directly binds and recruits genomic loci to the nuclear periphery, where in context of differentiation they are subject to repression.

### Gene Repositioning Muscle NETs Are Critical for Myogenesis

**Figure 7:**
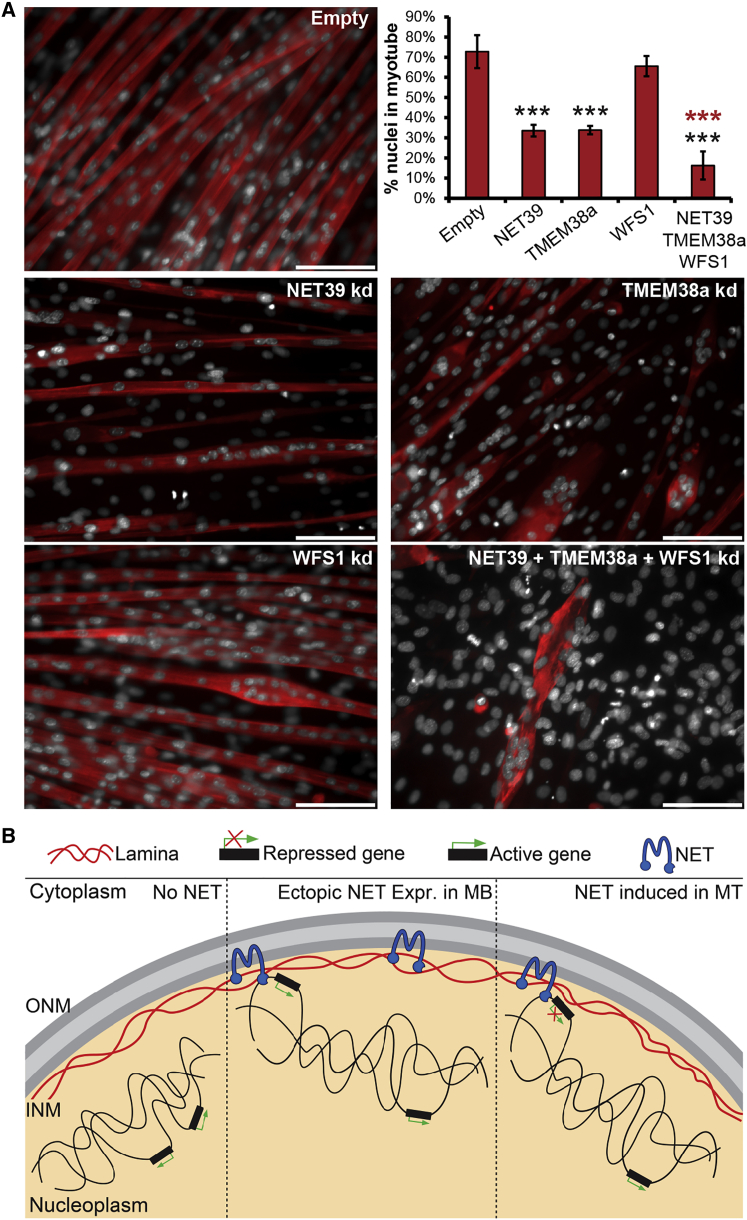
Gene Repositioning Muscle NETs Are Critical for Myogenic Differentiation (A) Representative images and quantification of the fraction of nuclei in MTs (marked by Myh1, red) in indicated samples. ^∗∗∗^p < 0.001 comparing the fraction of total nuclei present within MTs relative to empty vector-treated MTs by a χ^2^ test. The error bars represent the SD between 5–10 fields across three biological repeats. The scale bar represents 100 μm. (B) In MBs with low levels of muscle NETs, target genes are active in the transcriptionally permissive interior. Ectopic expression of these NETs in MBs results in peripheral gene targeting without repression. However, during differentiation, normal induction of the NET repositions the locus to the periphery concomitant with an increase in repression. Loss of the gene-repositioning NET results in both failure to reposition the locus and reduced repression (Inner [INM] and outer [ONM] nuclear membranes). See also Figure S7 and Movie S1.

Corroborating the key role of these gene-repositioning NETs in controlling myogenic gene expression, defects in MT formation were observed when NET39, Tmem38a, and WFS1 were knocked down individually and in combination prior to differentiation. MTs formed for all individual KDs, though they tended to be aberrantly thick and less ordered in the Tmem38a KD. For both NET39 and TMEM38a KD, a reduced myogenic index was observed as determined by the fraction of all nuclei within Myh1 stained MTs (Figure 7A). In contrast, MT formation was almost completely abolished in the triple KD, with the few remaining MTs being extremely thick, aberrantly shaped, and disordered. Phase contrast live cell imaging also revealed the presence of large vacuoles in Tmem38a- and WFS1-depleted MTs (Figure S7). Moreover, the kinetics of MT formation were reduced upon NET39 and TMEM38a KD and severely diminished in the triple KD (Movie S1). Hence, the loss of these three NETs, both individually and collectively, has profound consequences for myogenesis and the physical characteristics of any MTs formed.

## Discussion

The combination of DamID, transcriptomics, and FISH used here has clearly shown the existence of a mechanism for repositioning specific developmental genes by tissue-specific NETs. This mechanism is distinct from the previously described gene positioning due to recruitment of silenced chromatin to the periphery (Peric-Hupkes et al., 2010, Solovei et al., 2013).

### NET Targets Are Genes Requiring Fine-Tuned Regulation

We argue that this new type of gene positioning is focused on genes that need to be tightly regulated, often at critical times during differentiation and in a tissue-specific manner. Among those shown here, Musculin, encoded by *Msc*, occludes myoD binding sites to inhibit myogenesis and so must be very tightly repressed for myogenesis to progress (Lu et al., 1999). Nidogen 1, encoded by *Nid1*, is secreted into the extracellular matrix of early developing MTs. While important at this early time window, *Nid1* becomes repressed early in myogenesis and expressing Nidogen 1 after this point inhibits further differentiation (Neu et al., 2006). Vcam1, encoded by *Vcam1*, and its counter receptor Vla4 mediate interactions for satellite cell migration and MB-MT fusion, but Vla4 remains on myofibers, while Vcam1 disappears (Jesse et al., 1998, Rosen et al., 1992), suggesting it also must be tightly temporally regulated. Similarly, the *Ptn*, *Hgf, Efna5*, and *Bdnf* genes are all downregulated later in myogenesis, but are needed early for respective formation of neuromuscular junctions (Caruelle et al., 2004, Peng et al., 1995), migration of precursors (Dietrich et al., 1999), alignment of fusing MBs (Stark et al., 2011), and precursor functions (Mousavi and Jasmin, 2006). Likewise, the many cell-cycle genes thus regulated are needed in MBs to generate a sufficient number of cells to fuse into MTs, but must be tightly repressed once myofibers have formed. Additionally *Efna5*, *Cxcl1*, and *Ptn* are all induced upon muscle damage (Caruelle et al., 2004, De Paepe et al., 2012, Stark et al., 2011), suggesting that the peripheral tethering of genes at the NE places them under an additional regulatory control that can respond to muscle damage in order to reverse repression.

### Categories of Gene Regulation in Myogenesis

Use of the robust C2C12 differentiation system enabled our identification of IP and PI regions regulated by muscle-specific NETs. However, this only accounts for a subset of myogenic gene expression changes. The remaining IP and PI genes that repositioned and changed expression may be regulated by as yet unidentified muscle NETs or previously described heterochromatin and chromatin folding-dependent NE associations (Solovei et al., 2013). Genes that changed expression without repositioning are likely purely dependent upon transcription factor cascades or secondary/propagating effects (e.g., a repositioned gene encodes a transcriptional regulator). Separate functions for these NETs have been reported, the loss of which could contribute to gene expression changes. However, a reported function of WFS1 in ER stress responses is unlikely to be relevant as WFS1 was only in the NE in muscle (Fonseca et al., 2005). A calcium transport function reported for Tmem38a in the sarcoplasmic reticulum is similarly likely distinct from its NE role (Yazawa et al., 2007). Some NET39 gene regulation effects could be attributed to signaling changes from its reported function inhibiting mTOR activity (Liu et al., 2009). However, its ability to retarget *Ptn* to nucleoli strongly supports a more direct physical tethering function.

Loss or gain of peripheral chromatin associations will also inevitably influence internal genome organization. For example NET-tethering of a locus normally in an internal topologically associating domain (TAD) (Bickmore and van Steensel, 2013) would either move the TAD to the periphery or disrupt it. Alternatively, peripheral tethering could alter the genes available for long range inter-TAD interactions or transcription factories, thereby altering the expression of associated genes (Pombo and Dillon, 2015). This may explain how a NET involved in peripheral tethering also promotes altered expression of internally localized genes. Finally, IP and PI genes that repositioned without changing expression perhaps lacked transcriptional regulators in the muscle cells and may have moved as a consequence of the directed restructuring of adjacent genes rather than for their own regulation. This suggests that the effects described here are a central part of a much larger picture where transcriptional regulators bound to chromatin also play an important role and NET-directed positioning serves as an additional layer of regulation to further fine tune gene expression.

### Segregating Gene Positioning Effects from Other Aspects of Differentiation

The potential role of transcriptional regulators in the tethering nexus is prescient with regard to the central question of the actual contribution of peripheral positioning to gene regulation. The ability to disrupt the peripheral positioning of specific loci while other aspects of differentiation proceeded enabled distinguishing the contribution of positioning from other myogenic transcriptional regulatory cascades. Myogenesis proceeded with the KD of any individual NET and the genes were still partly repressed even when displaced from the periphery, indicating transcriptional repressors were present. Thus, this study has for the first time been able to distinguish for an endogenous locus, without artificial interventions, the relative contribution of peripheral positioning to gene repression, which generally seems to contribute between 1/3 and 2/3 of the total repression observed in normal myogenesis.

In contrast, no changes in expression were observed when NETs were exogenously expressed in MBs, indicating that repositioning to the NE is not intrinsically associated with repression. Rather, the combination of nuclear repositioning with other aspects of the cellular differentiation milieu allows optimal repression to be achieved once silencing is induced. We posit that tissue-specific NET-directed gene repositioning adds an extra layer of regulation to critical myogenic genes in order to better control their appropriate expression and repression at different myogenic stages (Figure 7B). The cumulative effect of these changes can yield repositioning of whole chromosomes. Determining NET binding partners on chromatin and the relative affinity of these and other NE-chromatin interactions will be important in the future. These NETs might also be important for NE-linked diseases. Lamins and several widely expressed NETs have been linked to Emery-Dreifuss and limb-girdle muscular dystrophies, but it is unclear how the tissue-specific muscle pathology is achieved. However, many myogenic genes are altered in these diseases (Bakay et al., 2006, Sáenz et al., 2008), suggesting the possibility that disrupted interactions with these muscle-specific NETs could underlie disease pathology. For now it is clear that these NETs direct tissue-specific patterns of gene positioning in myogenesis and contribute to the extent of gene repression from this peripheral tethering. Moreover, that 37% of all genes normally changing in myogenesis were affected by KD of three muscle-specific gene-positioning NETs, unequivocally establishes the importance of this type of regulation by tissue-specific NETs for muscle differentiation.

## Experimental Procedures

### Cells and Transduction

C2C12 MBs were cultured under ATCC-recommended conditions and induced to differentiate at 48 hr post-confluency by addition of DMEM with 2% horse serum. Differentiation media was replaced every 48 hr up to 144 hr post-induction. To inhibit contraction, 1 μM tetrodotoxin was added at 96 hr post-induction. VSV-G pseudotyped lentivirus’ encoding DamID, GFP, or pLKO constructs are described in Supplemental Information. 10 μg/ml protoamine sulfate was added during transduction to enhance efficiency. 1.5 μg/ml puromycin was used for selection of pLKO-encoding lentivirus transduced cells.

### FISH

C2C12 MBs and MTs were cultured on coverslips and fixed in 4% paraformaldehyde (PFA), 1× PBS. FISH was performed as described in Zuleger et al. (2013). Briefly, cells were permeabilized, treated with RNase A, and dehydrated with an ethanol series. DNA was denatured and captured in this state by a second ice-cold ethanol dehydration series. Coverslips were then annealed overnight to labeled BAC or whole chromosome probes. After washing, probes were visualized with Alexa Fluor-conjugated Streptavidin/anti-digoxigenin antibodies and total DNA visualized with DAPI. To identify overexpressing cells, they were stained with GFP antibodies before FISH. Coverslips were mounted in Vectashield (Vector Labs), images of nuclear midplanes acquired, and chromosome and gene positions determined using a macro run in Image-Pro Plus. This measures nuclear area from DAPI images, divides the area into five shells of equal area through eroding 20% of total area in steps from the DAPI-defined nucleus, determines the shell containing the gene spot or the chromosome intensity per shell, and sums this for each cell.

### Microarrays

Extracted total RNA from control MBs, MTs, and NET-depleted MTs was converted to biotin labeled-cRNA using the Illumina TotalPrep RNA Amplification Kit (Ambion, AMIL1791). For each analysis, three biological replicates were hybridized to MouseWD6 BeadChip Illumina whole genome expression arrays. Microarray data were quantile normalized, analyzed, and differentially expressed transcripts selected with a log2 ratio above 0.5 in absolute value using moderated F-statistics adjusted for a false discovery rate of 5%.

### DamID

DamID was performed as in Vogel et al. (2007). Briefly, C2C12 MBs and 72 hr differentiated C2C12 MTs were both separately transduced with Dam-Lamin B1- and Dam only-encoding lentiviruses over 24 hr. At 72 hr post-transduction, genomic DNA was extracted from trypsinized MBs and purified MTs and processed into libraries for next generation sequencing (Beijing Genomics). Sequenced reads were mapped to the mouse MM9 genome and the log2(Lamin B1/Dam) value determined for all genomic *DpnI* fragments in MBs and MTs. IP and PI regions were then identified by comparing the mean intensity differences between MT and MB log2(Lamin B1/Dam) values along a running 100 kb window. Regions with a mean MT/MB lamin B1 signal difference of 2-fold were then tested for significance against a randomized signal sample population using Fisher’s exact test over 1,000 iterations. Regions with p > 0.01 were disregarded.

### Immunofluorescence

The human muscle section from a control donor was obtained with informed consent of the donor and provided by Benedikt Schoser (Ludwig-Maximilians-Universität, München) through the Muscle Tissue Culture Collection at the Friedrich-Baur-Institüt. This collection is part of MD-NET and funded by the German Ministry of Education and Research and is partnered with EuroBioBank and TREAT-NMD. Local ethics approval was also obtained for use of human tissue from the University of Edinburgh School of Health in Social Science Research Ethics Panel. Mouse tissues were obtained in accordance with both University of Edinburgh and UK Home Office ethics approval and under Home Office License PPL 70/8175 to E.C.S.

## Author Contributions

Most experimental work, M.I.R.; EDL muscle preparation, R.C.; Bioinformatics, J.I.d.l.H., S.W., and A.R.W.K.; Tissue staining, P.L.T.; EM, D.G.B.; and Project design and writing, M.I.R. and E.C.S.

## Accession Numbers

The accession number for the MB and MT DamID and gene expression data reported in this paper is GEO: GSE80330.

## Supplemental Information

Document S1. Supplemental Experimental Procedures and Figures S1–S7

Table S1. Table of DamID and Gene Expression Changes in C2C12 Cells upon Myogenesis and NET-Depletion, Related to Figures 2, 3, 4, and 5 The table shows information for all genes represented on microarray for gene expression and DamID results presented as summary and raw data values. The table contains a key explaining the various parameters.

Movie S1. NET Depletion Significantly Alters the Kinetics of Myogenesis, Related to Figure 7 The movie shows the time-lapse of Empty-, NET39-, TMEM38a-, WFS1-, and combinatorial-shRNA treated MBs differentiating to myotubes over ∼5 days using the 10× objective of the IncuCyte ZOOM system. The kinetics of NET39, TMEM38a, and combinatorial KD MBs differentiation is severely impaired while the myotubes formed by TMEM38a and cells with the combinatorial KD are misshapen and poorly aligned.

Document S2. Article plus Supplemental Information
