# Supplementary material for: Tissue-Specific Gene Repositioning by Muscle Nuclear Membrane Proteins Enhances Repression of Critical Developmental Genes during Myogenesis
Source: Mol Cell. 2016 Jun 16;62(6):834–47. doi: 10.1016/j.molcel.2016.04.035 (PMC4914829; doi:10.1016/j.molcel.2016.04.035)
Supplement: Document S1. Supplemental Experimental Procedures and Figures S1–S7 [file mmc1.pdf]

Molecular Cell, Volume *62*

Supplemental Information

Tissue-Specific Gene Repositioning by Muscle Nuclear Membrane Proteins Enhances Repression of Critical Developmental Genes during Myogenesis

Michael I. Robson, Jose I. de las Heras, Rafal Czapiewski, Phú Lê Thành, Daniel G. Booth, David A. Kelly, Shaun Webb, Alastair R.W. Kerr, and Eric C. Schirmer

SUPPLEMENTAL FIGURES
Robson, de las Heras *et al*,.

A

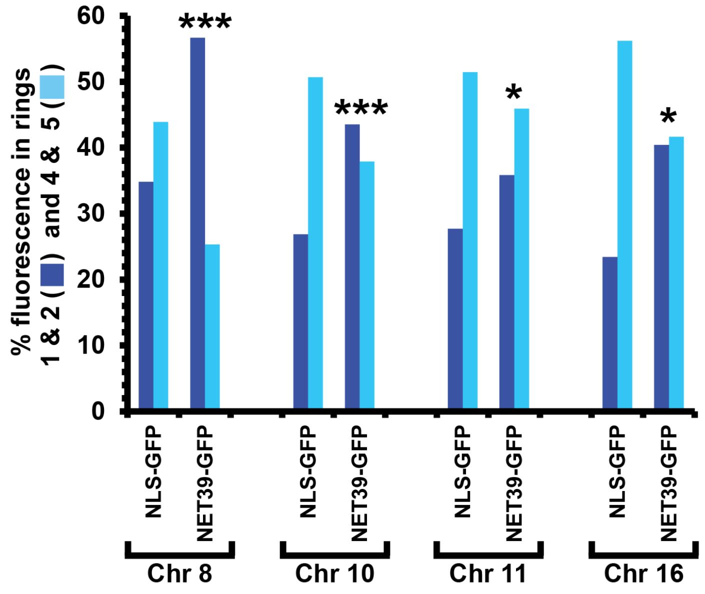

B

	NT MB	NT MT	NET39 shRNA MB	NET39 shRNA MT	NET39-shRNA MB+NLS-GFP	NET39 shRNA MB+GFP-NET39	Satellite cells	Myofibers
NT MB	NA	0.000	0.000	0.000	0.000	0.005	0.517	0.000
NT MT		NA	0.000	0.000	0.000	0.127	0.000	0.114
NET39 shRNA MB			NA	0.123	0.577	0.000	0.003	0.000
NET39 shRNA MT				NA	0.004	0.000	0.000	0.000
NET39-shRNA MB+NLS-GFP					NA	0.000	0.001	0.000
NET39 shRNA MB+GFP-NET39						NA	0.001	0.002
Satellite cells							NA	0.000
Myofibers								NA

P<0.05 P<0.01 P<0.001

Figure S1. Screen to Identify Substrates for NET39-mediated Chromosome Repositioning and Statistics for Figure 1E. GFP-NET39 was overexpressed in C2C12 MBs and the position of chromosomes 8, 10, 11 and 16 scored to determine if any are a substrate for NET39-mediated repositioning to the periphery. A. Quantitation of chromosome repositioning by NET39 overexpression in C2C12 MBs using the macro described in Figure 1D. *P < 0.05 and **P < 0.01 and ***P < 0.001 comparing the position of the chromosome in the GFP-NET39 overexpressing cells to the NLS-GFP overexpressing cells using KS tests. B. Summary table of KS tests for indicated comparisons from data in Figure 1 comparing NET39 overexpression and knockdown in both C2C12 myoblasts and myotubes. Values have been color coded based on degree of significance.

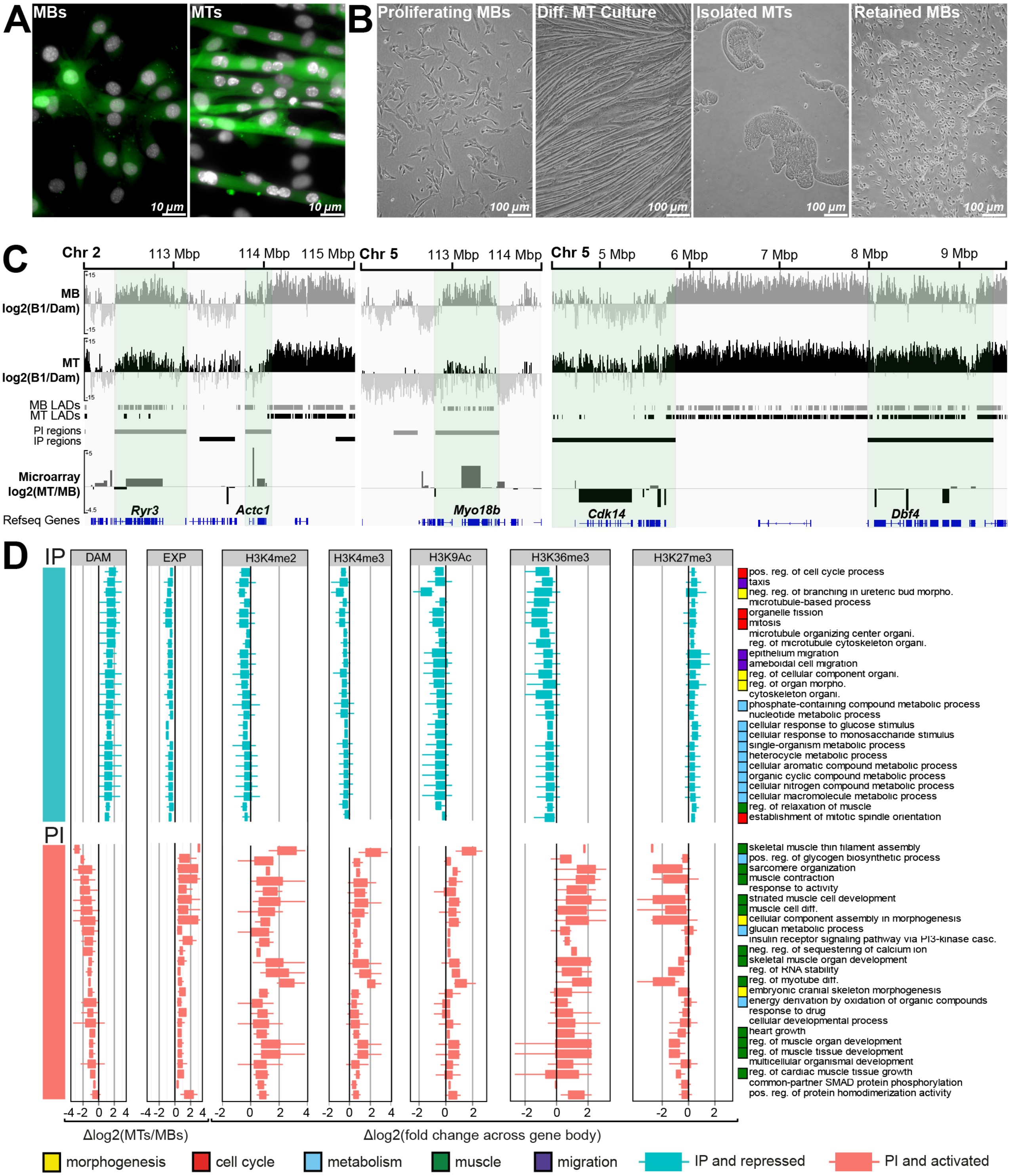

Figure S2. Example of MT isolation and DamID Genome Browser Views with ChIP-Seq Data and Variance, Related to Figure 2. A. Representative micrograph of C2C12 MBs and MTs transduced with a GFP-encoding lentivirus. B. Micrographs of cells pre- and post-isolation of a pure MT population. C. Genome browser views for genomic region surrounding indicated genes showing DamID signal intensities, identified LADs, IP and PI regions and microarray gene expression changes for MBs and MTs. D. Tukey boxplots (outliers not shown) displaying Δlog2 DamID, expression and Δlog(fold change) and indicated histone modifications values for genes within GO term categories significantly enriched in PI activated genes and IP repressed genes. Myogenic alterations to histone

modifications associated with transcriptionally active genes (H3K4me2, H3K4me3, H3K9Ac and H3K36me3) and the transcriptional repression-associated H3K27me3 were extracted from Asp et al., 2011. The Δlog2(DamID) value was calculated by subtracting the average log2(Lamin B1/Dam) value in a 100 kb window surrounding the gene in the MB sample from the MT sample. ChIP-seq values were determined for each histone modification by subtracting the average signal across the gene body in the MB sample from the MT sample.

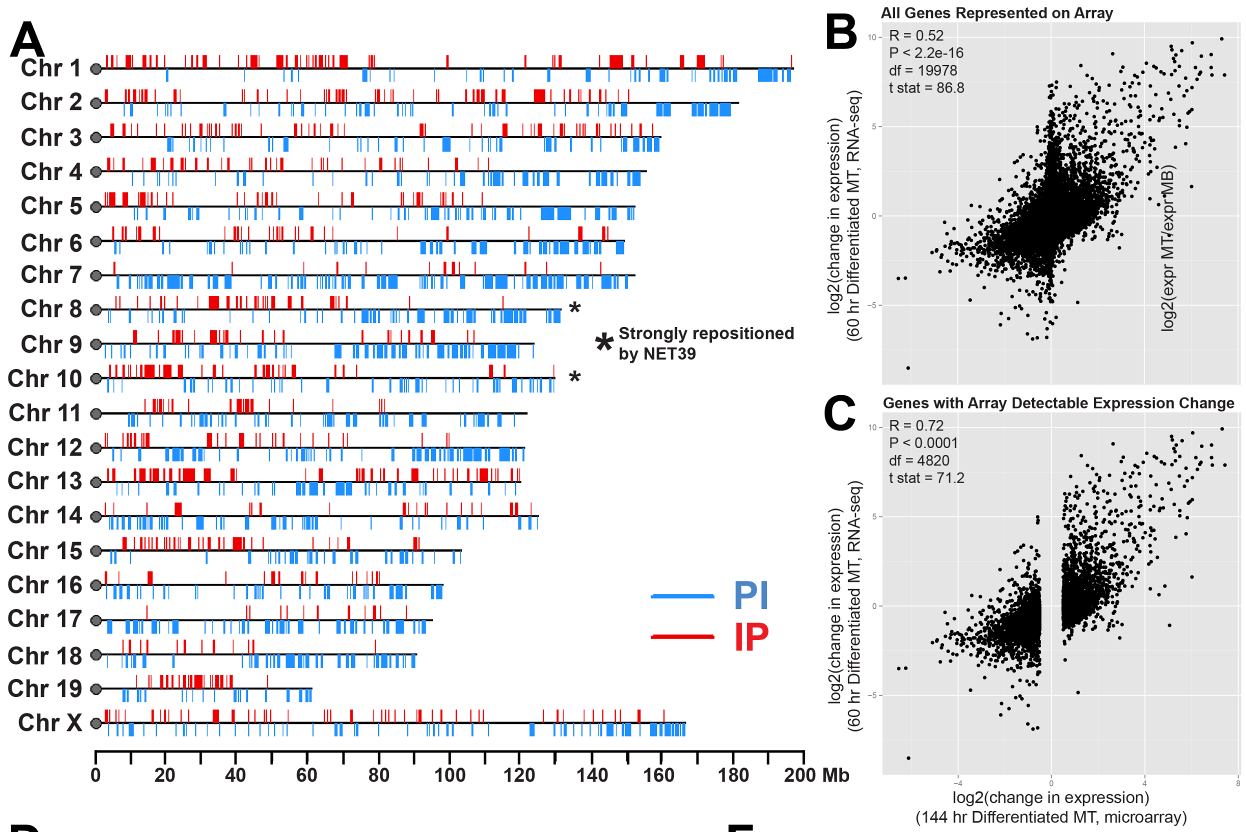

D IP Activated

GO term	Description	P-value	FDR q-value	Enrichment (N, B, n, b)	Genes
GO:0006334	nucleosome assembly	9.49E-04	1.00E+00	4.76 (2223,17,165,6)	Hist1h2bc, Hist1h2bj, Hist1h4i, Hist1h4n, Hist1h2bk, Hist1h1c, Hist1h2bm, Tspyl1

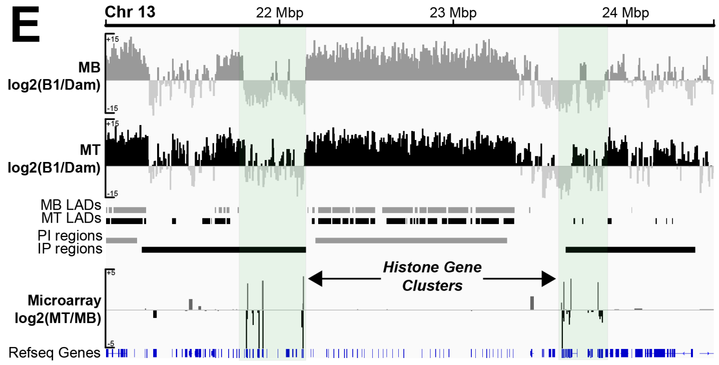

F PI Repressed

GO term	Description	P-value	FDR q-value	Enrichment (N, B, n, b)
GO:0000280	nuclear division	3.52E-06	2.72E-02	6.68 (2324,31,101,9)
GO:0051301	cell division	4.44E-06	1.72E-02	5.17 (2324,49,101,11)
GO:0007049	cell cycle	5.65E-06	1.45E-02	3.98 (2324,81,101,14)
GO:0007067	mitotic nuclear division	1.08E-05	2.08E-02	6.82 (2324,27,101,8)
GO:0048285	organelle fission	1.75E-05	2.70E-02	5.60 (2324,37,101,9)
GO:1903047	mitotic cell cycle process	2.96E-05	3.82E-02	4.29 (2324,59,101,11)
GO:0022402	cell cycle process	1.16E-04	1.28E-01	3.25 (2324,92,101,13)
GO:0006487	protein N-linked glycosylation	3.58E-04	3.46E-01	10.23 (2324,9,101,4)
GO:0016072	rRNA metabolic process	8.77E-04	7.52E-01	8.37 (2324,11,101,4)

G

P-value' is the enrichment p-value computed according to the mHG or HG model.

FDR q-value' is the correction of the above p-value for multiple testing using the Benjamini & Hochberg (1995) method. For the ith term (ranked according to p-value) the FDR q-value = (p-value * number of GO terms) / i.

Enrichment (N, B, n, b) is defined as follows:
N - is the total number of genes
B - is the total number of genes associated with a specific GO term
n - is the number of genes in the top of the user's input list or in the target set when appropriate
b - is the number of genes in the intersection
Enrichment = (b/n) / (B/N)

Figure S3. DamID Idiograms, Microarray and RNA-Seq Comparison, and Details of GO-term Analysis in Figure 2. A. Idiograms displaying the distribution of IP and PI regions with altered NE-association during myogenesis. * represents chromosomes which were found to be highly significantly repositioned by NET39 overexpression in C2C12 myoblasts as shown in Fig. S1. B. Comparison of C2C12 myoblast to myotube changes in gene expression detected in published RNA-Seq data from 60 hours of differentiation (Yue et al, 2014) vs our microarray analysis for 144 hour differentiated C2C12 myotubes. When the change in expression for all genes represented on the array is contrasted to the change detected by RNA-seq, a Pearson correlation of 0.52 is obtained. C. However, 75% of genes detected as “not changing” by the microarray were below the detectable microarray signal threshold, as determined from the distribution of log2(raw signal) of included negative controls (data not shown). Hence, when only comparing genes displaying altered expression during differentiation by microarray to the corresponding change detected by RNA-seq, a strong Pearson correlation of 0.72 is obtained. From this we conclude

the gene expression changes detected by our microarray are largely also detected in the RNA-seq dataset. A considerable source of the remaining deviation likely derives from the difference in time point of differentiation (144 hours for our microarray vs 60 hours for the published RNA-seq). R = correlation coefficient, P = P value, df = degrees of freedom, t statistic. D-G. GO-term Analysis for IP activated and PI repressed genes. D. List of GO-terms enriched in the set of genes which are recruited to the periphery and activated during myogenesis. Genes repositioning to the nuclear periphery concomitantly with activation display few enriched GO-terms. E. Genome browser view of the Hist1 gene cluster showing the majority of genes associated with the nucleosome assembly GO-term are located within two proximal IP regions. F. List of GO-terms enriched in the set of genes which are released from the periphery and repressed during myogenesis. In some cases genes associated with these processes require peripheral localization for transcriptional activation. G. Descriptions of table values described in D and F.

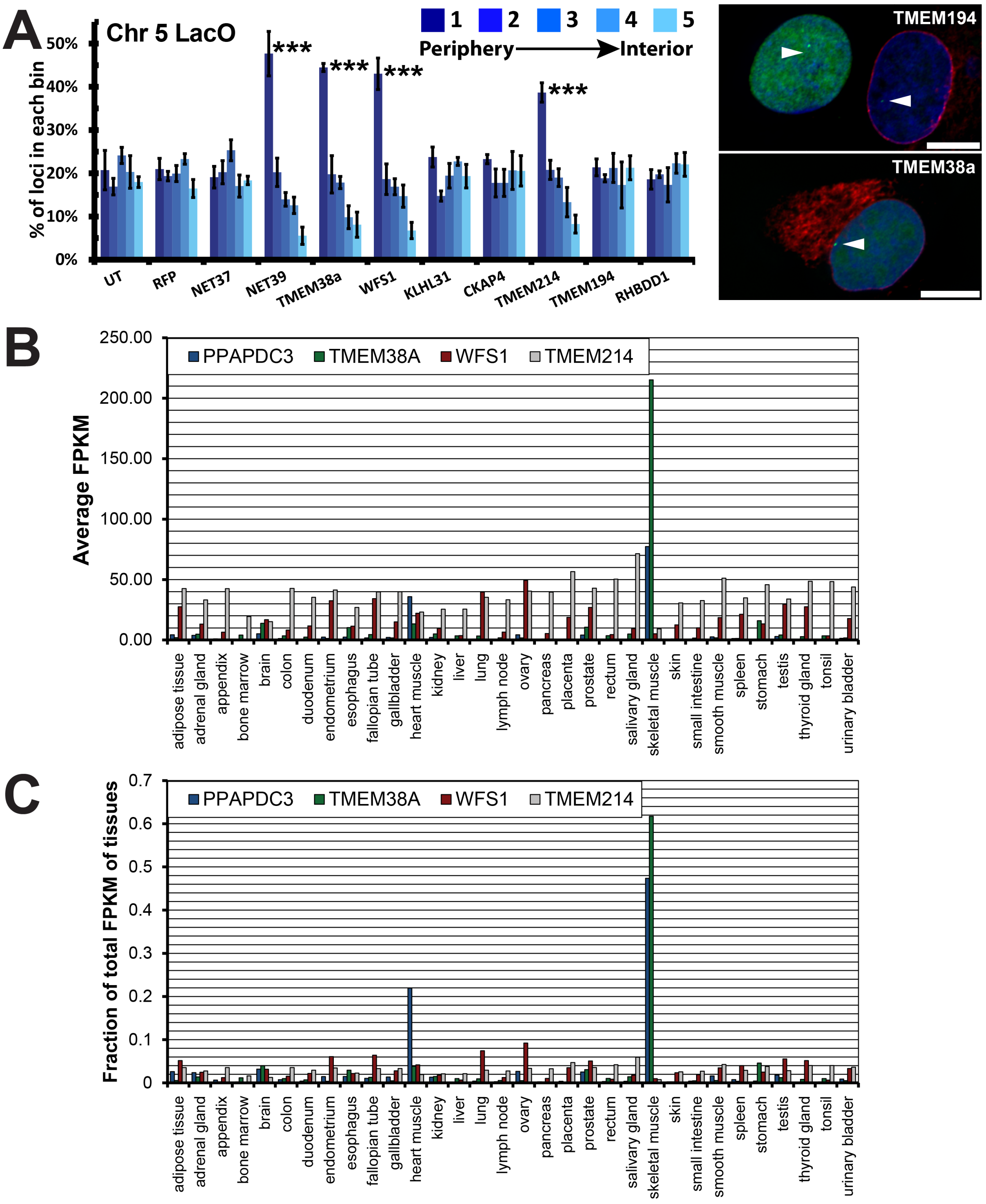

Figure S4. Screen to Identify Additional Muscle Repositioning NETs and Human Tissue RNA-seq Transcript Data, Related to Figure 3. A. Representative images and quantification of a lacO array integrated into chromosome 5 of HT1080 fibroblasts and used as a surrogate to measure chromosome-positioning changes when the cells overexpress NET-RFP constructs. The quantification identified 4 muscle NETs that significantly reposition chromosome 5 using the array as a marker for chromosome repositioning. Right image panels: lacI-GFP (green)

expression allows the lacO array to be visualized along with the NET-RFP protein (red) and DAPI (blue). ***$P < 0.001$ by comparing the peripheral to summed internal incidence of the array in the NET-transfected cells to the mRFP control using two way χ^2 tests. Error bars represent standard deviation of the mean across 3 biological repeats. B and C. Extracted transcript levels across human tissues from published RNA-seq data represented as average Fragments Per kb of transcript per Million mapped reads (FPKM) values or as a fraction of the total FPKM signal across all tissues (Uhlen et al., 2015). NET39 and TMEM38a are highly expressed in skeletal muscle while WFS1 and TMEM214 are more ubiquitously expressed, although targeting to the NE was restricted to muscle (Figure 3).

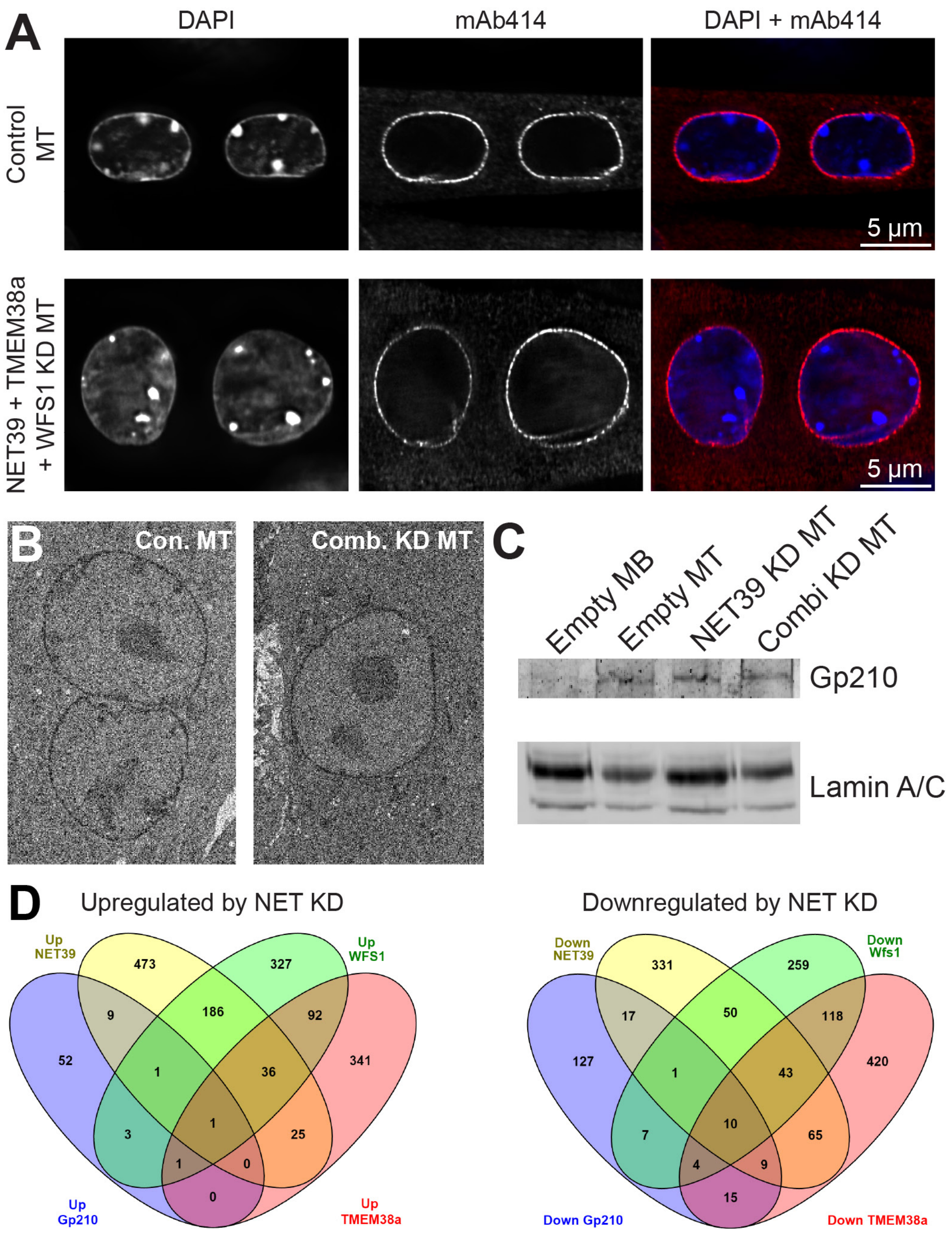

Figure S5. NPC and Gp210 (Nup210) are Unperturbed in Chromosome Repositioning NET Depleted Cells, Related to Figure 4. A. Representative micrographs demonstrating relative levels and distribution of NPCs by staining of FG-containing nucleoporins. B. 3D-View electron micrographs of Control and Triple-knockdown MT nuclei. In 20 nuclei observed for each sample, no increased NE ruptures or disruptions were observed. C. Western blot displaying unaltered levels of Gp210 induction and Lamin A/C expression in MBs, empty vector-treated MTs, NET39 shRNA-treated MTs or MTs co-treated with NET39, TMEM38a and WFS1 shRNAs. D. Non-proportional Venn diagrams displaying overlap between genes up- or down-regulated in Gp210-, NET39-, TMEM38a- or WFS1-depleted myotubes. The majority of genes affected by Gp210 are distinct from those affected by individual depletion of chromosome repositioning NETs. Gp210 gene expression data taken from D'Angelo et al 2012.

A

Nid1	Empty MB + GFP-NET39	Empty MT	NET39 kd MT
Empty MB	0.000	0.000	0.008
Empty MB + GFP-NET39		0.182	0.078
Empty MT			0.004

Ptn	Empty MB + GFP-NET39	Empty MT	NET39 kd MT
Empty MB	0.000	0.000	0.247
Empty MB + GFP-NET39		0.036	0.003
Empty MT			0.000

Msc	Empty MB + GFP-NET39	Empty MT	NET39 kd MT
Empty MB	0.000	0.000	0.120
Empty MB + GFP-NET39		0.141	0.000
Empty MT			0.000

DDR2	Empty MB + TMEM38a-GFP	Empty MT	TMEM38a kd MT
Empty MB	0.000	0.000	0.193
Empty MB + TMEM38a-GFP		0.541	0.000
Empty MT			0.000

Cxcl1	Empty MB + WFS1-GFP	Empty MT	WFS1 kd MT
Empty MB	0.001	1.000	0.000
Empty MB + WFS1-GFP		0.015	0.043
Empty MT			0.000

P<0.05 P<0.01 P<0.001

Vcam1	Empty MB + GFP-NET39	Empty MB + WFS1-GFP	Empty MT	NET39 kd MT	WFS1 kd MT
Empty MB	0.000	0.002	0.000	0.383	0.004
Empty MB + GFP-NET39		0.260	0.369	0.001	0.165
Empty MB + WFS1-GFP			0.042	0.040	0.881
Empty MT				0.000	0.023
NET39 kd MT					0.062

Bdnf	Empty MB + GFP-NET39	Empty MB + WFS1-GFP	Empty MT	NET39 kd MT	WFS1 kd MT
Empty MB	0.353	0.001	0.038	0.034	0.727
Empty MB + GFP-NET39		0.013	0.280	0.234	0.184
Empty MB + WFS1-GFP			0.224	0.406	0.000
Empty MT				0.862	0.026
NET39 kd MT					0.016

Efna5	Empty MB + GFP-NET39	Empty MB + TMEM38a-GFP	Empty MT	NET39 kd MT	TMEM38a kd MT
Empty MB	0.677	0.094	0.002	1.000	0.604
Empty MB + GFP-NET39		0.309	0.028	0.725	0.371
Empty MB + TMEM38a-GFP			0.245	0.188	0.038
Empty MT				0.019	0.001
NET39 kd MT					0.732

B

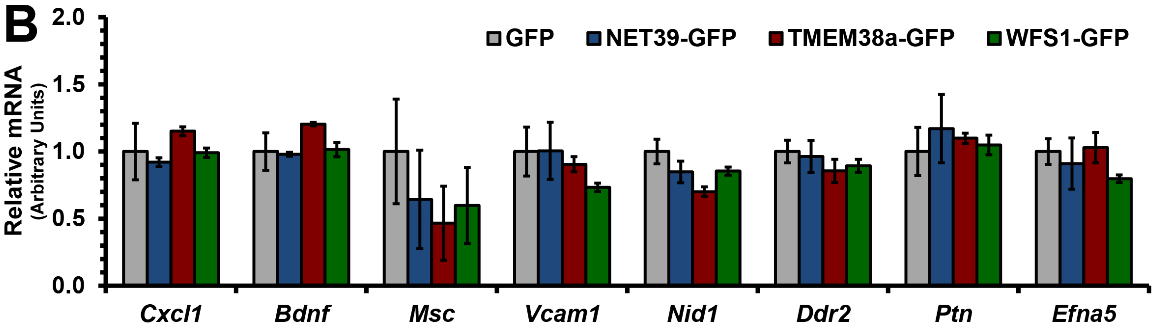

C

Two Tailed t-test	*Cxcl1*	*Bdnf*	*Msc*	*Vcam1*	*Nid1*	*Ddr2*	*Ptn*	*Efna5*
GFP:NET39-GFP	0.805	0.878	0.171	0.988	0.147	0.747	0.647	0.580
GFP:TMEM38a-GFP	0.644	0.181	0.082	0.677	0.054	0.177	0.653	0.797
GFP:WFS1-GFP	0.977	0.918	0.095	0.264	0.250	0.208	0.833	0.054

P<0.05
P<0.01
P<0.001

Figure S6. Summary of FISH Statistics for Figure 5 and qPCR of NET-Regulated Genes in C2C12 MBs. A. All statistical comparisons to determine the significance of repositioning for each gene calculated by χ^2 tests with degree of significance highlighted. Tests compared the peripheral to summed internal incidence of the locus in the indicated samples. B. qPCR of NET-regulated genes in MBs stably overexpressing GFP-tagged NETs. For each gene tested here, one of the NETs is normally needed for its repositioning and silencing at the periphery in MTs. However, the NETs could also reposition the genes to the periphery in MBs in the absence of differentiation when stably overexpressed (see Figure 5). However, NET-induced peripheral targeting in the absence of differentiation is is insufficient to repress target genes. As a control, samples were normalized to TMEM194, a NET that had no effect in the repositioning assay (Figure 3). Error bars represent standard deviation across 3 biological repeats. C. Table of statistical comparisons to determine the significance of gene expression changes for each gene calculated by Two Tailed t-test. No significant differences were observed.

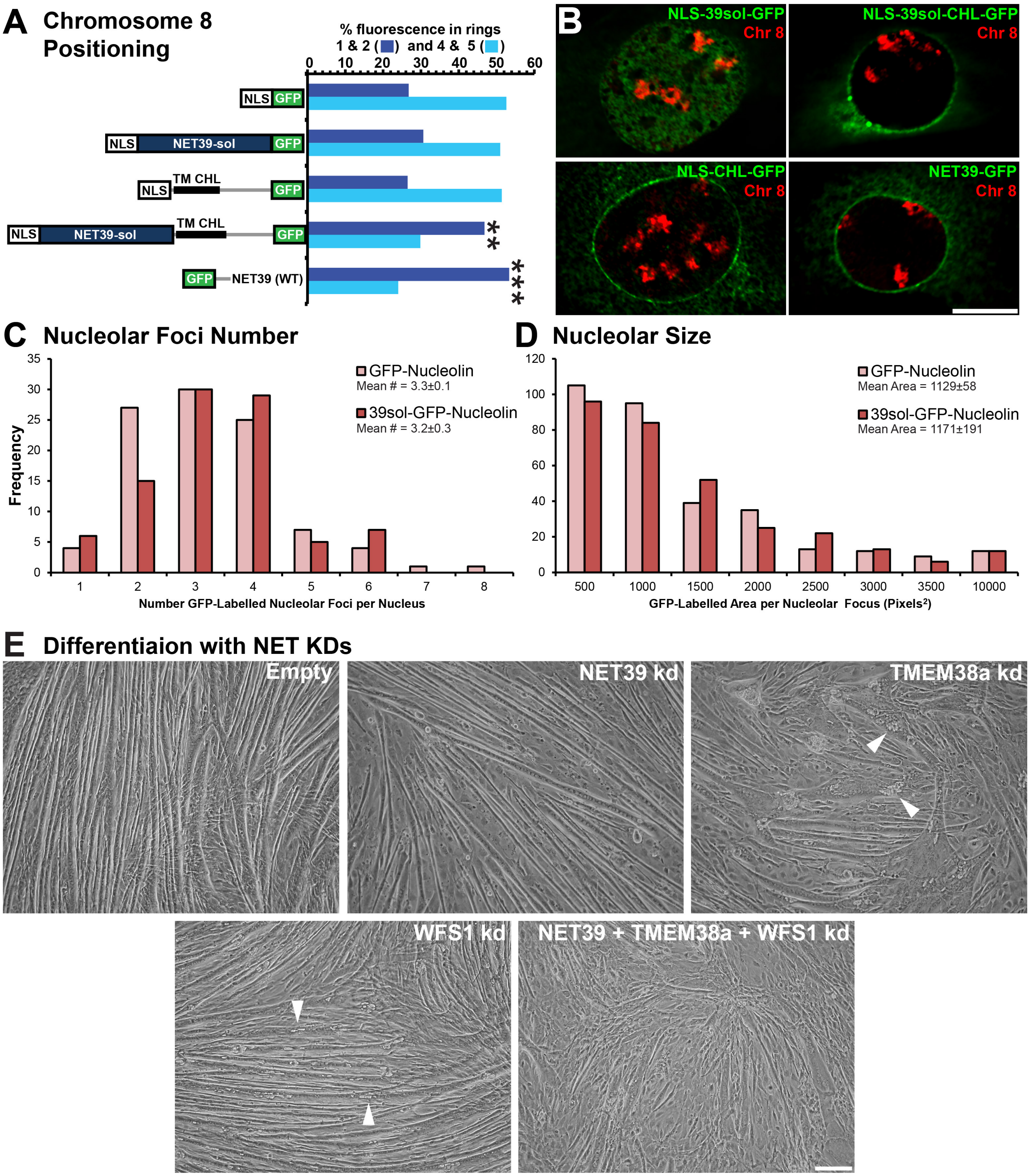

Figure S7. NET39sol Domain Construct Effects on Chromosome and Gene Position and Differentiation of NET KD MBs, Related to Figures 6 and 7. A and B. Re-anchoring the NET39sol domain rescues NET39 chromosome 8 repositioning, related to Figure 6. A. Quantification of chromosome 8 position across 50-100 cells in indicated conditions. Chromosome 8 is repositioned to the periphery only when the 39sol domain is anchored to the periphery. Scale bar is 5 µm. $^{**}P < 0.01$ and $^{***}P < 0.001$ comparing the position of the chromosome in the indicated overexpressing cells to the NLS-GFP overexpressing cells using KS tests. B. Example images of chromosome 8 position in NET39-shRNA MBs expressing the indicated constructs. C and D. The Number and Size of GFP-labelled Nucleoli Foci is Unaffected in NET39sol-GFP-Nucleolin Expressing Cells. C. Frequency histogram of the number of

nucleolar foci per nucleus with average size. D. Frequency histogram of the distribution of areas for GFP-labelled nucleolar foci. In both C and D the error margin for a given mean number or the area of a given nucleolar foci reflects the standard deviation between the means of two biological replicates. E. NET-depletion significantly perturbs the morphology and extent of differentiation of MTs, Related to Figure 7. Micrographs of knockdown MTs. Confluent plates of control MBs and NET39, TMEM38a and WFS1 knockdown MBs were induced to differentiate for 6 days. Control MBs form elongated, multinucleated MTs that align in linear arrays. Although NET39-depleted MTs were normal in appearance they were fewer in number. TMEM38a-depleted MTs were smaller and contained vacuoles indicative of large scale damage (indicated by arrows). WFS1-depleted MTs had less of a pronounced phenotype but also displayed visible vacuoles. Combinatorial NET39, TMEM38a and WFS1-depletion massively impaired myogenesis, generating large deformed myotubes that were significantly reduced in number suggesting the loss of each NET has an additive effect. Scale bar 100 µm.

Supplemental Movie 1. NET Depletion Significantly Alters the Kinetics of Myogenesis, Related to Figure 7. Time-lapse of Empty-, NET39-, TMEM38a-, WFS1- and combinatorial-shRNA treated MBs differentiating to myotubes over ~5 days using the 10x objective of the IncuCyte ZOOM® system. The kinetics of NET39, TMEM38a and combinatorial knockdown MBs differentiation is severely impaired while the myotubes formed by TMEM38a and cells with the combinatorial knockdown are misshapen and poorly aligned.

Supplemental Table 1. Table of DamID and Gene Expression Changes in C2C12 cells upon Myogenesis and NET-depletion, Related to Figures 2, 3, 4 and 5. Information for all genes represented on microarray for gene expression and DamID results presented as summary and raw data values. The table contains a key explaining the various parameters.

SUPPLEMENTAL EXPERIMENTAL PROCEDURES
Robson, de las Heras *et al,.*

Antibodies

Primary antibodies and their sources are listed below. For visualization of primary antibodies for immunofluorescence, donkey anti-mouse, anti-rabbit and anti-guinea pig secondary antibodies conjugated to a variety of Alexa Fluor® dyes were used (Molecular Probes, Invitrogen). For the detection of biotin- or digoxigenin-labelled probes in FISH experiments, streptavidin (Molecular Probes, Invitrogen) or anti-digoxigenin antibodies (Jackson labs) conjugated to Alexa Fluor® dyes were used, respectively. For Licor Western blotting, IRDye®-conjugated anti-mouse or anti-rabbit antibodies were used (Licor).

List of Primary Antibodies.

Antigen	Host	IF dilution	WB dilution	Band Size	Source
**H3**	Mouse	N/A	1:200-1:1000	17 kDa	Abcam (10799)
**Myh1**	Mouse	1:200	1:500-1:1000	200 kDa	Sigma (M1570) clone My-32
**Lamin A/C**	Guinee pig	1:200	1:1000	72 kDa	(Schirmer et al., 2001)
**Lamin B1**	Guinee pig	1:400	1:1000	66 kDa	(Schirmer et al., 2001)
**NET39**	Rabbit	N/A	1:200	30 kDa	Proteintech (20635-1-AP)
**TMEM38a**	Rabbit	1:100	1:200	30 kDa	Millipore (06-1005)
**WFS1**	Rabbit	1:50	1:200	100 kDa	Proteintech (11558-1-AP)
**TMEM214**	Rabbit	1:50	1:200	70 kDa	Proteintech (20125-1-A)
**Dystrophin**	Mouse	1:50	N/A	271 kDa	Glenn Morris (MANDYS1(3B7))
**V5**	Mouse	1:200	1:200	N/A	Invitrogen (R960-25)
**GFP**	Rabbit	1:200	1:1000	N/A	Invitrogen (A-11122)
**RFP**	Mouse	1:200	1:1000	N/A	Generated by Dzmitry Batrakou
**NPC Mixture**	Mouse	1:500	N/A	N/A	Abcam (ab24609)
**Gp210**	Guinee pig	1:200	1:500	250 kDa	Greber (1990)

Plasmid Sources and Construction

Expression constructs for human NET39/PPAPDC3, Tmem38a, WFS1 and Tmem214 were cloned previously by the Schirmer laboratory (Wilkie et al., 2011; Zuleger et al., 2011). Notably, the NET39 variant used in those works and in this one is a longer splice variant than that in the original paper describing this NET (Schirmer et al., 2003). NET depletion experiments employed the pLKO shRNA lentiviral vectors TRCN0000081370 (5’-AGGCTTCATCATCGGCTACTT-3’), TRCN0000124329 (5’-GCCTTCTGTTTCTGTTCTGTA-3’) and

TRCN0000248037 (5'ATGCCGAGTGTCATAAGAAAT3') targeting NET39, TMEM38a and WFS1, respectively (Sigma). The empty and non-target shRNA vectors SHC001 and SHC002 were used as reference controls (Sigma). To stably overexpress NETs in the absence of differentiation, GFP, GFP-NET39, TMEM38a-GFP or WFS1-GFP were cloned into a modified pLVX-TRE3G lentiviral vector (Clontech). In this vector, GFP constructs are doxycycline-inducible in pLVX-TET3G treated cells. Doxycyclin-inducible puromycin resistance is achieved simultaneously via an intervening IRES. The lentiviral packaging constructs psPAX2 and pMD2.G were a gift from Justina Cholewa-Waclaw (Adrian Bird Laboratory, WTCCB, Edinburgh).

For nuclear envelope retargeting experiments the 24-131 amino acid fragment containing the previously published transmembrane of chicken hepatic lectin (CHL) (Soullam and Worman, 1993) was inserted into the NLS-39sol-eGFP N3 construct described in Zuleger et al,. 2013. For nucleolin retargeting experiments the NET39 soluble fragment was inserted into a previously published GFP-nucleolin vector (Takagi et al., 2005). pSVK3-3 CHL was a gift from the Worman lab, and GFP-nucleolin a gift from Michael Kastan (Addgene plasmid # 28176).

Primary Myofiber and Satellite Cell Isolation

Myofibers and satellite cells were isolated from the Extensor Digitorum Longus (EDL) muscle of 6-8 week old male J6 mice as described by Moyle & Zammit and demonstrated visually by Pasut et al. (Moyle & Zammit, 2014; Pasut et al, 2013). Briefly, four 6-8 week old male J6 mice (Central Bioresearch Services, University of Edinburgh) were sacrificed by cervical dislocation and the EDL muscle extracted for dissociation into pre-warmed DMEM supplemented with 0.2% collagenase Type IV (Gibco, 17104-019) and antibiotics for 1.5 – 2 h at 37°C. Individual myofibers were dissociated from the muscle body by titration and separated from the remaining collagenase. For FISH, myofibers were transferred onto coverslips coated in matrigel (Gibco, A1413202), left to adhere for 4 h and then fixed. To isolate satellite cells, myofibers were plated in matrigel-coated 6 well plates for 72 h in DMEM supplemented with 20% FBS, 10% horse serum (Gibco), 1% chick embryo extract and 10 ng/ml basic Fibroblast Growth Factor (PeproTech, 450-33B), 100 units/ml penicillin, 100 μg/ml streptomycin and 10 μg/ml gentamicin. During this time associated satellite cells migrated onto the matrigel-coated plate and proliferated. To remove contaminating fibroblasts cells were trypsinized, pelleted and resuspended in satellite cell medium in a 35 mm dish to settle on the substratum. As fibroblasts but not satellite cells adhere efficiently in the absence of matrigel, after 15 min the media containing any non-adhered satellite cells was removed and cells re-plated. For FISH, satellite cells were then cultured and fixed on matrigel-coated coverslips. To generate MTs, satellite cells cultured on matrigel-coated coverslips were induced to differentiate at confluency using differentiation media as described for C2C12 MBs. Primary MTs were then fixed in 4% formaldehyde, PBS and stained as described below for tissue sections.

Lentivirus Generation and Transduction

Lentiviruses encoding DamID or pLKO constructs were generated as described in (Salmon and Trono, 2007) with several modifications. Briefly, non-replicative lentiviruses were generated in ~6 million 293FT cells plated in a 8.5 cm diameter tissue culture plate by joint transfection of 2.8 μg pMD2.G, 4.6 μg psPAX2 and 7.5 μg of the construct-specific transfer vector using 36 μl lipofectamine 2000 in 3 ml Optimem as per the manufacturer's instructions. After 16 h 293FT media was replaced. 48 h later the virus containing supernatant was aspirated, cleared of cellular debris by centrifugation for 10 min at 3,500 rpm and followed by filtration through a 0.45 μm^2 low protein binding PES syringe filter (Millipore, SLHP003RS). Viruses were then concentrated by ultracentrifugation at 55,000 x *g* for 75 min at 4°C in a JA-25.5 rotor and resuspended in an appropriate volume of Opti-mEM. If not used immediately, aliquots were frozen at -80°C. Transduction was performed in the presence of 10 μg/ml protoamine sulphate. When required, selection was applied 48 h post transduction by addition of 1.5 μg/ml puromycin.

DamID

DamID was performed as described in (Vogel et al., 2007). Briefly, for undifferentiated samples 40,000 C2C12 MBs were plated 8 h before transduction onto 3.48 cm diameter tissue culture plates and then, following adhesion to the substratum, were transduced with $^{1}/_{10}$ of a Dam methylase encoding virus's preparation in the presence of 10 µg/ml protoamine sulphate in growth medium (Dulbecco's modified Eagle's medium (DMEM) supplemented with 20% fetal calf serum (FBS), 100 units/ml penicillin and 100 µg/ml streptomycin). Transduction was performed overnight, after which virus containing media was replaced and cells left in culture for a further 48 h.

For differentiated samples, 5 days prior to transduction, C2C12 MBs were plated onto 3.48 cm tissue culture plates at 45,000 cells/cm^2 in growth medium. 48 h later media was aspirated, cells were washed twice in PBS and differentiation media (DMEM, 2% horse serum (Gibco), 100 units/ml penicillin and 100 µg/ml streptomycin) was added. Media was then replaced 48 h later. Following 72 h of differentiation, $^{1}/_{2}$ of a DamID viral preparation was added in the presence of 10 µg/ml protamine sulphate and 1 µM tetrodotoxin (TTX) (Toxin technologies) to prevent MT contractions. After 24 h, viral containing media was replaced with fresh differentiation media and cells left to differentiate for a further 48 h. For both undifferentiated and differentiated samples, cells were harvested by trypsinization 72 h post-transduction. For differentiated MTs partial trypsinization was performed to selectively enrich the MT population. Briefly, differentiation media was aspirated and cells subsequently washed once in PBS. TrypLE™ Express trypsin diluted 1:5 in PBS was then incubated with washed cells for 1 min at 37°C or until MTs, but not mono-nuclear MBs, were released from the growth surface, after which differentiation medium was added to block further trypsin activity and cell detachment. MTs were then carefully extracted by aspirating the cell-containing supernatant after which plates were gently washed in further differentiation medium to gather remaining MTs. To further deplete the sample of MBs, MTs were then preferentially pelleted by limited centrifugation at 500 rpm for 1 min.

DamID sample processing was then performed as described in Vogel et al. Briefly, DNA was extracted from cells using the DNeasy tissue lysis kit (Qiagen) as per manufacturer's instructions. 2.5 µg of extracted DNA was then digested by *DpnI* (NEB) and, following heat inactivation of *DpnI,* was ligated to the DamID adaptor duplex (dsAdR) generated from the oligonucleotides AdRt (5'-CTAATACGACTCACATAGGGCAGCGTGGTCGCGGCCGA-GGA-3') and AdRb (5'-TCCTCGGCCG-3') after which DNA was further digested by *DpnII*. To amplify DNA sequences methylated by the Dam methylase, 5 µl of *DpnII* digested material was then subjected to PCR in the supplied buffer in the presence of the 1.25 µM Adr-PCR primer (5'-GGTCGCGGCCGAGGATC-3'), 0.2 mM dNTPs and 1X of the Advantage cDNA polymerase (Clontech, cat. no. 639105). PCR was performed as described below.

PCR program for DNA amplification in DamID.

Cycle	Denature	Anneal	Extend
1			68°C for 10 min
2	94°C for 3 min	65°C for 5 min	68°C for 15 min
3-6	94°C for 1 min	65°C for 1 min	68°C for 10 min
7-23	94°C for 1 min	65°C for 1 min	68°C for 2 min

Following PCR, the quality of amplified DNA was confirmed on 2.5% agarose gels. If samples were of sufficient quality DNA was purified on QIAquick PCR purification columns (Qiagen) and then concentrated to the required concentration by precipitation. An average of 6-8 PCR reactions were required per sample to generate the 2 µg of material required of next generation sequencing.

DamID Sequencing and Analysis

DamID sample libraries were prepared for next generation sequencing by fragmentation followed by ligation to sequencing adaptors. Libraries were then sequenced by 90 bp paired end (90PE) sequencing in reactions with 5 samples per well. As mitochondrial DNA also becomes Dam-methylated and muscle has many mitochondria, ~73% of MT reads were mitochondrial. Thus, 310 million total reads were engaged for the MT lamin B1-dam sample to achieve 80.6 million reads after subtracting mitochondrial sequences. Between 77-100 million reads were achieved for MT Dam and MB and MT Dam-Lamin B1 samples. DamID sequences were aligned to the mouse mm9 genome using the Burrows-Wheeler Aligner software bwa-mem (Li and Durbin, 2009). Subsequent processing was performed using R and Bedtools (Quinlan and Hall, 2010; R Development Core Team, 2010). MB and MT DamID data was quantified at a DpnI fragment level by counting the number of reads that overlapped each DpnI-flanked (GATC) genomic fragment for each pair of Dam-alone and Dam-LaminB1 samples, and expressed as a proportion of the total to account for variation in the number of reads. The log2 ratios between Dam-LaminB1 and Dam-alone were calculated for each DpnI fragment, and the resulting values quantile normalized in R using the BioConductor Limma package (Ritchie et al., 2015) to allow a more quantifiable sample comparison.

Lamina Associated Domains (LADs) Definition

To identify LADs, the peakfinder software SICER (Wu and Yao, 2013; Zang et al., 2009) was employed with the following parameters: redundancy threshold 1, window size 500 bp, gap size 0, fragment size 150 bp, effective genome fraction 0.75, FDR 0.05. This identified a large number of small peaks with variable density distribution, roughly corresponding to the intensity of the LADs. We considered a gene being in a LAD if it overlapped with at least one SICER peak.

Identification of Genomic Regions with Altered Peripheral Association

To identify genomic regions with differential frequencies of association with the periphery in MBs vs MTs, a statistical test was devised in which a moving window of 250 DpnI fragments (~100Kb) were run along each chromosome, with a shift of 20 DpnI fragments (~10Kb). From this the average DamID signal the difference between MTs and MBs was calculated within each window. Windows with a signal difference greater than 2-fold were flagged as potential differential regions. These regions were then tested for statistical significance compared to a shuffled sample (x1,000 iterations) using Fisher's exact test. The regions that passed with $p<0.01$ were termed PI or IP regions, to denote regions that exhibited a significant repositioning to the interior (PI) or to the periphery (IP) during differentiation from MBs to MTs.

Gene Ontology and ChIP-seq Analysis

Genes in the IP class with reduced expression and genes in the PI class with increased expression were analyzed for Biological Process and Cellular Compartment GO-term enrichment using the BioConductor package GOstats (Falcon and Gentleman, 2007). The log2 change in DamID and microarray gene expression values and fold enrichment in ChIP-seq values between MTs and MBs was determined for genes within each of the top 25 enriched terms for both IP and PI classes and plotted accordingly. For DamID the log2 change in lamin B1 signal intensity for each gene represented an average value calculated for a 100 kb window surrounding the gene. For ChIP-seq, the fold enrichment between MTs and MBs was determined across the whole gene body including introns and exons. For ChIP-seq there was only limited information available for C2C12 differentiation, with only H3K4me1, H3K4me2, H3K4me3, H3K27me3, H3K36me3, H3K9Ac, H3K18Ac and H4K12Ac datasets available. These datasets were extracted from GSE25308 and quantification of fold enrichment across gene bodies achieved as described previously (Asp et al., 2011).

RNA-seq Analysis

RNA-seq expression values were obtained from published ENCODE data (GSM929774 and GSM929775) for C2C12 differentiation by averaging the Fragments Per kb of transcript per Million mapped reads (FPKM) signal across exons for each gene available using Bedtools (Yue et al., 2014). For tissue-wide expression of NETs data were extracted from Uhlen et al., 2015.

RNA Extraction

RNA extraction was achieved using TRI-reagent (Invitrogen) as per the manufacturer's instructions. For differentiated C2C12 cells, MTs were isolated from undifferentiated MBs by partial trypsinization and centrifugation as described previously. The quality and concentration of the RNA was then assessed using a NanoDrop 2000c spectrophotometer and an Agilent Bioanalyzer (RNA 6000 Nano total RNA kit, Agilent). If not immediately used, RNA was stored at -80°C in separate aliquots to reduce freeze thawing.

Microarrays

RNA was labelled with biotin using the TotalPrep RNA Amplification Kit (Illumina) following the manufacturer's instructions. The quality of cRNA was then confirmed by an Agilent Bioanalyzer using the RNA 6000 Nano mRNA RNA kit (Agilent). Quality analysis and hybridization of the cRNA was performed at the Wellcome Trust Clinical Research Facility in Edinburgh. The subsequent microarray data were normalized using the free statistics package R (R Development Core Team, 2010). For each analysis, at least three biological replicates were hybridized to Illumina whole genome gene expression arrays (MouseWD6 BeadChip). These arrays have coverage of 48,804 transcripts according to the NCBI RefSeq database release 17 and UniGene release 188. Hybridizations were carried out using an Illumina Beadstation. Microarray data were quantile normalized and analyzed in the R environment using the Bioconductor package Limma (Smyth, 2005). Differentially expressed transcripts were selected with a log2 ratio above 0.5 in absolute value using moderated F-statistics adjusted for a false discovery rate of 5% (Benjamini and Hochberg, 1995).

Quantitative Real Time PCR (qPCR)

cDNAs were generated using the Thermoscript II RNAse H– Reverse Transcriptase as per manufacturer's instructions. Briefly, following annealing to a polyA primer at 65°C, 5 μg of total RNA was incubated with 1X Thermoscript RNAse H- reaction buffer, 10 U RNAsin, 10 mM DTT, 1 μM dNTPs mix and 200 U Thermoscript II RNAse H– Reverse Transcriptase. Tubes were vortexed, centrifuged briefly, and incubated at 42°C in a thermocycler with the heated lid at 80°C for 2 h. After heat inactivation of the reverse transcriptase by a 15 min incubation at 70°C, RNA was removed by incubating samples with 1 μL of 7 mg/ml RNAse A for 45 min at 37°C. cDNA was diluted with 90 μl of water and the reactions stored at -20°C.

qPCR Program for Lightcycler 480.

Target (°C)	Acquisition mode	Hold (mm:ss)	Ramp rate (°C/s)	Acquisition (per °C)
**Preincubation, 1 cycle**				
**95**		05:00		
**Amplification, 40 cycles**				
**95**		00:10		
**56**		00:01		
**51**		00:015		
**72**	Single	00:21		
**Melting curve, 1 cycle**				
**95**		00:05		
**65**	Continuous	01:00	2.2	
**97**			0.19	3
**Cooling, 1 cycle**				
**22**			2.2	

For qPCR, 20 µl reactions containing 8.4 µl of diluted cDNA, 800 nM forward and reverse primers and 1x LightCyclerR 480 SYBR Green I Master were carried out in a 96-well LightCycler 480 Multiwell Plate. Polymerase chain reactions were carried out in a LightCycler 480 using the program detailed above. Primers used are listed in below. Expression data was analysed using LightCycler 480 Software v1.5.0.39. Primers for real-time PCR were designed using the IDT RealTime PCR web tool with default parameters except for amplicon size, which was set to 100 bp minimum, 150 bp optimum and 200 bp maximum.

List of qPCR Primers.

Gene	Species	Forward primer (5'-3')	Reverse Primer (5'-3')
**Ptn**	Mouse	AATGTGACCTCAATACCGCC	TCTCCTGTTTCTTGCCTTCC
**Vcam1**	Mouse	AGCAAAGACAGGAGACATGG	CAGTAGAGTGCAAGGAGTTCG
**Bdnf**	Mouse	ACCAGGTGAGAAGAGTGATG	AGTGTCAGCCAGTGATGTC
**Cxcl1**	Mouse	ACCCAAACCGAAGTCATAGC	GGACCCTCAAAAGAAATTGTATAGTG
**Msc**	Mouse	CTACGAGGACAGCTATGTGC	GAGAAGGTCCAGAATCCAGTG
**Nid1**	Mouse	CTCCAGTATCCTTTCGCTGTG	AGCAGTAATTGTGGCCTTGG
**Efna5**	Mouse	TTGGCAATCCTACTGTTCCTC	GTTAGGTGGATCTCTGGTGTTC
**Ddr2**	Mouse	CTGTCGGATGAGCAGGTTATC	CAGCTTATACACAGAGTCGGG
**TMEM194**	Mouse	TGACCCCAAACTCTTCCTTG	CCTACCAGGATGACGTAAATGG

Fluorescence In Situ Hybridization (FISH)

For FISH experiments C2C12 MBs and MTs were cultured on coverslips which were washed in PBS prior to fixation in 4% para-formaldehyde, 1X PBS for 10 min at room temperature. Satellite cells and primary myofibers were cultured on matrigel cultured coverslips and fixed identically. After aging coverslips for several days, cells were permeabilized for 6 min with 0.2% Triton-X-100 in PBS, followed by 3 washes in PBS. If antibody staining was required coverslips were blocked with 2% BSA prior to sequential incubations with primary and secondary antibodies. After washing, antibodies were fixed for 45 s in 2% paraformaldehyde, PBS. Cells were next pre-equilibrated in 2X SCC and treated with RNase A (100µg/ml) at 37ºC for 1 h. Following washing in 2X SCC, cells were dehydrated with a 70%, 85% and 100% ethanol series. Coverslips were then air dried, heated to 70ºC and submerged into 85ºC preheated 70% formamide, 2X SSC (pH 7.0) for 21 min. A second ethanol dehydration series was then performed using -20ºC 70% ethanol for the first step. Coverslips were air dried and 150-300 ng biotin-/digoxigenin-/fluorophore-labelled probe was added in hybridization buffer (50% formamide, 2X SSC, 1% Tween20, 10% Dextran Sulphate) containing 6 µg human Cot1 DNA (Invitrogen) and sheared salmon sperm DNA and incubated at 37°C for 24 h in a humidified chamber. For gene specific FISH, probes were generated from BACs by end labelling while chromosome paints were purchased pre-labelled. After incubation, the coverslips were washed four times for 5 min each in 4X SSC at 50ºC followed by four times for 5 min each in 0.1X SSC at 65ºC. Coverslips were then pre-equilibrated in 4X SSC, 0.1% Tween-20 and blocked with 4% BSA before incubating for 1 h at room temperature with Alexa Fluor®conjugated-Steptavidin/anti-dioxigenin antibodies and 4,6-diamidino-2 phenylindole, dihydrochloride (DAPI) at 2 µg/ml. Coverslips were subsequently washed 3 times in 4X SSC, 0.1% Tween-20 at 37ºC and mounted on slides in Vectashield (Vector Labs). Mouse chromosome 8 and human chromosome 5 were purchased from Metasystems and Cambridge Bioscience, respectively. BACs were ordered as indicated below.

List of FISH probes.

Locus	Chr	BAC	Source
Ttn	2	BMQ379B2	Source Biosystems
Efna5	17	BMQ-206B15	Source Biosystems
Nid1	13	BMQ-452C6	Source Biosystems
Ptn	6	BMQ-358G8	Source Biosystems
Msc	1	CH29-615N9	Source Biosystems
DDR2	1	BMQ-350O7	Source Biosystems
Cxcl1	5	BMQ-214F8	Source Biosystems
Bdnf	2	BMQ-60G18	Source Biosystems
Vcam1	3	CH29-72H24	BACPAC
Chromosome 8	8	N/A	Metasystems (000000-0528-839)

Determination of FISH-labelled Gene and Chromosome Position

The position of the loci and chromosomes was determined using previously designed macros written in Visual Basic and run on Image Pro Plus (available on request). Briefly, the macro calculates the DAPI-identified nuclear cross-sectional area and then sequentially erodes 20% of this in 4 stages to generate 5 shells of roughly equal area. The shell containing the locus was then determined and the number of events in each shell summed. The percentage of total events in each shell was then calculated in excel. For chromosomes the fraction of chromosome in each bin was calculated. Due to the larger size of chromosomes bins 1 and 2 were termed peripheral and bins 4 and 5 were termed internal. Only cells which had the locus/chromosome in focus at the mid-plane of the nucleus were analyzed.

Tissue Sections and Immunofluorescence Staining

The tissue samples of gastrocnemius muscle were provided by Dr. Benedikt Schoser (Friedrich-Baur Institute, Dept. of Neurology at the Ludwig-Maximilians-University of Munich, Germany). Liver and Brain sections were isolated from male J6 mice sacrificed by cervical dislocation. Tissue blocks were snap frozen using liquid nitrogen in OCT mounting medium and mounted on cryostat chucks using OCT mounting medium (Tissue-Tek). Sections were cut to a thickness of 10 µm using a cryostat (Leica CM1900) and mounted on SuperFrost Plus (VWR) slides. Slides were immediately placed on dry ice and stored at -80°C. Primary myotubes stained were grown in tissue culture on matrigel-coated coverslips. For staining, sections were first equilibrated at RT and OCT mounting medium (Tissue-Tek) removed. Sections were incubated in blocking solution (1% Fish gelatine (G-7765, Sigma) in TBS + 0.1% Tween) for 30 min followed by primary antibodies overnight at 4°C. Sections were washed 3 x 5 min using TBS + 0.1% Tween and incubated with secondary antibodies and DAPI for 1 h and washed as previous. Sections were then mounted in Vectashield (Vector Laboratories).

3view Electron Microscopy

MBs were seeded and differentiated to MTsin glass-bottomed dishes (MatTek) for 6 days. MTs were then fixed (2% Paraformaldehyde, 2% Glutaraldehyde) and stained with heavy metals: reduced osmium tetroxide, tannic acid, osmium tetroxide, uranyl acetate and lead aspartate. Samples were dehydrated in a graded series of ethanol before embedding in Agar 100 resin. Following polymerisation at 60°C for 48 h, hardened resin samples were trimmed using an ultra-microtome and mounted onto a pinhead. Serial block-face EM was performed using a Gatan 3View. Full reconstruction of samples was achieved using 600 x 50 nm sections per sample.

Determination of MT Fusion Frequency

Empty vector- and NET shRNA-treated MBs were differentiated on coverslips for 6 days and subsequently fixed in 4% formaldehyde, PBS. All nuclei were then stained with DAPI while MTs were selectively stained using an anti-myosin heavy chain 1 (Myh1) antibody as described in the above sections. 5-10 fields across 3 biological replicates were then imaged and the number of nuclei with significant signal overlap with Myh1 staining were counted and determined to reside within MTs and those without were determined to represent MBs. The fraction of total nuclei present within MTs was then determined.

Microscopy

Images were acquired on a Nikon TE-200 microscope using a 1.45 NA 100x objective, Sedat quad filter set, PIFOC Z-axis focus drive (Physik Instrubments) and a CoolSnapHQ High Speed Monochrome CCD camera (Photometrics) run by Metamorph image acquisition software.

IncuCyte™ Visualization of MT Formation

Empty vector- and NET shRNA-treated MBs were plated in 6 well plates at confluency and induced to differentiate after 48 h by addition of differentiation medium in an IncuCyte ZOOM® system. MTs were then allowed to form over the next 4 days with media changed every 48 h. Cells were imaged every 30 min over 16 fields per well using the IncuCyte™ 10x objective. For Supplemental Movie 1 a single field per condition was selected and the collected images assembled into a movie using the IncuCyte ZOOM® system software.

Western Blotting

Protein samples were prepared from cells after directly lysing in TRIzol™ following the manufacturer's protocol. Protein concentration was quantified following resuspension in 20 mM Tris, pH 8.0, 1% SDS using the Pierce® BCA Protein Assay Kit. Cell lysates were separated on 8-12% Tris-glycine-SDS or Bis-Tris gels. Subsequently the gels were transferred onto nitrocellulose membranes (Odyssey 926-31092) by means of semi-dry transfer (BIO-RAD). After transfer the membrane was blocked in western blot blocking buffer (5% milk powder in PBS with 0.05% Tween-20) for 30 min. Subsequently, the membrane was incubated with the primary antibody diluted in Western blot blocking buffer at the dilutions indicated in the Antibodies section above for 60 min at room temperature or overnight at 4°C. Six washes in PBS, 0.05% Tween-20 were then followed by incubation with the secondary antibody conjugated to an IRDye® for 60 min at room temperature. After 6 washes in PBS, 0.05% Tween20, membranes IRdye®-conjugated antibodies were detected on a Li-Cor Odyssey Quantitative Fluorescence Imager.

SUPPLEMENTAL REFERENCES

Asp, P., Blum, R., Vethantham, V., Parisi, F., Micsinai, M., Cheng, J., Bowman, C., Kluger, Y., and Dynlacht, B.D. (2011). Genome-wide remodeling of the epigenetic landscape during myogenic differentiation. Proceedings of the National Academy of Sciences of the United States of America *108*, E149-158.

Benjamini, Y., and Hochberg, Y. (1995). Controlling the false discovery rate: a practical and powerful apporach to multiple testing. Journal Royal Statistical Society Series B *57*, 289-300.

Falcon, S., and Gentleman, R. (2007). Using GOstats to test gene lists for GO term association. Bioinformatics *23*, 257-258.

Greber, U.F., Senior, A., and Gerace, L. (1990). A major glycoprotein of the nuclear pore complex is a membrane-spanning polypeptide with a large lumenal domain and a small cytoplasmic tail. Embo Journal 9, 1495-1502.

Li, H., and Durbin, R. (2009). Fast and accurate short read alignment with Burrows-Wheeler transform. Bioinformatics *25*, 1754-1760.

Quinlan, A.R., and Hall, I.M. (2010). BEDTools: a flexible suite of utilities for comparing genomic features. Bioinformatics *26*, 841-842.

R Development Core Team (2010). R: A language and environment for statistical computing. (Vienna, Austria, R Foundation for Statistical Computing).

Ritchie, M.E., Phipson, B., Wu, D., Hu, Y., Law, C.W., Shi, W., and Smyth, G.K. (2015). limma powers differential expression analyses for RNA-sequencing and microarray studies. Nucleic Acids Reearch *43*, e47.

Salmon, P., and Trono, D. (2007). Production and titration of lentiviral vectors. Current protocols in human genetics / editorial board, Jonathan L Haines [et al] *Chapter 12*, Unit 12 10.

Schirmer, E.C., Florens, L., Guan, T., Yates, J.R.r., and Gerace, L. (2003). Nuclear membrane proteins with potential disease links found by subtractive proteomics. Science *301*, 1380-1382.

Schirmer, E.C., Guan, T., and Gerace, L. (2001). Involvement of the lamin rod domain in heterotypic lamin interactions important for nuclear organization. The Journal of Cell Biology *153*, 479-489.

Smyth, G.K. (2005). Limma: linear models for microarray data. In Bioinformatics and Computational Biology Solutions Using R and Bioconductor, R. Gentleman, V. Carey, S. Dudoit, R. Irizarry, and W. Huber, eds. (New York, Springer), pp. 397-420.

Soullam, B., and Worman, H.J. (1993). The amino-terminal domain of the lamin B receptor is a nuclear envelope targeting signal. The Journal of Cell Biology *120*, 1093-1100.

Takagi, M., Absalon, M.J., McLure, K.G., and Kastan, M.B. (2005). Regulation of p53 translation and induction after DNA damage by ribosomal protein L26 and nucleolin. Cell *123*, 49-63.

Uhlen, M., Fagerberg, L., Hallstrom, B.M., Lindskog, C., Oksvold, P., Mardinoglu, A., Sivertsson, A., Kampf, C., Sjostedt, E., Asplund, A., *et al.* (2015). Proteomics. Tissue-based map of the human proteome. Science *347*, 1260419.

Vogel, M.J., Peric-Hupkes, D., and van Steensel, B. (2007). Detection of in vivo protein-DNA interactions using DamID in mammalian cells. Nature Protocols *2*, 1467-1478.

Wilkie, G.S., Korfali, N., Swanson, S.K., Malik, P., Srsen, V., Batrakou, D.G., de las Heras, J., Zuleger, N., Kerr, A.R., Florens, L., *et al.* (2011). Several novel nuclear envelope transmembrane proteins identified in skeletal muscle have cytoskeletal associations. Molecular & cellular proteomics : Molecular Cellular Proteomics *10*, M110 003129.

Wu, F., and Yao, J. (2013). Spatial compartmentalization at the nuclear periphery characterized by genome-wide mapping. BMC genomics *14*, 591.

Yue, F., Cheng, Y., Breschi, A., Vierstra, J., Wu, W., Ryba, T., Sandstrom, R., Ma, Z., Davis, C., Pope, B.D.*, et al.* (2014). A comparative encyclopedia of DNA elements in the mouse genome. Nature *515*, 355-364.

Zang, C., Schones, D.E., Zeng, C., Cui, K., Zhao, K., and Peng, W. (2009). A clustering approach for identification of enriched domains from histone modification ChIP-Seq data. Bioinformatics *25*, 1952-1958.

Zuleger, N., Kelly, D.A., Richardson, A.C., Kerr, A.R., Goldberg, M.W., Goryachev, A.B., and Schirmer, E.C. (2011). System analysis shows distinct mechanisms and common principles of nuclear envelope protein dynamics. The Journal of Cell Biology *193*, 109-123.
