# Supplementary figures and images for: Tissue-Specific Gene Repositioning by Muscle Nuclear Membrane Proteins Enhances Repression of Critical Developmental Genes during Myogenesis
Source: Mol Cell. 2016 Jun 16;62(6):834–47. doi: 10.1016/j.molcel.2016.04.035 (PMC4914829; doi:10.1016/j.molcel.2016.04.035)

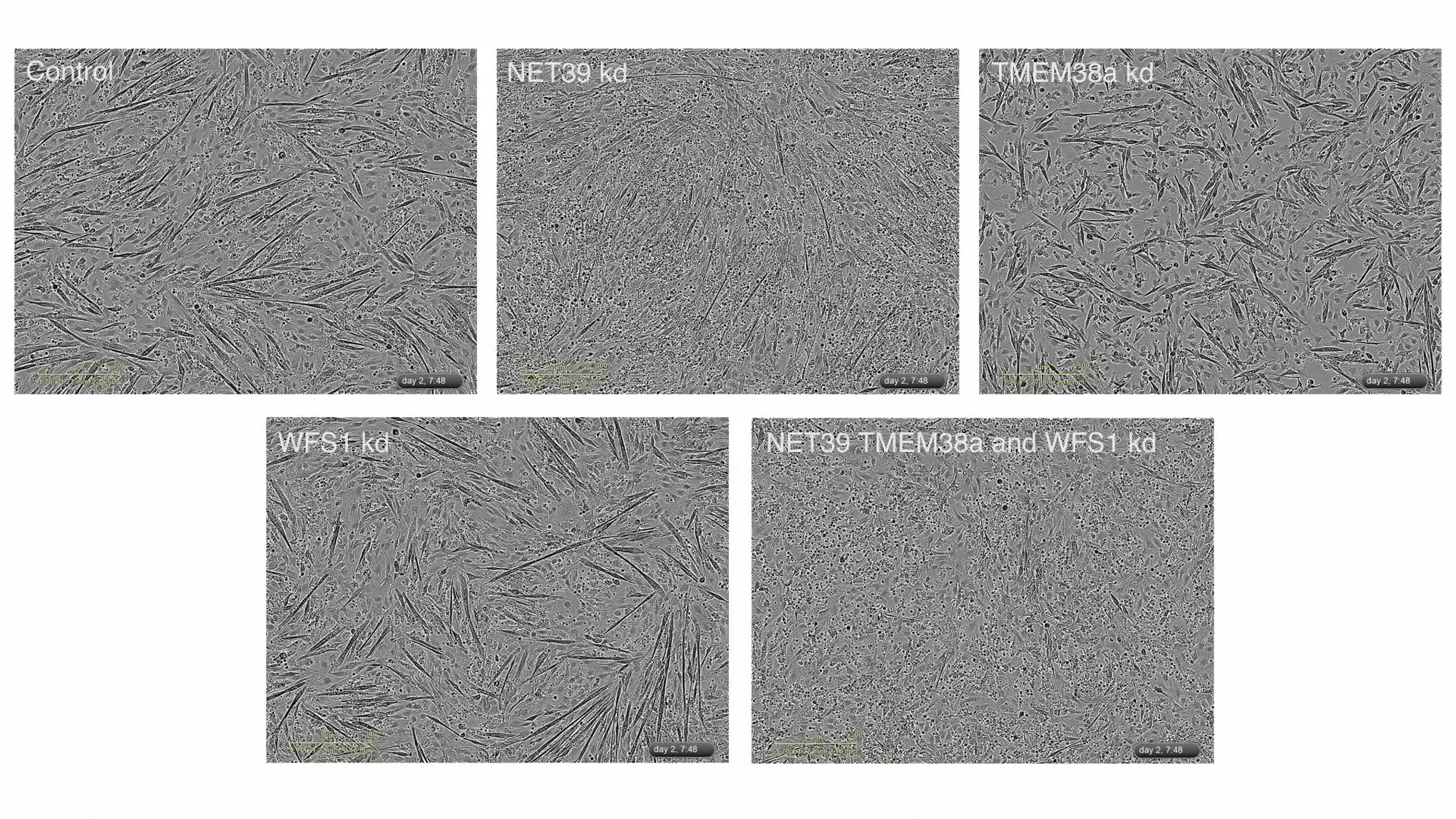

Supplement: Movie S1. NET Depletion Significantly Alters the Kinetics of Myogenesis, Related to Figure 7 — The movie shows the time-lapse of Empty-, NET39-, TMEM38a-, WFS1-, and combinatorial-shRNA treated MBs differentiating to myotubes over ∼5 days using the 10× objective of the IncuCyte ZOOM system. The kinetics of NET39, TMEM38a, and combinatorial KD MBs differentiation is severely impaired while the myotubes formed by TMEM38a and cells with the combinatorial KD are misshapen and poorly aligned. [file mmc3.jpg]
