# Supplementary material for: Tissue-Specific Gene Repositioning by Muscle Nuclear Membrane Proteins Enhances Repression of Critical Developmental Genes during Myogenesis
Source: Mol Cell. 2016 Jun 16;62(6):834–47. doi: 10.1016/j.molcel.2016.04.035 (PMC4914829; doi:10.1016/j.molcel.2016.04.035)
Supplement: Document S2. Article plus Supplemental Information [file mmc4.pdf]

Molecular Cell

Article

# Tissue-Specific Gene Repositioning by Muscle Nuclear Membrane Proteins Enhances Repression of Critical Developmental Genes during Myogenesis

## Graphical Abstract

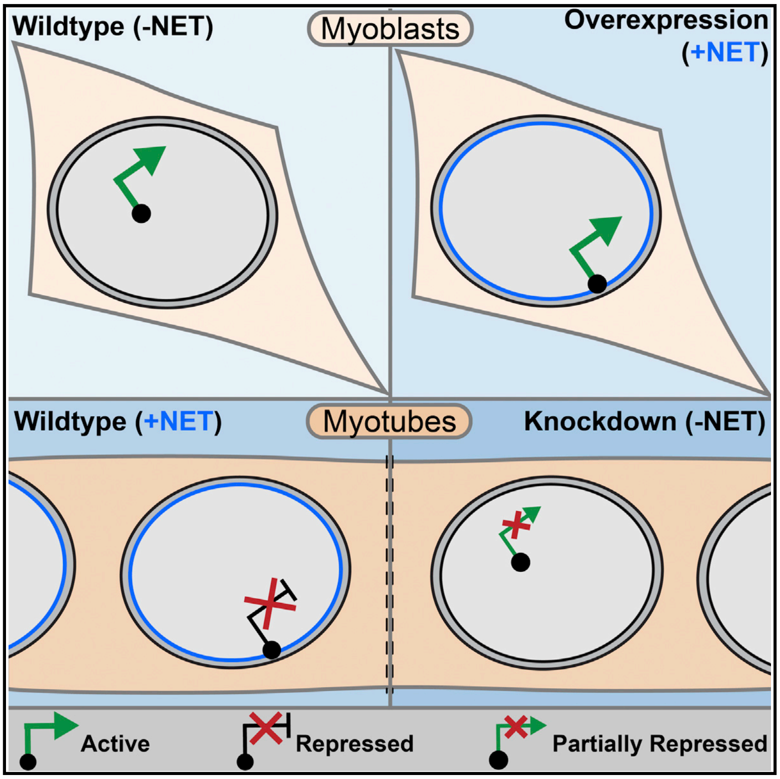


## Authors

Michael I. Robson, Jose I. de las Heras, Rafal Czapiewski, ..., Shaun Webb, Alastair R.W. Kerr, Eric C. Schirmer

## Correspondence

e.schirmer@ed.ac.uk

## In Brief

Muscle-specific nuclear envelope transmembrane proteins (NETs) optimize myogenic gene expression by physically recruiting genes to the periphery and enhancing their repression. Specifically manipulating the position of endogenous genes in myoblasts and myotubes indicates that peripheral localization enhances repression, but only in context of other changes in differentiation.

## Highlights

- Tissue-specific NETs direct repositioning of critical muscle genes during myogenesis
- Expression changes for NET-repositioned genes depend on cell differentiation state
- Isolating position from differentiation reveals its contribution to gene expression
- Three NETs together affect 37% of all genes normally changing in myogenesis

## Accession Numbers

GSE80330

Robson et al., 2016, Molecular Cell 62, 834–847
June 16, 2016 © 2016 The Authors. Published by Elsevier Inc.
http://dx.doi.org/10.1016/j.molcel.2016.04.035

Molecular Cell
Article

# Tissue-Specific Gene Repositioning by Muscle Nuclear Membrane Proteins Enhances Repression of Critical Developmental Genes during Myogenesis

Michael I. Robson,[1] Jose I. de las Heras,[1] Rafal Czapiewski,[1] Phú Lê Thành,[1] Daniel G. Booth,[1] David A. Kelly,[1] Shaun Webb,[1] Alastair R.W. Kerr,[1] and Eric C. Schirmer[1,*]
[1]The Wellcome Trust Centre for Cell Biology and Institute of Cell Biology, University of Edinburgh, Edinburgh EH9 3BF, UK
*Correspondence: e.schirmer@ed.ac.uk
http://dx.doi.org/10.1016/j.molcel.2016.04.035

## SUMMARY

**Whether gene repositioning to the nuclear periphery during differentiation adds another layer of regulation to gene expression remains controversial. Here, we resolve this by manipulating gene positions through targeting the nuclear envelope transmembrane proteins (NETs) that direct their normal repositioning during myogenesis. Combining transcriptomics with high-resolution DamID mapping of nuclear envelope-genome contacts, we show that three muscle-specific NETs, NET39, Tmem38A, and WFS1, direct specific myogenic genes to the nuclear periphery to facilitate their repression. Retargeting a NET39 fragment to nucleoli correspondingly repositioned a target gene, indicating a direct tethering mechanism. Being able to manipulate gene position independently of other changes in differentiation revealed that repositioning contributes $^{1}/_{3}$ to $^{2}/_{3}$ of a gene's normal repression in myogenesis. Together, these NETs affect 37% of all genes changing expression during myogenesis, and their combined knockdown almost completely blocks myotube formation. This unequivocally demonstrates that NET-directed gene repositioning is critical for developmental gene regulation.**

## INTRODUCTION

Repositioning certain developmentally important genes between the nuclear periphery and interior during differentiation correlates with changes in their expression state. For example, concomitant with their activation, the *MyoD, IgH*, and *Mash1* genes reposition from the periphery to the nuclear interior respectively during myogenic, B cell, and neuronal differentiation. Conversely, the *c-maf* locus repositions to the periphery as it is repressed during T cell differentiation (Wong et al., 2014; Zuleger et al., 2011). New genome-wide approaches have identified many developmentally important genes that change in their peripheral association during differentiation with correspondingly altered expression (Peric-Hupkes et al., 2010). These findings raise the questions: how are gene positioning patterns established and regulated during development, and, to what extent does altered nuclear position contribute to changes in a genes expression?

Although little is known about the regulation of specific developmental gene repositioning to the periphery, nuclear envelope (NE) proteins clearly direct establishment of genome-wide patterns of peripheral heterochromatin organization. Peripheral heterochromatin is disrupted in mice lacking the NE transmembrane protein (NET) LBR and lamin A (Solovei et al., 2013). Lamins also contribute to more specific gene repositioning in *C. elegans* muscle, but the mechanism remains elusive (Mattout et al., 2011). Similarly, in mouse fibroblasts, lamin B1 and the NET LAP2β function with transcriptional regulators to peripherally target the *IgH* and *Cyp3a* loci, but it is unclear how the *IgH* locus is specifically released in pro-B cells (Zullo et al., 2012). However, the near ubiquitous expression of lamins, LAP2β and LBR and the general nature of these interactions argue for other mechanisms to explain tissue-specific aspects of gene positioning.

Determining peripheral positioning's contribution to regulation of gene expression is stymied by the fact that gene repositioning in development is accompanied by changes in transcriptional regulators, epigenetic marks, and other genome changes associated with differentiation. Circumventing this by directing reporter genes to the NE in the absence of differentiation used artificial locus-NE protein interactions (Finlan et al., 2008; Kumaran and Spector, 2008; Reddy et al., 2008); however, these studies yielded inconsistent results concerning the effect of repositioning on the expression of tethered reporters. Random genome-wide insertions of reporter genes yielded significant repression when integrated into peripherally localized genome regions (Akhtar et al., 2013). More recently, TALEN-driven chromatin unfolding sufficed to release endogenous developmental genes from the periphery, suggesting position may in some cases reflect a gene's state of folding (Therizols et al., 2014). However, it is unclear if chromatin unfolding can drive repositioning for a range of developmental genes or if other more specific directed mechanisms normally function in development. Addressing these questions requires identification of the endogenous proteins responsible for peripheral gene localization and their manipulation in both differentiated and undifferentiated cells.

We recently identified several tissue-specific NETs that direct several chromosomes to the nuclear periphery in fibroblasts (Zuleger et al., 2013). We hypothesized that chromosome

 © 2016 The Authors. Published by Elsevier Inc.
This is an open access article under the CC BY license (http://creativecommons.org/licenses/by/4.0/).

repositioning reflects a summation of individual specific gene repositioning events directed by tissue-specific NETs during differentiation and that repositioning contributes to gene regulation. Combining transcriptome analysis in myogenesis with NET knockdowns (KDs) and global gene positioning changes determined by DamID (Vogel et al., 2007), we for the first time show the NET dependence of specific gene repositioning and its consequences for gene repression in myogenesis. Critically, the ability to specifically target the NET independently of other changes in myogenesis demonstrates that gene repositioning adds another layer to gene regulation during development.

## RESULTS

### NET39 Directs Chromosome 8 Repositioning In Myogenesis

NET39 is induced early in myogenesis (Liu et al., 2009) and could reposition a subset of chromosomes to the nuclear periphery in fibroblasts (Zuleger et al., 2013). This suggested that NET39—and potentially other muscle NETs—might direct gene and chromosome positioning changes during myogenesis. Accordingly, we investigated its contribution to chromosome positioning using the C2C12 system, in which proliferating mouse myoblasts (MBs) can be induced to differentiate into myotubes (MTs) (Yaffe and Saxel, 1977) (Figure 1A). Using whole chromosome fluorescence in situ hybridization (FISH), we first tested whether exogenous NET39 expression could increase peripheral localization of four chromosomes (8, 10, 11, and 16), finding chromosome 8 strongly affected (Figure S1A). Stable NET39 KD C2C12 lines were generated and transient NET39 overexpression was used to next test NET39 function during myogenesis. The target small hairpin (sh)RNA reduced NET39 protein levels by >90% in the MTs and to levels undetectable by western blot in the MBs (Figure 1B).

Chromosome 8 normally repositions from the interior in MBs to the nuclear periphery of MTs during myogenesis (Figure 1C, non-target shRNA). Repositioning was quantified using an algorithm that takes a midplane image and erodes the total nuclear area from the periphery by 20% in five consecutive steps to generate five concentric rings of equal area (Chubb et al., 2002) (Figure 1D). Although the large chromosome volume was distributed over several rings, this analysis confirmed a significant increase in the percentage of chromosome signal at the periphery with a mean of 56% in the most peripheral two rings (Figure 1E). NET39 KD not only abolished differentiation-associated repositioning, but also strongly reduced peripheral chromosome 8 in MBs indicating even basal NET39 levels undetectable by western blot (but detected by quantitative [q]RT-PCR, data not shown) can influence a chromosomes position (Figure 1E).

The repositioning activity lost by NET39 KD could be rescued by exogenously expressing human GFP-NET39, but not GFP-NLS in the NET39 KD MBs in the absence of differentiation (Figure 1E). All changes induced by altering NET39 levels were highly statistically significant using the Kolmogorov-Smirnov (KS) test (Figure S1B). Compellingly, similar chromosome 8 repositioning was observed in primary muscle cells with strong peripheral association in primary myofibers freshly isolated from mouse extensor digitorum longus (EDL) muscle paralleling C2C12 MTs, but not in primary satellite cells paralleling C2C12 MBs (Figures 1C and 1E, most right). These data clearly indicate that chromosome 8 repositions from the nuclear interior to the nuclear periphery during myogenesis and that this repositioning is largely driven by NET39.

### Global Determination of Gene Positioning Changes in Myogenesis

We next mapped global changes in genomic loci at the nuclear periphery during C2C12 myogenesis using DamID. A bacterial Dam methylase fused to lamin B1 preferentially methylated peripheral gene sequences which were isolated and identified by next generation sequencing (Vogel et al., 2007). To control for local variation in chromatin accessibility, soluble Dam methylase was expressed in parallel experiments. As MT fusion never reaches 100% in a population and both MTs and MBs were efficiently transduced, for MT preparations, MTs were isolated from remaining undifferentiated MBs by a short trypsin digestion step (Figures S2A and S2B). Genomic DNA was isolated from 3-day transduced cultures (taken at day 6 of MT differentiation), processed to enrich for Dam methylated DNA, and sequenced to yield 5.1- and 5.7-fold genome coverage in MBs and MTs, respectively.

Log2(Lamin B1 Dam/soluble Dam) ratios were then generated and used to identify lamina associated domains (LADs) in MBs and MTs as described previously (Wu and Yao, 2013). Similar to an earlier DamID study differentiating embryonic stem cells into neuronal precursors (Peric-Hupkes et al., 2010), the majority of LADs identified in MBs were retained in MTs: 94% of the LAD coverage was shared between MTs and MBs with the remaining 6% roughly equally distributed between lost and newly formed LADs (Figure 2A). Although many LADs were shared between MBs and MTs, the signal intensity from log2(Lamin B1/Dam) ratios frequently differed considerably with discrete regions displaying a quantifiably increased or decreased ratio in MBs or MTs. This indicated a binary LAD or non-LAD definition is insufficient to identify regions with altered peripheral association between MBs and MTs. Since the log2(Lamin B1/Dam) signal represents the probability of peripheral localization (Peric-Hupkes et al., 2010), we instead developed a statistical method, described in detail in the Supplemental Information, to identify regions that exhibit significant changes in lamina association in MBs and MTs. In all, a tendency to relocate from the interior in MBs to the periphery in MTs (denoted "IP") was observed for 713 regions that were often proximal to the edges of MB genome regions enriched in LADs, with an average size of 416 kb containing 2,197 genes. The opposite tendency (shifting from the periphery to the interior; "PI") was observed for 1,034 regions with an average size of 539 kb containing 2,939 genes (Figures 2A and S3A). IP and PI regions can be observed in a chromosomal context in Figure S3A ideograms and all DamID identified genes are listed in Table S1.

Consistent with an earlier neuronal differentiation study (Peric-Hupkes et al., 2010), genes located within IP regions strongly tended to become repressed during differentiation instead of activated, though many were unchanged. Out of 2,197 genes in IP regions, 933 became at least 1.4-fold repressed, 991 were unchanged, and only 266 increased at least 1.4-fold in

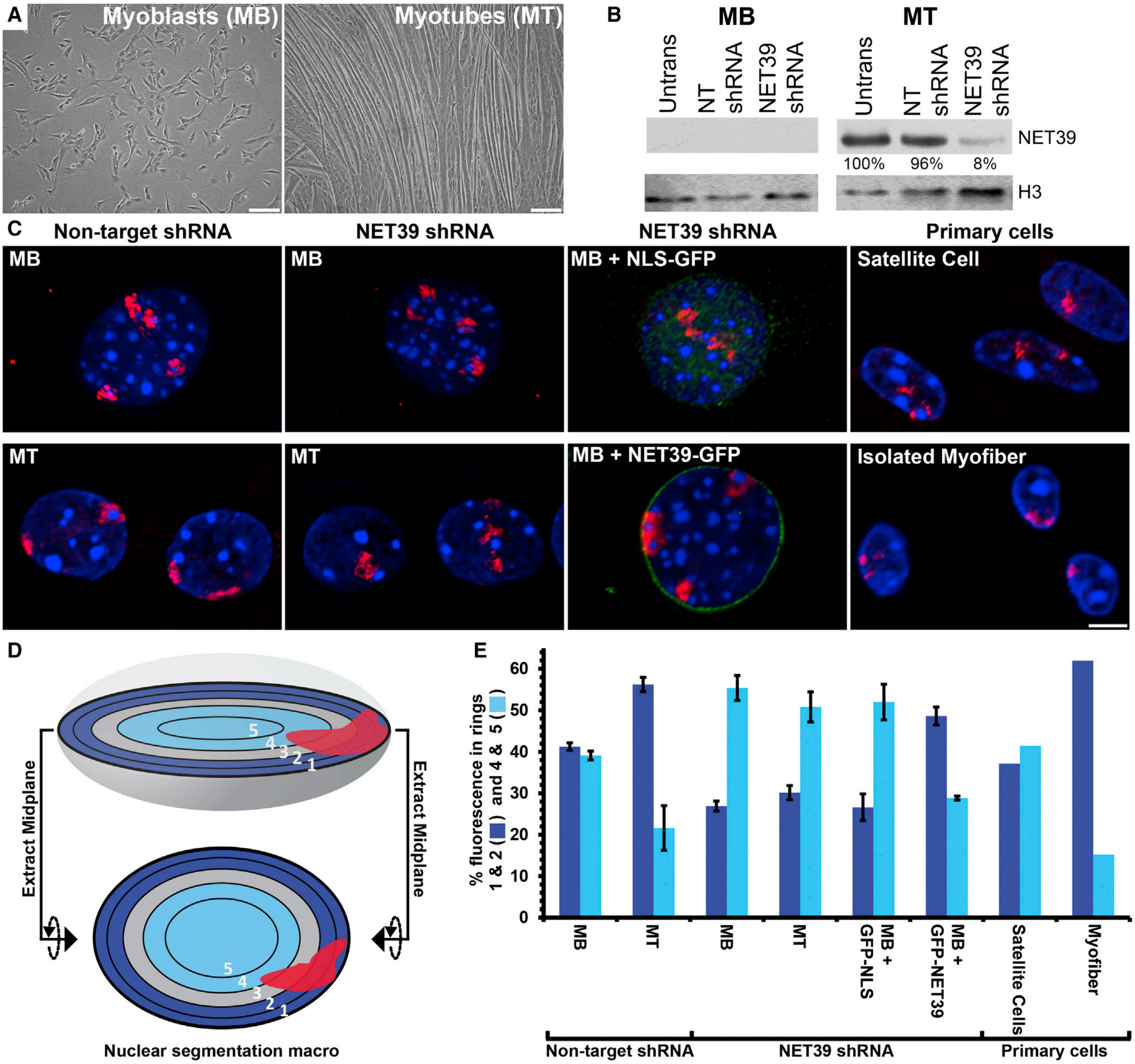


**Figure 1. NET39 Determines the Position of Chromosome 8 during Myogenesis**

(A) Micrographs showing normal C2C12 in vitro myogenic differentiation with undifferentiated MBs forming multi-nucleated MTs.
(B) Western blot confirming the depletion of NET39 in NET39-shRNA treated MTs.
(C) Representative images of the position of chromosome 8 in indicated samples. The scale bar represents 5 μm.
(D) Schematic describing analysis of chromosome position at the nuclear midplane.
(E) Quantification of chromosome 8 position in indicated samples. The error bars represent the SD of the means of two biological repeats of at least 50 nuclei each. For primary cells, single experiments were performed and so the error bars are absent.
See also Figure S1.

expression based on ENCODE C2C12 RNA-sequencing (seq) data (Yue et al., 2014). As our DamID time point was 2 days later in differentiation than available RNA-seq data, we performed expression analysis using microarrays to parallel the DamID. Consistent with similar published RNA-seq data (Figures S3B and S3C), microarray data revealed that genes in IP regions tend to be repressed, while genes in PI regions tend to be activated during differentiation. Titin, an important gene for muscle differentiation, was in this latter category (Figure 2B). Release of the Titin locus from the periphery during myogenesis was also evident from FISH, confirming both the DamID data and the statistical approach used to identify repositioning loci.

Many PI regions contained important myogenic genes that need to be activated for muscle differentiation: *Ryr3* encodes a

CellPress

A

| Sample | % of Mouse Genome | Number | % unique | Median Size (Kbp) | Average Size (Kbp) | Min. Size (Kbp) | Max. Size (Mbp) | # Genes |
|---|---|---|---|---|---|---|---|---|
| Myoblasts LADs | 28.8% | 22,890 | 3% | 18 | 33.4 | 2 | 1.0 | 7226 (36%) |
| Myotube LADs | 30.1% | 24,765 | 3% | 20 | 32.3 | 2 | 0.9 | 6525 (32%) |
| IP Regions | 11.2% | 713 | N/A | 285 | 416 | 53 | 3.70 | 2197 (11%) |
| PI Regions | 21.0% | 1034 | | 386 | 539 | 58 | 3.90 | 2939 (15%) |

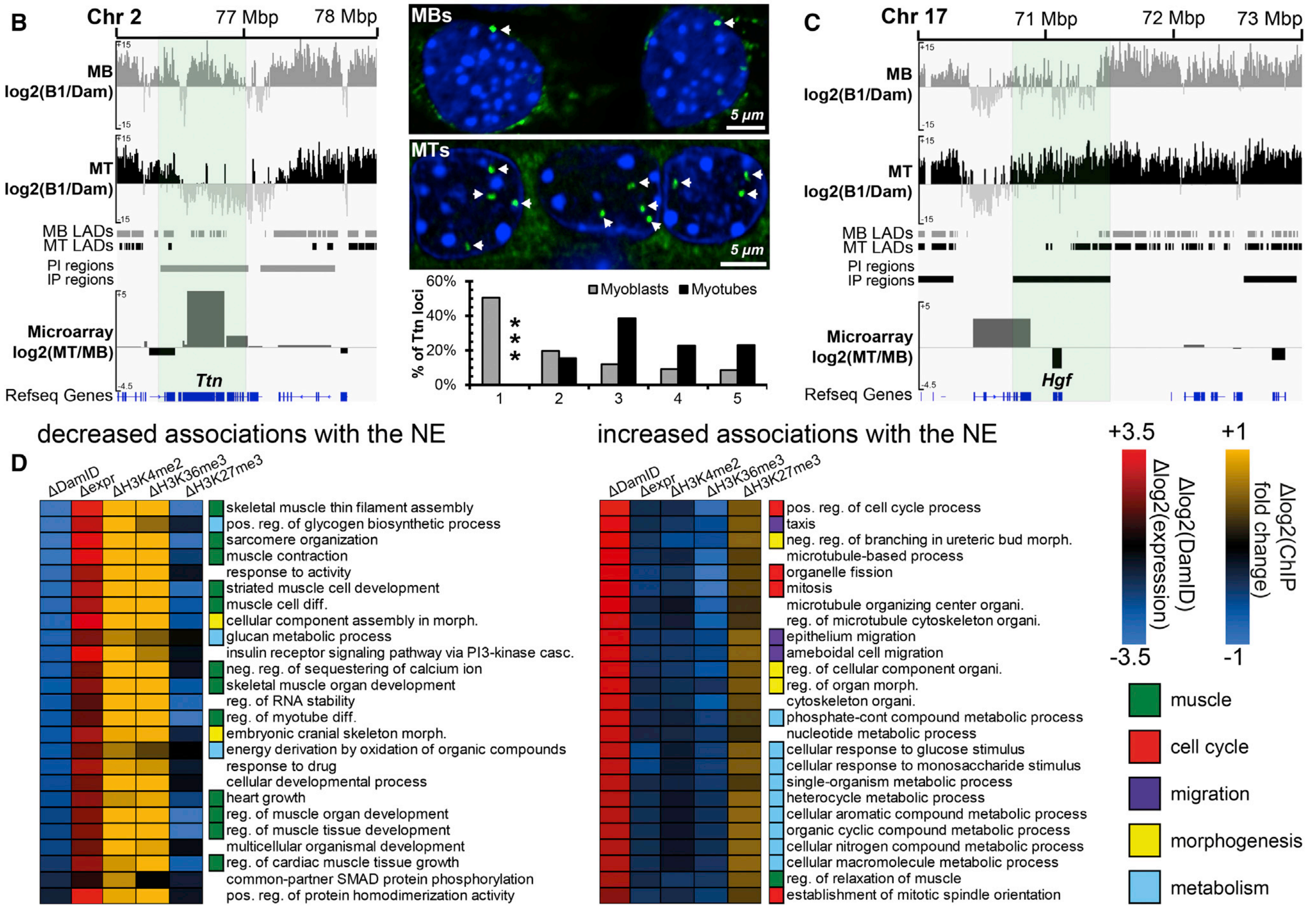


**Figure 2. Global Identification of Loci Repositioning in C2C12 Myogenesis**

(A) Table summarizing parameters of identified LADs and regions showing increased (IP) and decreased (PI) association with the nuclear periphery.

(B) Example genome browser view of the *Ttn* locus with representative images and direct quantification of the position of *Ttn* loci in 100 nuclei (green) by FISH in MBs and MTs. ***$p < 0.001$ comparing locus position in MTs to MBs using $\chi^2$ test.

(C) Genome browser view for the genomic region surrounding *Hgf* showing DamID signal intensities, identified LADs, IP and PI regions, and microarray gene expression changes for MBs and MTs.

(D) Heatmap displaying average Δlog2 DamID, Δlog2 expression, and Δlog(fold change) for indicated histone modifications values between MTs and MBs for genes within GO term categories significantly enriched in PI activated genes and IP repressed genes. Myogenic alterations to histone modifications associated with transcriptionally active genes (H3K4me2 and H3K36me3) and the transcriptional repression-associated H3K27me3 were extracted from Asp et al. (2011). The Δlog2(DamID) value was calculated by subtracting the average log2(Lamin B1/Dam) value in a 100 kb window surrounding the gene in the MB sample from the MT sample. The ChIP-seq values were determined for each histone modification by subtracting the average signal across the gene body in the MB sample from the MT sample.

See also Figures S2 and S3.

ryanodine receptor important for $Ca^{2+}$ regulation, *Actc1* for muscle actin, and *Myo18b* for a muscle myosin (Figure S2C). Correspondingly, IP regions contained genes inhibitory to myogenesis that need to be repressed in MTs. For example, IP gene *Hgf* encodes hepatic growth factor that, although required for the activity of satellite cells, is inhibitory to myogenesis when present during differentiation (Figure 2C) (Dietrich et al., 1999; Yamada et al., 2010). Similarly, *Dbf4* and *Cdk14* products promote cell-cycle progression (Davidson et al., 2009; Kumagai et al., 1999) that, while required in cycling satellite cells, is detrimental in terminally differentiated MTs (Figure S2C). To globally associate repositioning with gene function, analysis of Gene Ontology (GO) term enrichment of repressed IP and activated PI genes was performed. The top 25 GO-terms for each are shown compared to a

heatmap for their expression changes in the matched myogenic microarray samples and available chromatin immunoprecipitation (ChIP)-seq data for myogenesis (Asp et al., 2011) (Figure 2D). The expression changes for these PI region genes correlated with the loss of the repressive H3K27me3 mark and accumulation of active histone marks such as H3K4me2, H3K4me3, H3K9Ac, and H3K36me3 (Figure S2D). They also strongly associated with muscle functions and differentiation. In contrast, these IP genes correlated with loss of active histone marks and gain of H3K27me3, reflecting silencing of genes functioning in the cell cycle, cell migration, morphogenesis, and altered metabolism. There were no clear associations with GO-categories relating to myogenesis for IP and PI genes that were oppositely regulated in expression (Figures S3D–S3G). Hence, repositioning to and from the nuclear periphery during myogenesis is generally associated with repression of genes inhibitory to myogenesis and the activation of genes required for MTs, respectively. Processed DamID and microarray data used in Figures 2, 3, and 4 can be found in Table S1.

## Together, Multiple Muscle NETs Reposition Large Chromosomal Regions

Two liver-specific NETs functioned together to position chromosome 5 in human liver cells (Zuleger et al., 2013). Therefore, it seemed likely that additional muscle NETs might contribute to the global gene positioning changes between MBs and MTs revealed by DamID. Thus, NETs found in muscle NEs (Wilkie et al., 2011) were screened using the same strategy as described in Zuleger et al. (2013) (overexpression-induced repositioning of chromosome 5 in human fibroblasts). Tmem38a, WFS1, and Tmem214 were identified as additional candidates (Figure S4A). To test if these NETs can cooperate with NET39, WFS1 was fused to GFP and Tmem38a and Tmem214 were fused to RFP. The NETs were then expressed either alone or in combination. Only cells with both RFP and GFP were analyzed to ensure that at least two NETs were expressed in the combination sample. As expected, each muscle NET by itself significantly increased peripheral localization of chromosome 5 from ~30% basal levels to ~50% (Figure 3A). Strikingly, when all four muscle NETs were co-transfected, peripheral localization increased to over 70% and was significantly higher compared to any NET alone, indicating the repositioning activity of NETs is additive (Figure 3A).

These four proteins were next confirmed as muscle-specific NETs. By western blot, NET39 and Tmem38a proteins were absent from dividing MBs (−24 hr), but were weakly detectable in the confluent cultures required for induction of differentiation (0 hr). They were then strongly expressed by 48 hr after induction (Figure 3B), well before standard myogenic markers such as the myosin heavy chain (Myh1) are expressed. WFS1 and Tmem214 were already present in MBs, and WFS1 increased during differentiation while Tmem214 did not (Figure 3B). Hence, different subsets of muscle NETs capable of repositioning chromosomes are present as myogenesis progresses and so could contribute differently to genome organization. Tissue transcriptome/proteome (Uhlén et al., 2015) analysis of NET expression revealed NET39 and Tmem38a almost exclusively in heart and muscle, while WFS1 and Tmem214 were more widely expressed (Figure S4B). WFS1 and Tmem214 were previously reported to be in the ER (Kohara et al., 2014; Li et al., 2013; Takeda et al., 2001); so their endogenous distribution was investigated with antibodies in human gastrocnemius (calf muscle), primary mouse in vitro differentiated MTs, mouse liver, and brain sections. This revealed clear NE staining exclusively in muscle, but not other cell types (Figure 3C). Thus, even though these two proteins are widely expressed, they are NETs, at least among the tissues tested, only in muscle. NET39 (not tested here) was reported to specifically target to the NE in muscle (Liu et al., 2009). Hence, all four proteins are muscle-specific NETs and would only contribute their gene/chromosome-repositioning activity from the NE in muscle.

## Global Analysis of NET-Directed Gene Positioning and Expression

To identify genomic loci specifically recruited by the muscle-specific NETs, the generation of DamID maps from NET-Dam methylase fusions was attempted. However, these were unsuccessful, possibly because the methodology requires frequent methylated GATC sites within 2 kb of one another, which might be too short for the more specific interactions expected to be involved in tissue-specific gene repositioning. However, as genes relocating to or from the periphery tended to change expression, we reasoned that depletion of gene repositioning NETs would diminish gene expression changes in the MTs, thus revealing their genomic repositioning targets. Therefore, stable shRNA KD C2C12 lines for each NET were generated, differentiated into MTs, and subjected to gene expression analysis (Figure 3D).

Largely distinct sets of genes changed expression in KD lines for NET39, Tmem38a, and WFS1 (Figure 3E). Tmem214 KD (data not shown) exhibited considerable overlap with the other NETs, suggesting it affected gene repositioning through an indirect mechanism and therefore it was not considered further. Each NET affected expression of 15%–20% of all genes that changed expression in wild-type myogenesis (Figure 4A). When considered together, NET39, WSF1, and Tmem38a affected 37% of all genes that normally change in myogenesis. Conversely, ~70% of genes altered by any individual NET changed expression during wild-type myogenesis (Figure 4B). Hence, NET depletion disproportionally affects the expression of distinct subsets of myogenic genes. Moreover, the changes induced by NET depletion were distinct from those previously reported in a MT-only KD of the transmembrane nucleoporin gp210 that had a profound effect on myogenesis (Figure S5) (D'Angelo et al., 2012). Postulating that gene expression defects resulted from a loss of NET-dependent repositioning in KD MTs, myogenic changes in gene expression and log2(Lamin B1/Dam) signal intensities were contrasted for genes within IP and PI regions (Figure 4C). This revealed a striking correlation in the directionality of changes in gene position and expression. Considering all genes changing significantly in both data sets, ~70% had changes in the expected direction, i.e., IP correlating with repression and PI with activation ($\chi^2$ p = $1.6 \times 10^{-22}$). Notably, the expression changes tended to be comparatively weak for the ~30% of genes with repositioning in the unexpected opposite direction (KS test p = $2 \times 10^{-4}$).

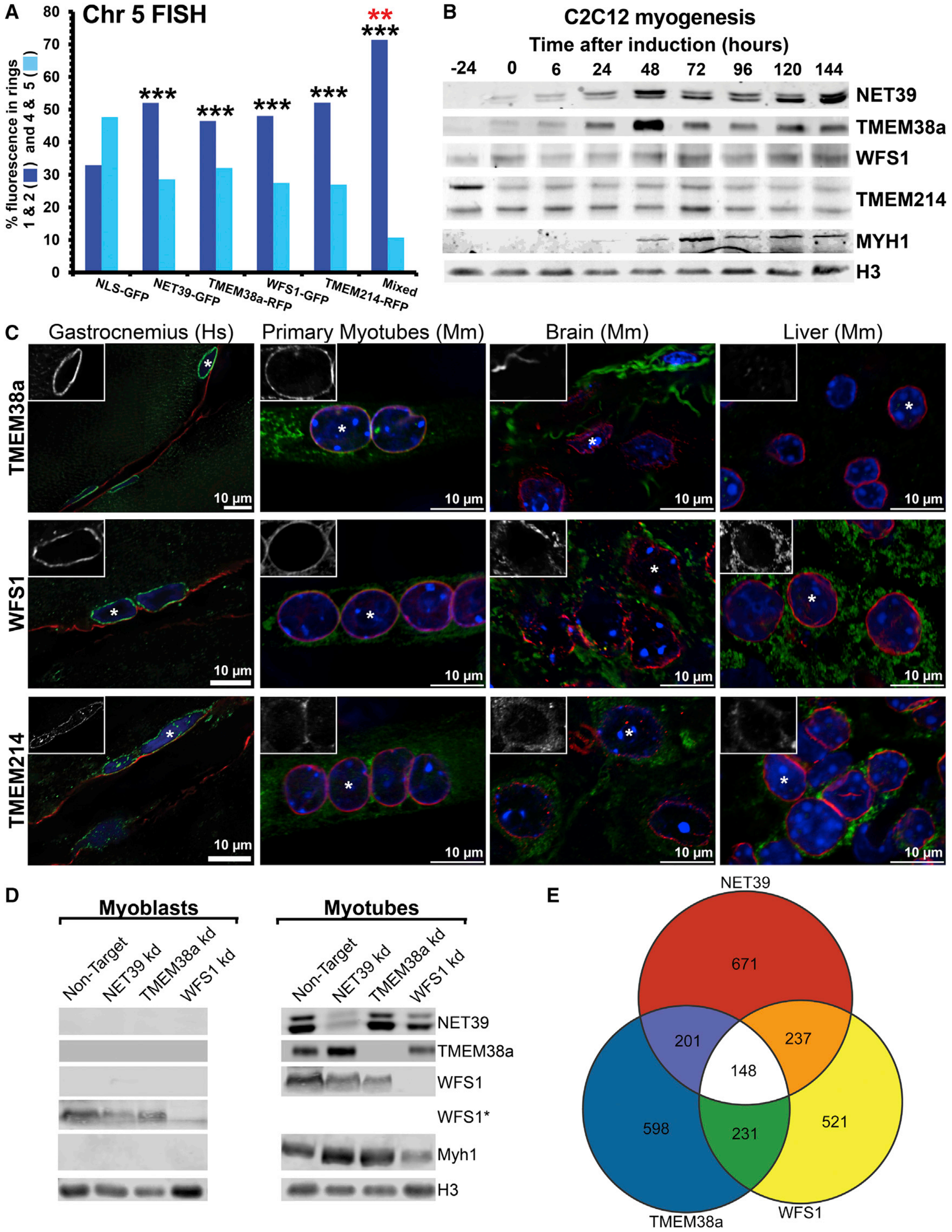


**Figure 3. Muscle-Specific NETs Function Together to Reposition a Chromosome and Genes Affected by their KD**

(A) Quantification of the position of human chromosome 5 in at least 50 HT1080 fibroblast nuclei expressing a single or multiple (Mixed) NETs. ***$p < 0.001$ comparing the position of chromosome 5 in the GFP-NET expressing cells to the NLS-GFP (black), and **$p < 0.01$ comparing mixed to individual NET expressing cells using KS tests (red).

(B) Western blot time course of C2C12 differentiation for indicated antibodies.

(C) Immunofluorescence staining of NETs (green), lamin A or B1 (red), and DNA (blue) in indicated tissue cryosections and fixed MTs generated in vitro from mouse EDL muscle-derived satellite cells. To identify myofibers in the gastrocnemius muscle section, dystrophin (red) was stained in lieu of lamins A and B1. The insert boxes show channels for NET staining individually for indicated nuclei (*).

*(legend continued on next page)*

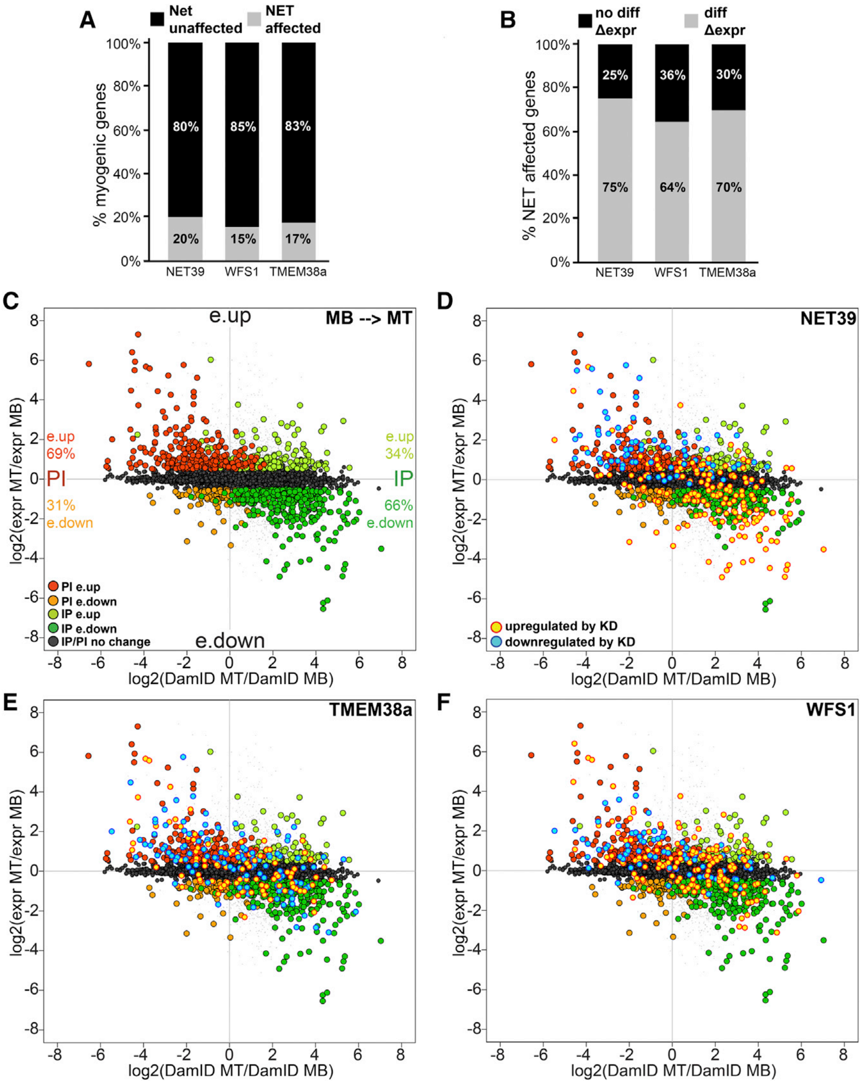


**Figure 4. Global Correlations between Gene Positioning and Expression Directed by Muscle-Specific NETs**

(A) Fraction of total genes normally changing during myogenesis that are altered by NET depletion.

(B) Fraction of total genes altered by NET depletion that normally change during myogenesis.

(C) log2(expr MT/expr MB) myogenic gene expression changes inversely correlate with average log2(DamID MT/DamID MB) myogenic lamin B1 DamID signal intensities changes. Of IP genes with altered gene expression, 66% are repressed (e.down) and 34% are activated (e.up), while for PI genes, only 31% are repressed and 69% are activated.

(D–F) Identical plots as (A) with genes highlighted if upregulated (yellow) or downregulated (blue) in indicated NET-depleted MTs relative to control MTs. The NET affected genes tend to anti-correlate, i.e., loss of the NET reduces a normal repression that occurs in myogenesis or reduces a normal increase in expression. All gene expression values are mean average changes of microarray triplicate samples.

See also Figure 5.

The genes that were altered in expression by NET KD were next overlaid onto these plots (Figures 4D–4F). 88% of gene expression effects induced by NET39 depletion were in a direction that countered normal myogenic changes. Genes normally repressed in MTs were upregulated with NET39 KD (yellow), while those normally induced were downregulated with NET39 KD (blue) (Figure 4D). Similar behavior was observed in ~60% of myogenic IP and PI genes affected by WFS1 and Tmem38a KDs (Figures 4E and 4F).

## Muscle NETs Direct Myogenic Gene Repositioning and Repression

Eight candidate genes from the intersect of the NET KD microarray and lamin B1 DamID data sets were chosen for further direct testing of NET-dependent peripheral gene repositioning by FISH. The *Nid1* gene is normally repressed early during myogenic differentiation, and the DamID data associated it with an IP region (Figure 5A). According to the microarray data, this repression of *Nid1* in MTs was strongly reduced in NET39 KD MTs (Figure 5C). FISH confirmed that *Nid1* is internal in MBs, peripheral in MTs, and fails to reposition to the periphery in NET39 KD MTs (Figure 5B). According to the microarray data, in the NET39 KD only ~60% of the normal *Nid1* repression was achieved. This difference was significant (false discovery rate < 0.001), whereas *Nid1* repression was unaffected in WFS1 and Tmem38a KDs (Figure 5C). Critically, when NET39 was exogenously expressed in MBs, *Nid1* moved to the periphery (Figure 5B), clearly showing that NET39 is sufficient to direct *Nid1* repositioning even in the absence of myogenesis. However, this ectopically induced repositioning in MBs in the absence of differentiation was not associated with gene expression changes (Figure S6B), indicating that gene repositioning requires other aspects of the cellular milieu such as transcriptional repressors induced during differentiation for its effects on gene regulation.

The *Cxcl1* locus paralleled *Nid1*, except that it was specifically regulated by WFS1 (Figures 5D–5F). FISH for *Cxcl1* similarly confirmed the DamID results and determined that the gene

(D) Western blot of pre- and post-differentiated control and NET-KD cell lines for indicated proteins.

(E) Venn diagram of total gene expression changes exceeding log2 0.5 in absolute value between NET-KD MTs relative to empty-vector treated control MTs detected by microarray analysis performed in triplicate.

See also Figure S4.

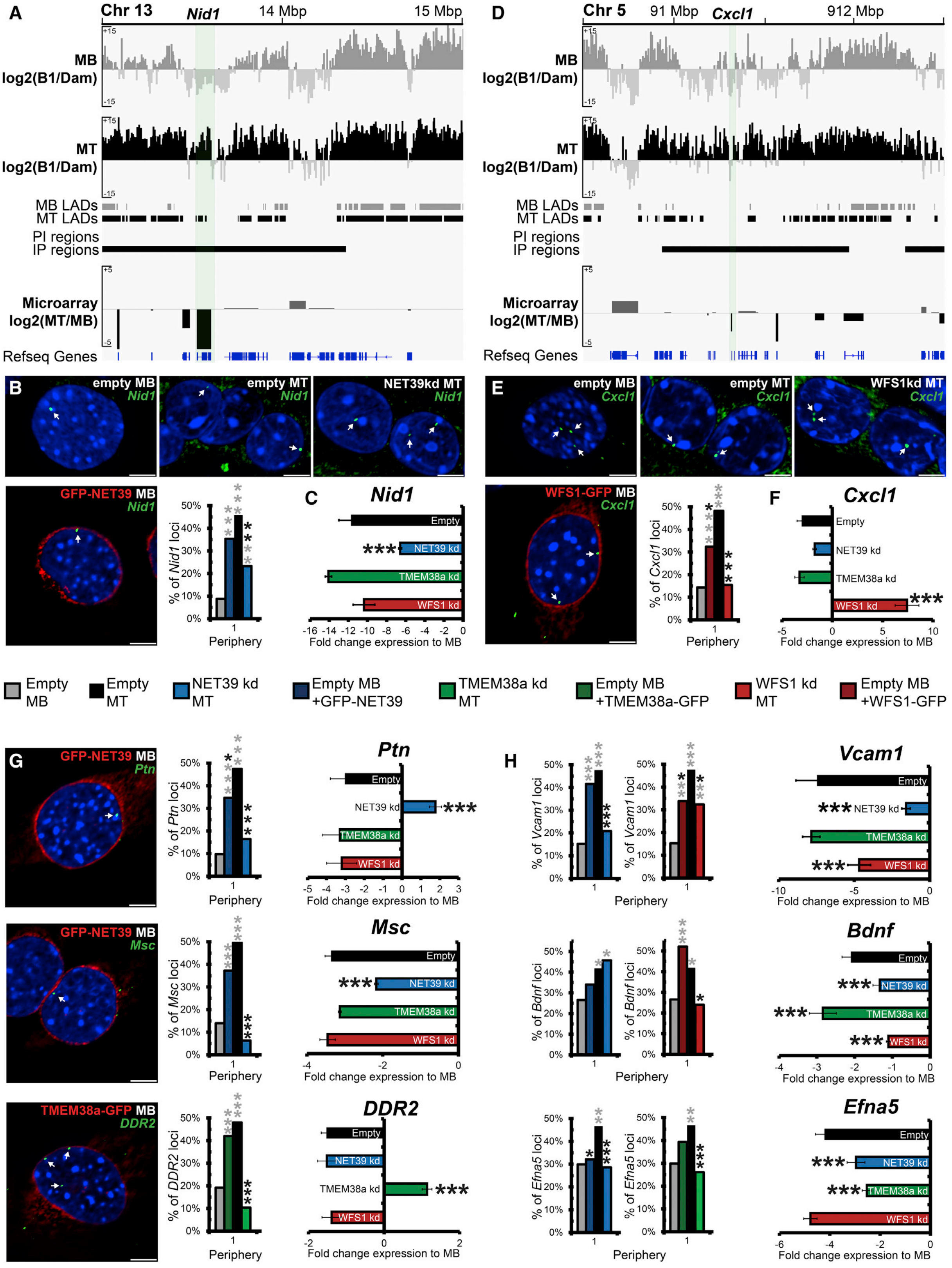


(legend on next page)

repositioning depends specifically upon WFS1. Interestingly, WFS1 KD not only abolished MT-associated downregulation of *Cxcl1*, but also led to strong upregulation compared to MBs. Similar effects were observed for the *Ptn*, *Msc*, and *DDR2* loci that were dependent upon NET39 or Tmem38a for myogenesis-associated repositioning and repression (Figure 5G).

For *Vcam1*, *Bdnf*, and *Efna5*, more than one NET affected their expression (Figure 5H). Repression of *Vcam1* reached only ~60% of normal levels with WFS1 KD and ~30% with NET39 KD. The degree of diminished repression strongly correlated with the extent of loss of peripheral association in MTs, with WFS1 KD only partially impairing and NET39 KD effectively abolishing *Vcam1* repositioning. Both NET39 and WFS1 overexpression in MBs increased *Vcam1* peripheral positioning. A similar correlation for effect strength was observed for *Bdnf* and *Efna5*, except that NET39 had a stronger effect on expression than positioning of *Bdnf* and KDs were much stronger than overexpression in repositioning of *Efna5*. Statistics for the significance of these changes can be found in Figure S6A. Interestingly, *Cxcl1* and *Vcam1* do not target to the nuclear periphery in liver where, although expressed, WFS1 is exclusively ER localized (data not shown). Thus, every gene tested depended on the NET for myogenic repositioning and this correlated with changes in gene expression during myogenesis. Moreover, seven of eight genes tested could be redirected to the periphery in MBs just by overexpressing the NET in the absence of differentiation. In all cases, as for *Nid1*, inappropriate locus repositioning in MBs induced by NET overexpression did not alter gene expression (Figures S6B and S6C).

## NET39 Redirection of Locus Positioning

As the *Ptn* locus was specifically repositioned to the periphery during myogenesis by NET39, we next investigated the functional requirements for peripheral targeting. First, we tested if its N-terminal nucleoplasmic domain was sufficient to reposition the locus when expressed as a GFP-fused soluble fragment without the membrane anchor. Although this fragment alone was insufficient for repositioning, fusing it to a surrogate transmembrane domain from chicken hepatic lectin (CHL) could reposition the locus (Figure 6A). Interestingly, the GFP-fused NET39 soluble fragment could dominant-negatively compete the relocalization function of full-length NET39 tagged with the V5 epitope (Figure 6A). Similar results were also obtained with chromosome 8 repositioning as the readout (Figures S7A and S7B). This demonstrates that NET39-mediated repositioning is not due to its reported interaction with mTOR that was mapped within the soluble fragment (Liu et al., 2009). That only the NE-tethered NET39/NET39 fragment could reposition the locus strongly argues that NET39 functions as a direct tether for *Ptn* and other genomic loci.

To further test NET39 as a direct tether, the soluble fragment was redirected to a new nuclear location and cells analyzed for *Ptn* position. When the NET39 soluble nucleoplasmic fragment was fused to nucleolin, this fusion protein accumulated at the nucleolus and so did the *Ptn* locus (Figure 6B). In contrast, overexpression of nucleolin fused to GFP had no effect on *Ptn* localization. The number and area occupied by GFP-labeled nucleolar foci was unchanged between GFP-Nucleolin and NLS-39sol-GFP-Nucleolin, indicating increased *Ptn* association was not generally due to creation of larger or more numerous nucleoli (Figures S7C and S7D). Taken together, these data strongly indicate that NET39 functions as a tether that directly binds and recruits genomic loci to the nuclear periphery, where in context of differentiation they are subject to repression.

## Gene Repositioning Muscle NETs Are Critical for Myogenesis

Corroborating the key role of these gene-repositioning NETs in controlling myogenic gene expression, defects in MT formation were observed when NET39, Tmem38a, and WFS1 were knocked down individually and in combination prior to differentiation. MTs formed for all individual KDs, though they tended to be aberrantly thick and less ordered in the Tmem38a KD. For both NET39 and TMEM38a KD, a reduced myogenic index was observed as determined by the fraction of all nuclei within Myh1 stained MTs (Figure 7A). In contrast, MT formation was almost completely abolished in the triple KD, with the few remaining MTs being extremely thick, aberrantly shaped, and disordered. Phase contrast live cell imaging also revealed the presence of large vacuoles in Tmem38a- and WFS1-depleted MTs (Figure S7). Moreover, the kinetics of MT formation were reduced upon NET39 and TMEM38a KD and severely diminished in the triple KD (Movie S1). Hence, the loss of these three NETs, both individually and collectively, has profound consequences for myogenesis and the physical characteristics of any MTs formed.

## DISCUSSION

The combination of DamID, transcriptomics, and FISH used here has clearly shown the existence of a mechanism for repositioning specific developmental genes by tissue-specific NETs. This mechanism is distinct from the previously described gene positioning due to recruitment of silenced chromatin to the periphery (Peric-Hupkes et al., 2010; Solovei et al., 2013).

**Figure 5. FISH and Microarray Analysis in NET KDs Confirms NET-Directed Repositioning and Expression Regulation**

(A and D) Genome browser views of myogenic DamID and gene expression changes of the *Nid1* (A) and *Cxcl1* loci (D).

(B and E) FISH analysis of *Nid1* (B) and *Cxcl1* (E) loci position in control MBs and MTs, NET-depleted MTs, and NET-overexpressing MBs.

(C and F) Histogram of *Nid1* (C) and *Cxcl1* (F) gene expression changes relative to MBs in control and NET-depleted MTs.

(G and H) Similar microarray and FISH analysis of genes affected uniquely by depletion of a NET (*Ptn*, *Msc*, and *DDR2*) (G) and by depletion of multiple NETs (*Vcam1*, *Bdnf*, and *Efna5*) (H). For microarray data, error bars represent SD over three biological repeats, while statistics represent false discovery rates (FDR) between sample and an empty vector-treated control. For FISH, loci position was determined in 50–100 nuclei for each sample. For quantification statistics, the position of loci in the indicated sample was compared to the empty-vector MBs (gray asterisks) or MTs (black asterisks) using $\chi^2$ tests. *p < 0.05, **p < 0.01, and ***p < 0.001.

All FISH statistics are in Figure S6. The scale bars represent 5 μm.

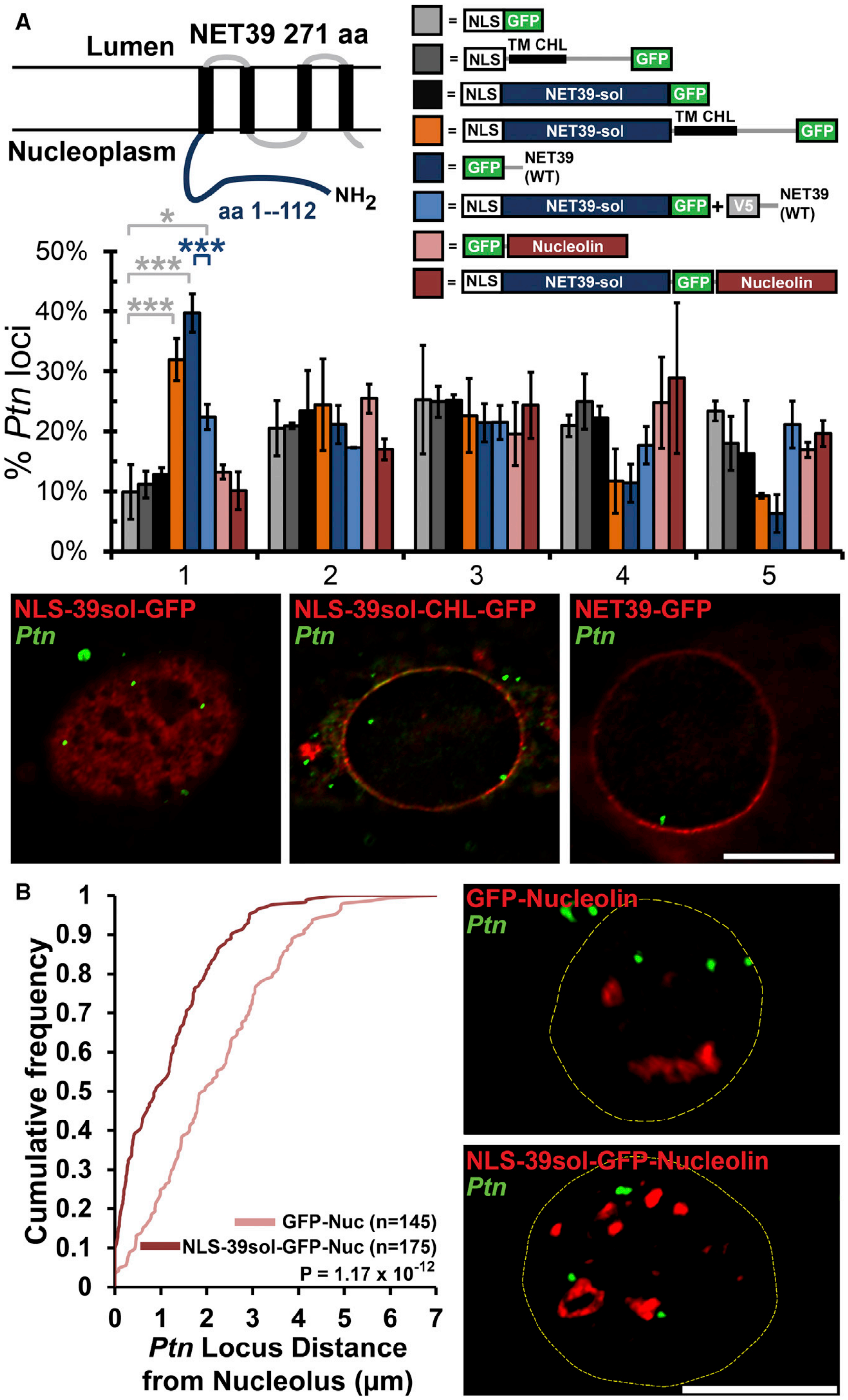


**Figure 6. Direct Tethering Function of NET39**

(A) Schematic diagrams of NET39 and fusion constructs with FISH analysis and representative images of *Ptn* loci positioning in indicated samples. The error bars represent SD of the mean over two biological repeats. ***p < 0.001 and *p < 0.05 comparing samples to NLS-GFP expressing MBs by $\chi^2$ tests.

(B) Representative images and cumulative frequency distribution of *Ptn* loci distance from the edge of nucleoli in indicated samples expressing either the NET39 soluble fragment fused to nucleolin or nucleolin-GFP over two summed biological repeats. For all images, *Ptn* is labeled green and GFP is labeled red.

The scale bars represent 5 µm. See also Figure S7.

## NET Targets Are Genes Requiring Fine-Tuned Regulation

We argue that this new type of gene positioning is focused on genes that need to be tightly regulated, often at critical times during differentiation and in a tissue-specific manner. Among those shown here, Musculin, encoded by *Msc*, occludes myoD binding sites to inhibit myogenesis and so must be very tightly repressed for myogenesis to progress (Lu et al., 1999). Nidogen 1, encoded by *Nid1*, is secreted into the extracellular matrix of early developing MTs. While important at this early time window, *Nid1* becomes repressed early in myogenesis and expressing Nidogen 1 after this point inhibits further differentiation (Neu et al., 2006). Vcam1, encoded by *Vcam1*, and its counter receptor Vla4 mediate interactions for satellite cell migration and MB-MT fusion, but Vla4 remains on myofibers, while Vcam1 disappears (Jesse et al., 1998; Rosen et al., 1992), suggesting it also must be tightly temporally regulated. Similarly, the *Ptn*, *Hgf, Efna5*, and *Bdnf* genes are all downregulated later in myogenesis, but are needed early for respective formation of neuromuscular junctions (Caruelle et al., 2004; Peng et al., 1995), migration of precursors (Dietrich et al., 1999), alignment of fusing MBs (Stark et al., 2011), and precursor functions (Mousavi and Jasmin, 2006). Likewise, the many cell-cycle genes thus regulated are needed in MBs to generate a sufficient number of cells to fuse into MTs, but must be tightly repressed once myofibers have formed. Additionally *Efna5*, *Cxcl1*, and *Ptn* are all induced upon muscle damage (Caruelle et al., 2004; De Paepe et al., 2012; Stark et al., 2011), suggesting that the peripheral tethering of genes at the NE places them under an additional regulatory control that can respond to muscle damage in order to reverse repression.

## Categories of Gene Regulation in Myogenesis

Use of the robust C2C12 differentiation system enabled our identification of IP and PI regions regulated by muscle-specific NETs. However, this only accounts for a subset of myogenic

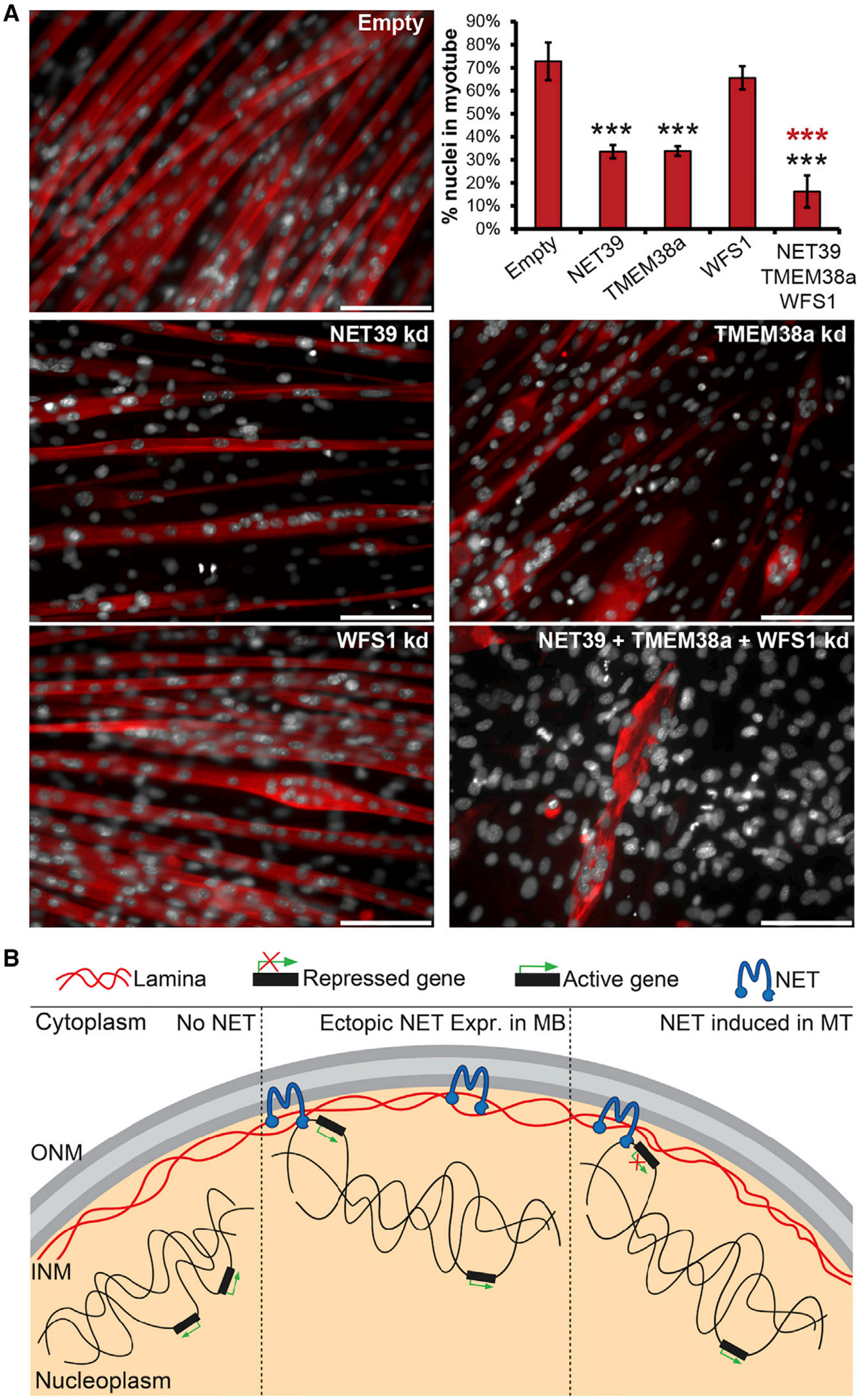


**Figure 7. Gene Repositioning Muscle NETs Are Critical for Myogenic Differentiation**

(A) Representative images and quantification of the fraction of nuclei in MTs (marked by Myh1, red) in indicated samples. ***p < 0.001 comparing the fraction of total nuclei present within MTs relative to empty vector-treated MTs by a $\chi^2$ test. The error bars represent the SD between 5–10 fields across three biological repeats. The scale bar represents 100 μm.

(B) In MBs with low levels of muscle NETs, target genes are active in the transcriptionally permissive interior. Ectopic expression of these NETs in MBs results in peripheral gene targeting without repression. However, during differentiation, normal induction of the NET repositions the locus to the periphery concomitant with an increase in repression. Loss of the gene-repositioning NET results in both failure to reposition the locus and reduced repression (Inner [INM] and outer [ONM] nuclear membranes).

See also Figure S7 and Movie S1.

gene expression changes. The remaining IP and PI genes that repositioned and changed expression may be regulated by as yet unidentified muscle NETs or previously described heterochromatin and chromatin folding-dependent NE associations (Solovei et al., 2013). Genes that changed expression without repositioning are likely purely dependent upon transcription factor cascades or secondary/propagating effects (e.g., a repositioned gene encodes a transcriptional regulator). Separate functions for these NETs have been reported, the loss of which could contribute to gene expression changes. However, a reported function of WFS1 in ER stress responses is unlikely to be relevant as WFS1 was only in the NE in muscle (Fonseca et al., 2005). A calcium transport function reported for Tmem38a in the sarcoplasmic reticulum is similarly likely distinct from its NE role (Yazawa et al., 2007). Some NET39 gene regulation effects could be attributed to signaling changes from its reported function inhibiting mTOR activity (Liu et al., 2009). However, its ability to retarget *Ptn* to nucleoli strongly supports a more direct physical tethering function.

Loss or gain of peripheral chromatin associations will also inevitably influence internal genome organization. For example NET-tethering of a locus normally in an internal topologically associating domain (TAD) (Bickmore and van Steensel, 2013) would either move the TAD to the periphery or disrupt it. Alternatively, peripheral tethering could alter the genes available for long range inter-TAD interactions or transcription factories, thereby altering the expression of associated genes (Pombo and Dillon, 2015). This may explain how a NET involved in peripheral tethering also promotes altered expression of internally localized genes. Finally, IP and PI genes that repositioned without changing expression perhaps lacked transcriptional regulators in the muscle cells and may have moved as a consequence of the directed restructuring of adjacent genes rather than for their own regulation. This suggests that the effects described here are a central part of a much larger picture where

transcriptional regulators bound to chromatin also play an important role and NET-directed positioning serves as an additional layer of regulation to further fine tune gene expression.

## Segregating Gene Positioning Effects from Other Aspects of Differentiation

The potential role of transcriptional regulators in the tethering nexus is prescient with regard to the central question of the actual contribution of peripheral positioning to gene regulation. The ability to disrupt the peripheral positioning of specific loci while other aspects of differentiation proceeded enabled distinguishing the contribution of positioning from other myogenic transcriptional regulatory cascades. Myogenesis proceeded with the KD of any individual NET and the genes were still partly repressed even when displaced from the periphery, indicating transcriptional repressors were present. Thus, this study has for the first time been able to distinguish for an endogenous locus, without artificial interventions, the relative contribution of peripheral positioning to gene repression, which generally seems to contribute between 1/3 and 2/3 of the total repression observed in normal myogenesis.

In contrast, no changes in expression were observed when NETs were exogenously expressed in MBs, indicating that repositioning to the NE is not intrinsically associated with repression. Rather, the combination of nuclear repositioning with other aspects of the cellular differentiation milieu allows optimal repression to be achieved once silencing is induced. We posit that tissue-specific NET-directed gene repositioning adds an extra layer of regulation to critical myogenic genes in order to better control their appropriate expression and repression at different myogenic stages (Figure 7B). The cumulative effect of these changes can yield repositioning of whole chromosomes. Determining NET binding partners on chromatin and the relative affinity of these and other NE-chromatin interactions will be important in the future. These NETs might also be important for NE-linked diseases. Lamins and several widely expressed NETs have been linked to Emery-Dreifuss and limb-girdle muscular dystrophies, but it is unclear how the tissue-specific muscle pathology is achieved. However, many myogenic genes are altered in these diseases (Bakay et al., 2006; Sáenz et al., 2008), suggesting the possibility that disrupted interactions with these muscle-specific NETs could underlie disease pathology. For now it is clear that these NETs direct tissue-specific patterns of gene positioning in myogenesis and contribute to the extent of gene repression from this peripheral tethering. Moreover, that 37% of all genes normally changing in myogenesis were affected by KD of three muscle-specific gene-positioning NETs, unequivocally establishes the importance of this type of regulation by tissue-specific NETs for muscle differentiation.

## EXPERIMENTAL PROCEDURES

### Cells and Transduction

C2C12 MBs were cultured under ATCC-recommended conditions and induced to differentiate at 48 hr post-confluency by addition of DMEM with 2% horse serum. Differentiation media was replaced every 48 hr up to 144 hr post-induction. To inhibit contraction, 1 μM tetrodotoxin was added at 96 hr post-induction. VSV-G pseudotyped lentivirus' encoding DamID, GFP, or pLKO constructs are described in Supplemental Information. 10 μg/ml protoamine sulfate was added during transduction to enhance efficiency. 1.5 μg/ml puromycin was used for selection of pLKO-encoding lentivirus transduced cells.

### FISH

C2C12 MBs and MTs were cultured on coverslips and fixed in 4% paraformaldehyde (PFA), 1× PBS. FISH was performed as described in Zuleger et al. (2013). Briefly, cells were permeabilized, treated with RNase A, and dehydrated with an ethanol series. DNA was denatured and captured in this state by a second ice-cold ethanol dehydration series. Coverslips were then annealed overnight to labeled BAC or whole chromosome probes. After washing, probes were visualized with Alexa Fluor-conjugated Streptavidin/anti-digoxigenin antibodies and total DNA visualized with DAPI. To identify overexpressing cells, they were stained with GFP antibodies before FISH. Coverslips were mounted in Vectashield (Vector Labs), images of nuclear midplanes acquired, and chromosome and gene positions determined using a macro run in Image-Pro Plus. This measures nuclear area from DAPI images, divides the area into five shells of equal area through eroding 20% of total area in steps from the DAPI-defined nucleus, determines the shell containing the gene spot or the chromosome intensity per shell, and sums this for each cell.

### Microarrays

Extracted total RNA from control MBs, MTs, and NET-depleted MTs was converted to biotin labeled-cRNA using the Illumina TotalPrep RNA Amplification Kit (Ambion, AMIL1791). For each analysis, three biological replicates were hybridized to MouseWD6 BeadChip Illumina whole genome expression arrays. Microarray data were quantile normalized, analyzed, and differentially expressed transcripts selected with a log2 ratio above 0.5 in absolute value using moderated F-statistics adjusted for a false discovery rate of 5%.

### DamID

DamID was performed as in Vogel et al. (2007). Briefly, C2C12 MBs and 72 hr differentiated C2C12 MTs were both separately transduced with Dam-Lamin B1- and Dam only-encoding lentiviruses over 24 hr. At 72 hr post-transduction, genomic DNA was extracted from trypsinized MBs and purified MTs and processed into libraries for next generation sequencing (Beijing Genomics). Sequenced reads were mapped to the mouse MM9 genome and the log2(Lamin B1/Dam) value determined for all genomic *DpnI* fragments in MBs and MTs. IP and PI regions were then identified by comparing the mean intensity differences between MT and MB log2(Lamin B1/Dam) values along a running 100 kb window. Regions with a mean MT/MB lamin B1 signal difference of 2-fold were then tested for significance against a randomized signal sample population using Fisher's exact test over 1,000 iterations. Regions with $p > 0.01$ were disregarded.

### Immunofluorescence

The human muscle section from a control donor was obtained with informed consent of the donor and provided by Benedikt Schoser (Ludwig-Maximilians-Universität, München) through the Muscle Tissue Culture Collection at the Friedrich-Baur-Institüt. This collection is part of MD-NET and funded by the German Ministry of Education and Research and is partnered with EuroBioBank and TREAT-NMD. Local ethics approval was also obtained for use of human tissue from the University of Edinburgh School of Health in Social Science Research Ethics Panel. Mouse tissues were obtained in accordance with both University of Edinburgh and UK Home Office ethics approval and under Home Office License PPL 70/8175 to E.C.S.

## ACCESSION NUMBERS

The accession number for the MB and MT DamID and gene expression data reported in this paper is GEO: GSE80330.

## SUPPLEMENTAL INFORMATION

Supplemental Information includes Supplemental Experimental Procedures, seven figures, one table, and one movie and can be found with this article online at http://dx.doi.org/10.1016/j.molcel.2016.04.035.

## AUTHOR CONTRIBUTIONS

Most experimental work, M.I.R.; EDL muscle preparation, R.C.; Bioinformatics, J.I.d.I.H., S.W., and A.R.W.K.; Tissue staining, P.L.T.; EM, D.G.B.; and Project design and writing, M.I.R. and E.C.S.

## ACKNOWLEDGMENTS

We thank Louise Evenden at the Wellcome Trust Clinical Research Facility for microarray analysis; A.J. Beckett and I.A. Prior at the Biomedical Electron Microscopy Unit, University of Liverpool for 3view EM; Benedikt Schoser for human tissue sections; and Andrea Rizzotto for schematic design. M.I.R. and P.L.T. were funded by Wellcome Trust and MRC Studentships. This work was funded by Wellcome Trust grants 095209 to E.C.S. and 092076 for the Centre for Cell Biology.

Received: September 22, 2015
Revised: December 21, 2015
Accepted: April 28, 2016
Published: June 2, 2016

## REFERENCES

Akhtar, W., de Jong, J., Pindyurin, A.V., Pagie, L., Meuleman, W., de Ridder, J., Berns, A., Wessels, L.F., van Lohuizen, M., and van Steensel, B. (2013). Chromatin position effects assayed by thousands of reporters integrated in parallel. Cell *154*, 914–927.

Asp, P., Blum, R., Vethantham, V., Parisi, F., Micsinai, M., Cheng, J., Bowman, C., Kluger, Y., and Dynlacht, B.D. (2011). Genome-wide remodeling of the epigenetic landscape during myogenic differentiation. Proc. Natl. Acad. Sci. USA *108*, E149–E158.

Bakay, M., Wang, Z., Melcon, G., Schiltz, L., Xuan, J., Zhao, P., Sartorelli, V., Seo, J., Pegoraro, E., Angelini, C., et al. (2006). Nuclear envelope dystrophies show a transcriptional fingerprint suggesting disruption of Rb-MyoD pathways in muscle regeneration. Brain *129*, 996–1013.

Bickmore, W.A., and van Steensel, B. (2013). Genome architecture: domain organization of interphase chromosomes. Cell *152*, 1270–1284.

Caruelle, D., Mazouzi, Z., Husmann, I., Delbé, J., Duchesnay, A., Gautron, J., Martelly, I., and Courty, J. (2004). Upregulation of HARP during in vitro myogenesis and rat soleus muscle regeneration. J. Muscle Res. Cell Motil. *25*, 45–53.

Chubb, J.R., Boyle, S., Perry, P., and Bickmore, W.A. (2002). Chromatin motion is constrained by association with nuclear compartments in human cells. Curr. Biol. *12*, 439–445.

D'Angelo, M.A., Gomez-Cavazos, J.S., Mei, A., Lackner, D.H., and Hetzer, M.W. (2012). A change in nuclear pore complex composition regulates cell differentiation. Dev. Cell *22*, 446–458.

Davidson, G., Shen, J., Huang, Y.L., Su, Y., Karaulanov, E., Bartscherer, K., Hassler, C., Stannek, P., Boutros, M., and Niehrs, C. (2009). Cell cycle control of wnt receptor activation. Dev. Cell *17*, 788–799.

De Paepe, B., Creus, K.K., Martin, J.J., and De Bleecker, J.L. (2012). Upregulation of chemokines and their receptors in Duchenne muscular dystrophy: potential for attenuation of myofiber necrosis. Muscle Nerve *46*, 917–925.

Dietrich, S., Abou-Rebyeh, F., Brohmann, H., Bladt, F., Sonnenberg-Riethmacher, E., Yamaai, T., Lumsden, A., Brand-Saberi, B., and Birchmeier, C. (1999). The role of SF/HGF and c-Met in the development of skeletal muscle. Development *126*, 1621–1629.

Finlan, L.E., Sproul, D., Thomson, I., Boyle, S., Kerr, E., Perry, P., Ylstra, B., Chubb, J.R., and Bickmore, W.A. (2008). Recruitment to the nuclear periphery can alter expression of genes in human cells. PLoS Genet. *4*, e1000039.

Fonseca, S.G., Fukuma, M., Lipson, K.L., Nguyen, L.X., Allen, J.R., Oka, Y., and Urano, F. (2005). WFS1 is a novel component of the unfolded protein response and maintains homeostasis of the endoplasmic reticulum in pancreatic beta-cells. J. Biol. Chem. *280*, 39609–39615.

Jesse, T.L., LaChance, R., Iademarco, M.F., and Dean, D.C. (1998). Interferon regulatory factor-2 is a transcriptional activator in muscle where It regulates expression of vascular cell adhesion molecule-1. J. Cell Biol. *140*, 1265–1276.

Kohara, K., Pignatelli, M., Rivest, A.J., Jung, H.Y., Kitamura, T., Suh, J., Frank, D., Kajikawa, K., Mise, N., Obata, Y., et al. (2014). Cell type-specific genetic and optogenetic tools reveal hippocampal CA2 circuits. Nat. Neurosci. *17*, 269–279.

Kumagai, H., Sato, N., Yamada, M., Mahony, D., Seghezzi, W., Lees, E., Arai, K., and Masai, H. (1999). A novel growth- and cell cycle-regulated protein, ASK, activates human Cdc7-related kinase and is essential for G1/S transition in mammalian cells. Mol. Cell. Biol. *19*, 5083–5095.

Kumaran, R.I., and Spector, D.L. (2008). A genetic locus targeted to the nuclear periphery in living cells maintains its transcriptional competence. J. Cell Biol. *180*, 51–65.

Li, C., Wei, J., Li, Y., He, X., Zhou, Q., Yan, J., Zhang, J., Liu, Y., Liu, Y., and Shu, H.B. (2013). Transmembrane protein 214 (TMEM214) mediates endoplasmic reticulum stress-induced caspase 4 enzyme activation and apoptosis. J. Biol. Chem. *288*, 17908–17917.

Liu, G.H., Guan, T., Datta, K., Coppinger, J., Yates, J., 3rd, and Gerace, L. (2009). Regulation of myoblast differentiation by the nuclear envelope protein NET39. Mol. Cell. Biol. *29*, 5800–5812.

Lu, J., Webb, R., Richardson, J.A., and Olson, E.N. (1999). MyoR: a muscle-restricted basic helix-loop-helix transcription factor that antagonizes the actions of MyoD. Proc. Natl. Acad. Sci. USA *96*, 552–557.

Mattout, A., Pike, B.L., Towbin, B.D., Bank, E.M., Gonzalez-Sandoval, A., Stadler, M.B., Meister, P., Gruenbaum, Y., and Gasser, S.M. (2011). An EDMD mutation in C. elegans lamin blocks muscle-specific gene relocation and compromises muscle integrity. Curr. Biol. *21*, 1603–1614.

Mousavi, K., and Jasmin, B.J. (2006). BDNF is expressed in skeletal muscle satellite cells and inhibits myogenic differentiation. J. Neurosci. *26*, 5739–5749.

Neu, R., Adams, S., and Munz, B. (2006). Differential expression of entactin-1/nidogen-1 and entactin-2/nidogen-2 in myogenic differentiation. Differentiation *74*, 573–582.

Peng, H.B., Ali, A.A., Dai, Z., Daggett, D.F., Raulo, E., and Rauvala, H. (1995). The role of heparin-binding growth-associated molecule (HB-GAM) in the postsynaptic induction in cultured muscle cells. J. Neurosci. *15*, 3027–3038.

Peric-Hupkes, D., Meuleman, W., Pagie, L., Bruggeman, S.W., Solovei, I., Brugman, W., Gräf, S., Flicek, P., Kerkhoven, R.M., van Lohuizen, M., et al. (2010). Molecular maps of the reorganization of genome-nuclear lamina interactions during differentiation. Mol. Cell *38*, 603–613.

Pombo, A., and Dillon, N. (2015). Three-dimensional genome architecture: players and mechanisms. Nat. Rev. Mol. Cell Biol. *16*, 245–257.

Reddy, K.L., Zullo, J.M., Bertolino, E., and Singh, H. (2008). Transcriptional repression mediated by repositioning of genes to the nuclear lamina. Nature *452*, 243–247.

Rosen, G.D., Sanes, J.R., LaChance, R., Cunningham, J.M., Roman, J., and Dean, D.C. (1992). Roles for the integrin VLA-4 and its counter receptor VCAM-1 in myogenesis. Cell *69*, 1107–1119.

Sáenz, A., Azpitarte, M., Armañanzas, R., Leturcq, F., Alzualde, A., Inza, I., García-Bragado, F., De la Herran, G., Corcuera, J., Cabello, A., et al. (2008). Gene expression profiling in limb-girdle muscular dystrophy 2A. PLoS ONE *3*, e3750.

Solovei, I., Wang, A.S., Thanisch, K., Schmidt, C.S., Krebs, S., Zwerger, M., Cohen, T.V., Devys, D., Foisner, R., Peichl, L., et al. (2013). LBR and lamin A/C sequentially tether peripheral heterochromatin and inversely regulate differentiation. Cell *152*, 584–598.

Stark, D.A., Karvas, R.M., Siegel, A.L., and Cornelison, D.D. (2011). Eph/ephrin interactions modulate muscle satellite cell motility and patterning. Development *138*, 5279–5289.

Takeda, K., Inoue, H., Tanizawa, Y., Matsuzaki, Y., Oba, J., Watanabe, Y., Shinoda, K., and Oka, Y. (2001). WFS1 (Wolfram syndrome 1) gene product: predominant subcellular localization to endoplasmic reticulum in

cultured cells and neuronal expression in rat brain. Hum. Mol. Genet. *10*, 477–484.

Therizols, P., Illingworth, R.S., Courilleau, C., Boyle, S., Wood, A.J., and Bickmore, W.A. (2014). Chromatin decondensation is sufficient to alter nuclear organization in embryonic stem cells. Science *346*, 1238–1242.

Uhlén, M., Fagerberg, L., Hallström, B.M., Lindskog, C., Oksvold, P., Mardinoglu, A., Sivertsson, Å., Kampf, C., Sjöstedt, E., Asplund, A., et al. (2015). Proteomics. Tissue-based map of the human proteome. Science *347*, 1260419.

Vogel, M.J., Peric-Hupkes, D., and van Steensel, B. (2007). Detection of in vivo protein-DNA interactions using DamID in mammalian cells. Nat. Protoc. *2*, 1467–1478.

Wilkie, G.S., Korfali, N., Swanson, S.K., Malik, P., Srsen, V., Batrakou, D.G., de las Heras, J., Zuleger, N., Kerr, A.R., Florens, L., et al. (2011). Several novel nuclear envelope transmembrane proteins identified in skeletal muscle have cytoskeletal associations. Mol. Cell. Proteomics *10*, M110.003129.

Wong, X., Luperchio, T.R., and Reddy, K.L. (2014). NET gains and losses: the role of changing nuclear envelope proteomes in genome regulation. Curr. Opin. Cell Biol. *28*, 105–120.

Wu, F., and Yao, J. (2013). Spatial compartmentalization at the nuclear periphery characterized by genome-wide mapping. BMC Genomics *14*, 591.

Yaffe, D., and Saxel, O. (1977). Serial passaging and differentiation of myogenic cells isolated from dystrophic mouse muscle. Nature *270*, 725–727.

Yamada, M., Tatsumi, R., Yamanouchi, K., Hosoyama, T., Shiratsuchi, S., Sato, A., Mizunoya, W., Ikeuchi, Y., Furuse, M., and Allen, R.E. (2010). High concentrations of HGF inhibit skeletal muscle satellite cell proliferation in vitro by inducing expression of myostatin: a possible mechanism for reestablishing satellite cell quiescence in vivo. Am. J. Physiol. Cell Physiol. *298*, C465–C476.

Yazawa, M., Ferrante, C., Feng, J., Mio, K., Ogura, T., Zhang, M., Lin, P.H., Pan, Z., Komazaki, S., Kato, K., et al. (2007). TRIC channels are essential for Ca2+ handling in intracellular stores. Nature *448*, 78–82.

Yue, F., Cheng, Y., Breschi, A., Vierstra, J., Wu, W., Ryba, T., Sandstrom, R., Ma, Z., Davis, C., Pope, B.D., et al.; Mouse ENCODE Consortium (2014). A comparative encyclopedia of DNA elements in the mouse genome. Nature *515*, 355–364.

Zuleger, N., Robson, M.I., and Schirmer, E.C. (2011). The nuclear envelope as a chromatin organizer. Nucleus *2*, 339–349.

Zuleger, N., Boyle, S., Kelly, D.A., de las Heras, J.I., Lazou, V., Korfali, N., Batrakou, D.G., Randles, K.N., Morris, G.E., Harrison, D.J., et al. (2013). Specific nuclear envelope transmembrane proteins can promote the location of chromosomes to and from the nuclear periphery. Genome Biol. *14*, R14.

Zullo, J.M., Demarco, I.A., Piqué-Regi, R., Gaffney, D.J., Epstein, C.B., Spooner, C.J., Luperchio, T.R., Bernstein, B.E., Pritchard, J.K., Reddy, K.L., and Singh, H. (2012). DNA sequence-dependent compartmentalization and silencing of chromatin at the nuclear lamina. Cell *149*, 1474–1487.

Molecular Cell, Volume *62*

## Supplemental Information

# Tissue-Specific Gene Repositioning by Muscle Nuclear Membrane Proteins Enhances Repression of Critical Developmental Genes during Myogenesis

**Michael I. Robson, Jose I. de las Heras, Rafal Czapiewski, Phú Lê Thành, Daniel G. Booth, David A. Kelly, Shaun Webb, Alastair R.W. Kerr, and Eric C. Schirmer**

# SUPPLEMENTAL FIGURES
**Robson, de las Heras *et al*,.**

**A**

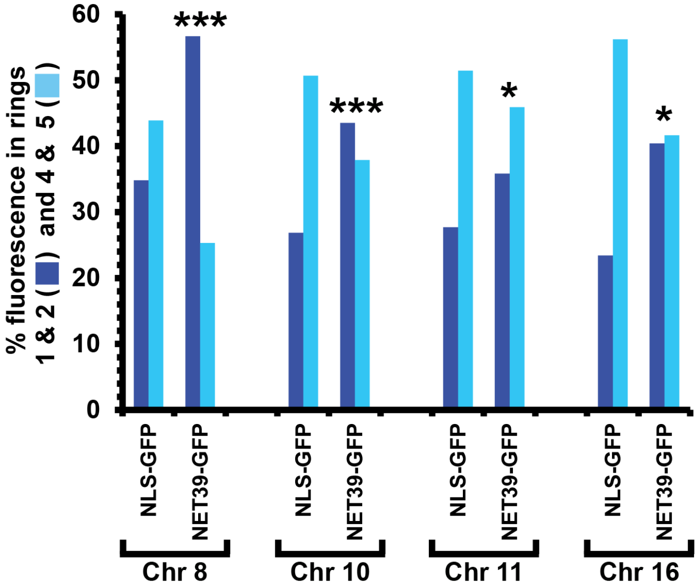


**B**

| | NT MB | NT MT | NET39 shRNA MB | NET39 shRNA MT | NET39-shRNA MB+NLS-GFP | NET39 shRNA MB+GFP-NET39 | Satellite cells | Myofibers |
|---|---|---|---|---|---|---|---|---|
| NT MB | NA | 0.000 | 0.000 | 0.000 | 0.000 | 0.005 | 0.517 | 0.000 |
| NT MT | | NA | 0.000 | 0.000 | 0.000 | 0.127 | 0.000 | 0.114 |
| NET39 shRNA MB | | | NA | 0.123 | 0.577 | 0.000 | 0.003 | 0.000 |
| NET39 shRNA MT | | | | NA | 0.004 | 0.000 | 0.000 | 0.000 |
| NET39-shRNA MB+NLS-GFP | | | | | NA | 0.000 | 0.001 | 0.000 |
| NET39 shRNA MB+GFP-NET39 | | | | | | NA | 0.001 | 0.002 |
| Satellite cells | | | | | | | NA | 0.000 |
| Myofibers | | | | | | | | NA |

P<0.05 P<0.01 P<0.001

**Figure S1. Screen to Identify Substrates for NET39-mediated Chromosome Repositioning and Statistics for Figure 1E.** GFP-NET39 was overexpressed in C2C12 MBs and the position of chromosomes 8, 10, 11 and 16 scored to determine if any are a substrate for NET39-mediated repositioning to the periphery. A. Quantitation of chromosome repositioning by NET39 overexpression in C2C12 MBs using the macro described in Figure 1D. *P < 0.05 and **P < 0.01 and ***P < 0.001 comparing the position of the chromosome in the GFP-NET39 overexpressing cells to the NLS-GFP overexpressing cells using KS tests. B. Summary table of KS tests for indicated comparisons from data in Figure 1 comparing NET39 overexpression and knockdown in both C2C12 myoblasts and myotubes. Values have been color coded based on degree of significance.

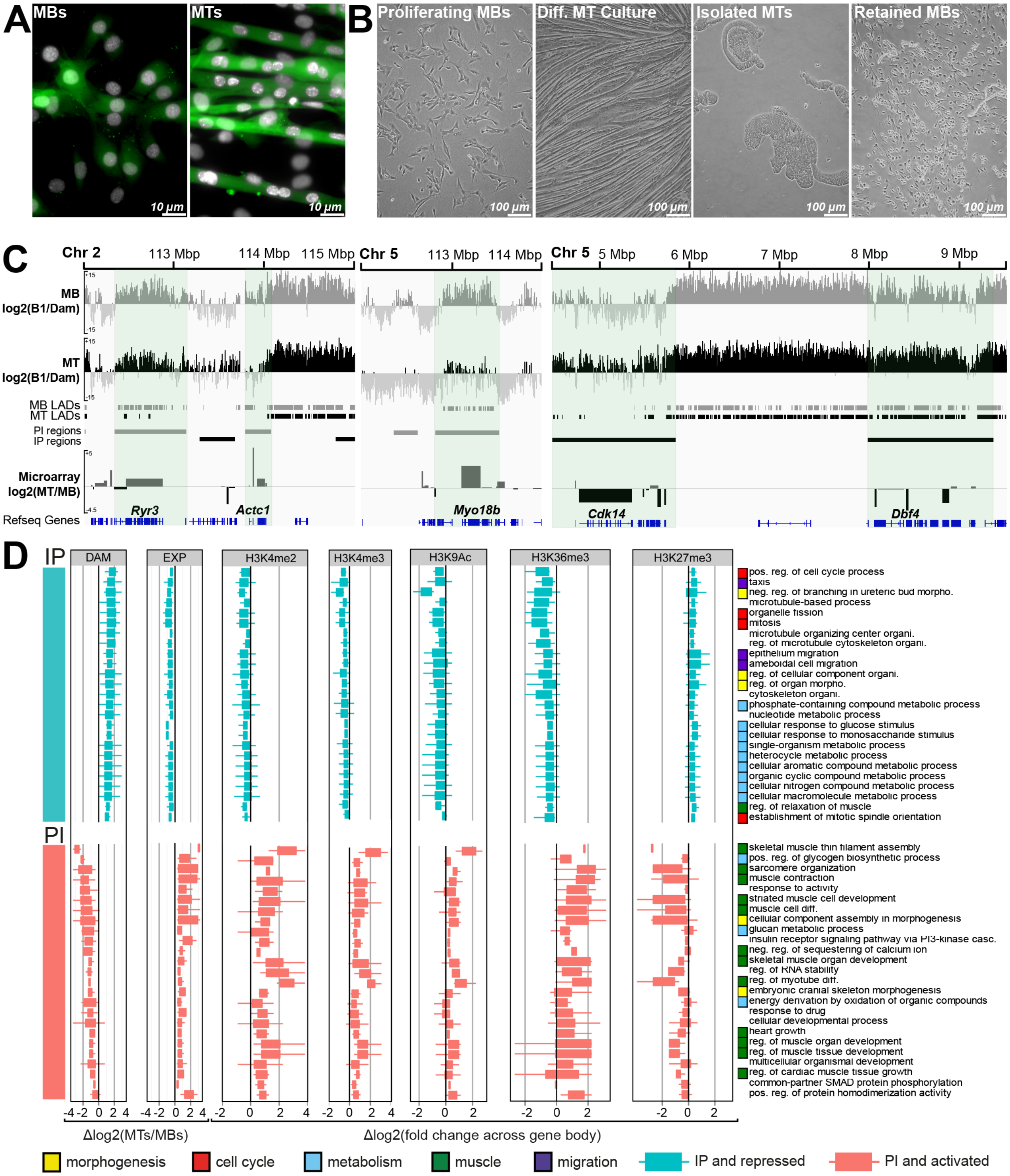


**Figure S2. Example of MT isolation and DamID Genome Browser Views with ChIP-Seq Data and Variance, Related to Figure 2.** A. Representative micrograph of C2C12 MBs and MTs transduced with a GFP-encoding lentivirus. B. Micrographs of cells pre- and post-isolation of a pure MT population. C. Genome browser views for genomic region surrounding indicated genes showing DamID signal intensities, identified LADs, IP and PI regions and microarray gene expression changes for MBs and MTs. D. Tukey boxplots (outliers not shown) displaying Δlog2 DamID, expression and Δlog(fold change) and indicated histone modifications values for genes within GO term categories significantly enriched in PI activated genes and IP repressed genes. Myogenic alterations to histone

modifications associated with transcriptionally active genes (H3K4me2, H3K4me3, H3K9Ac and H3K36me3) and the transcriptional repression-associated H3K27me3 were extracted from Asp et al., 2011. The Δlog2(DamID) value was calculated by subtracting the average log2(Lamin B1/Dam) value in a 100 kb window surrounding the gene in the MB sample from the MT sample. ChIP-seq values were determined for each histone modification by subtracting the average signal across the gene body in the MB sample from the MT sample.

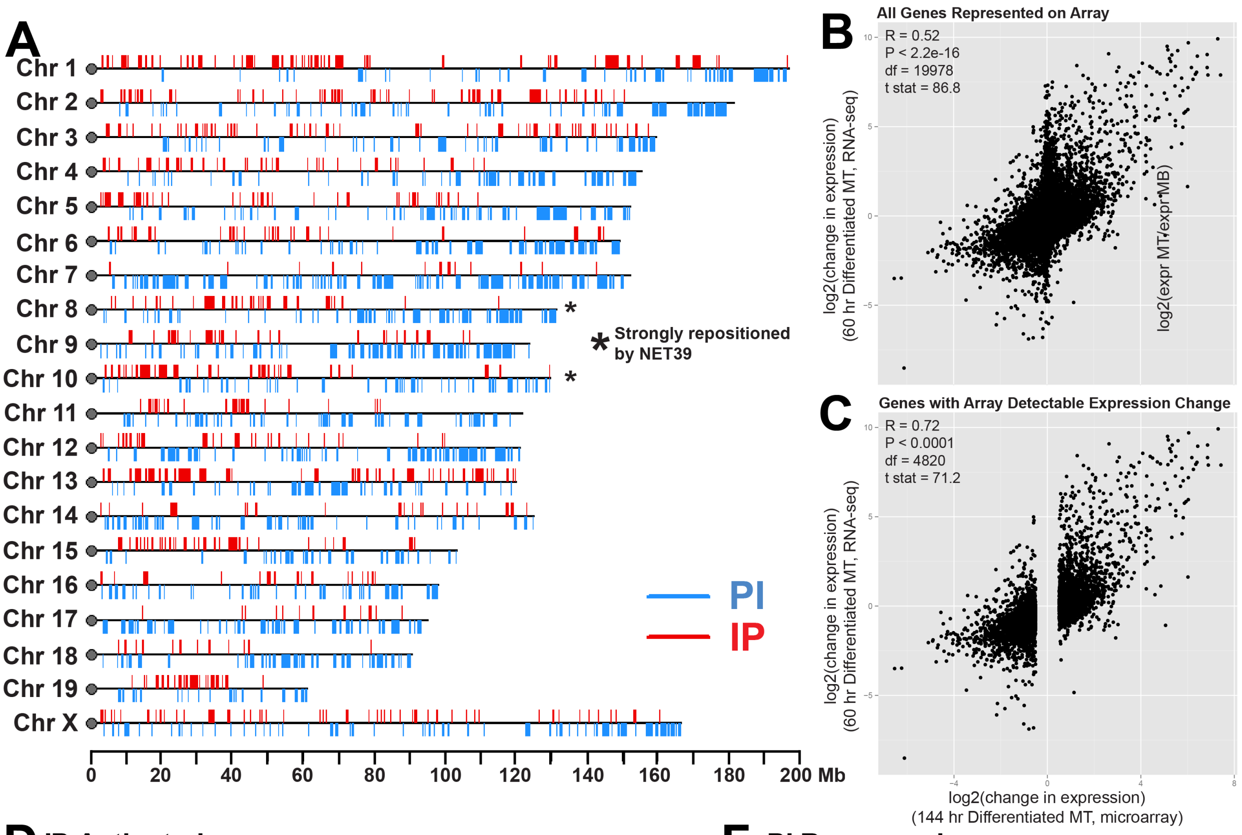


## D IP Activated

| GO term | Description | P-value | FDR q-value | Enrichment (N, B, n, b) | Genes |
|---|---|---|---|---|---|
| GO:0006334 | nucleosome assembly | 9.49E-04 | 1.00E+00 | 4.76 (2223,17,165,6) | Hist1h2bc, Hist1h2bj, Hist1h4i, Hist1h4n, Hist1h2bk, Hist1h1c, Hist1h2bm, Tspyl1 |

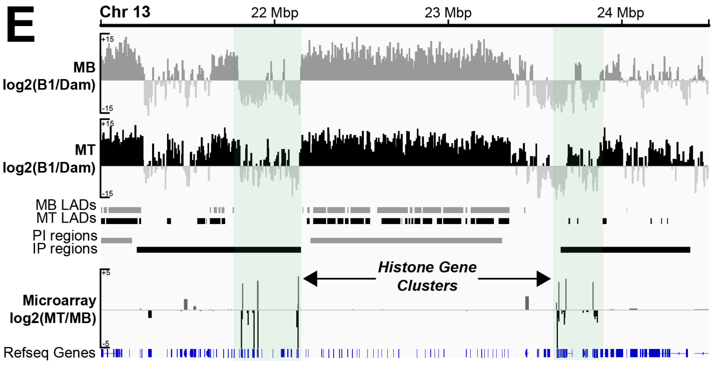


## F PI Repressed

| GO term | Description | P-value | FDR q-value | Enrichment (N, B, n, b) |
|---|---|---|---|---|
| GO:0000280 | nuclear division | 3.52E-06 | 2.72E-02 | 6.68 (2324,31,101,9) |
| GO:0051301 | cell division | 4.44E-06 | 1.72E-02 | 5.17 (2324,49,101,11) |
| GO:0007049 | cell cycle | 5.65E-06 | 1.45E-02 | 3.98 (2324,81,101,14) |
| GO:0007067 | mitotic nuclear division | 1.08E-05 | 2.08E-02 | 6.82 (2324,27,101,8) |
| GO:0048285 | organelle fission | 1.75E-05 | 2.70E-02 | 5.60 (2324,37,101,9) |
| GO:1903047 | mitotic cell cycle process | 2.96E-05 | 3.82E-02 | 4.29 (2324,59,101,11) |
| GO:0022402 | cell cycle process | 1.16E-04 | 1.28E-01 | 3.25 (2324,92,101,13) |
| GO:0006487 | protein N-linked glycosylation | 3.58E-04 | 3.46E-01 | 10.23 (2324,9,101,4) |
| GO:0016072 | rRNA metabolic process | 8.77E-04 | 7.52E-01 | 8.37 (2324,11,101,4) |

## G

P-value' is the enrichment p-value computed according to the mHG or HG model.

FDR q-value' is the correction of the above p-value for multiple testing using the Benjamini & Hochberg (1995) method. For the ith term (ranked according to p-value) the FDR q-value = (p-value * number of GO terms) / i.

Enrichment (N, B, n, b) is defined as follows:
N - is the total number of genes
B - is the total number of genes associated with a specific GO term
n - is the number of genes in the top of the user's input list or in the target set when appropriate
b - is the number of genes in the intersection
Enrichment = (b/n) / (B/N)

**Figure S3. DamID Idiograms, Microarray and RNA-Seq Comparison, and Details of GO-term Analysis in Figure 2.** A. Idiograms displaying the distribution of IP and PI regions with altered NE-association during myogenesis. * represents chromosomes which were found to be highly significantly repositioned by NET39 overexpression in C2C12 myoblasts as shown in Fig. S1. B. Comparison of C2C12 myoblast to myotube changes in gene expression detected in published RNA-Seq data from 60 hours of differentiation (Yue et al, 2014) vs our microarray analysis for 144 hour differentiated C2C12 myotubes. When the change in expression for all genes represented on the array is contrasted to the change detected by RNA-seq, a Pearson correlation of 0.52 is obtained. C. However, 75% of genes detected as “not changing” by the microarray were below the detectable microarray signal threshold, as determined from the distribution of log2(raw signal) of included negative controls (data not shown). Hence, when only comparing genes displaying altered expression during differentiation by microarray to the corresponding change detected by RNA-seq, a strong Pearson correlation of 0.72 is obtained. From this we conclude

the gene expression changes detected by our microarray are largely also detected in the RNA-seq dataset. A considerable source of the remaining deviation likely derives from the difference in time point of differentiation (144 hours for our microarray vs 60 hours for the published RNA-seq). R = correlation coefficient, P = P value, df = degrees of freedom, t statistic. D-G. GO-term Analysis for IP activated and PI repressed genes. D. List of GO-terms enriched in the set of genes which are recruited to the periphery and activated during myogenesis. Genes repositioning to the nuclear periphery concomitantly with activation display few enriched GO-terms. E. Genome browser view of the Hist1 gene cluster showing the majority of genes associated with the nucleosome assembly GO-term are located within two proximal IP regions. F. List of GO-terms enriched in the set of genes which are released from the periphery and repressed during myogenesis. In some cases genes associated with these processes require peripheral localization for transcriptional activation. G. Descriptions of table values described in D and F.

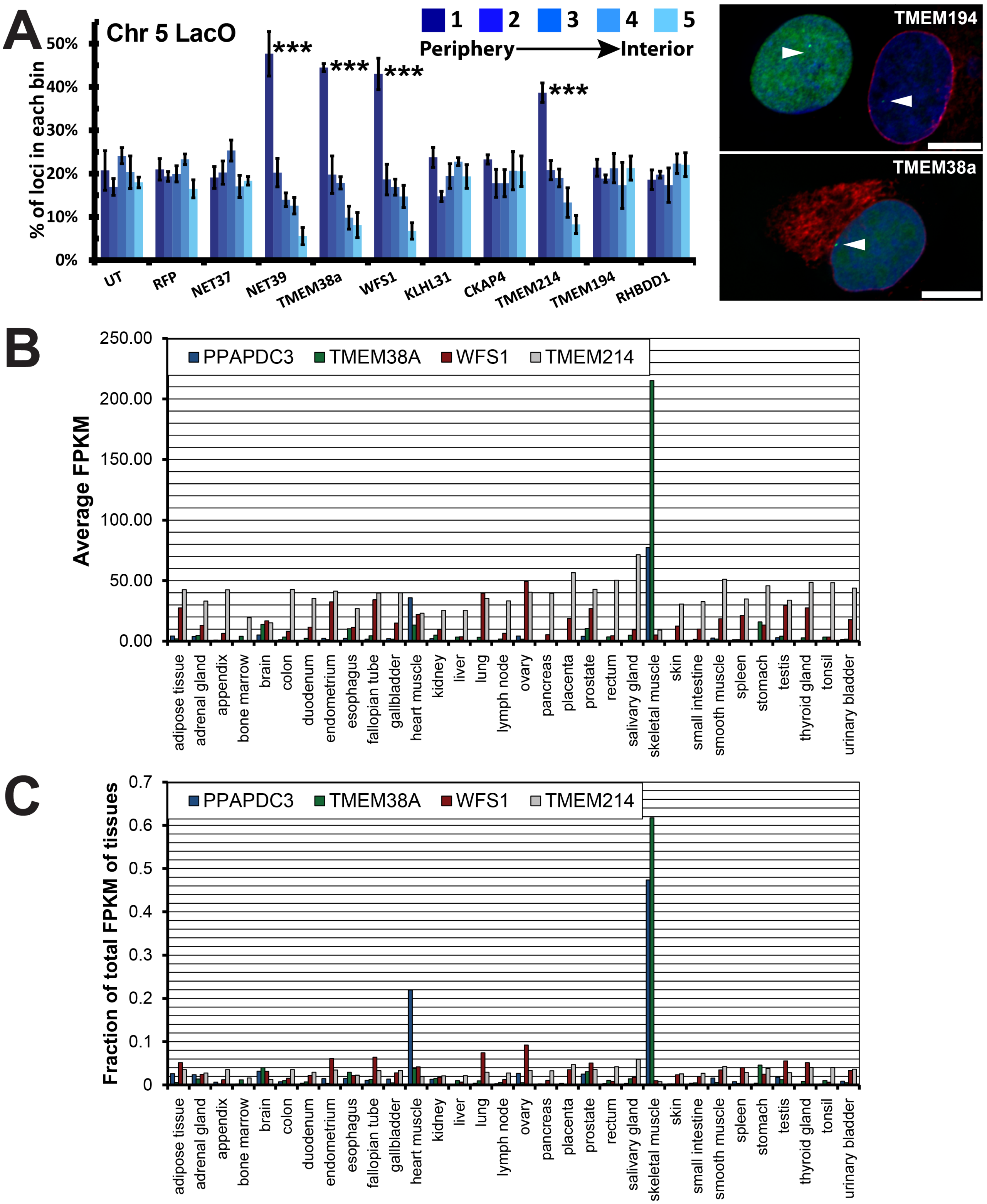


**Figure S4. Screen to Identify Additional Muscle Repositioning NETs and Human Tissue RNA-seq Transcript Data, Related to Figure 3.** A. Representative images and quantification of a lacO array integrated into chromosome 5 of HT1080 fibroblasts and used as a surrogate to measure chromosome-positioning changes when the cells overexpress NET-RFP constructs. The quantification identified 4 muscle NETs that significantly reposition chromosome 5 using the array as a marker for chromosome repositioning. Right image panels: lacI-GFP (green)

expression allows the lacO array to be visualized along with the NET-RFP protein (red) and DAPI (blue). ***P < 0.001 by comparing the peripheral to summed internal incidence of the array in the NET-transfected cells to the mRFP control using two way $\chi^2$ tests. Error bars represent standard deviation of the mean across 3 biological repeats. B and C. Extracted transcript levels across human tissues from published RNA-seq data represented as average Fragments Per kb of transcript per Million mapped reads (FPKM) values or as a fraction of the total FPKM signal across all tissues (Uhlen et al., 2015). NET39 and TMEM38a are highly expressed in skeletal muscle while WFS1 and TMEM214 are more ubiquitously expressed, although targeting to the NE was restricted to muscle (Figure 3).

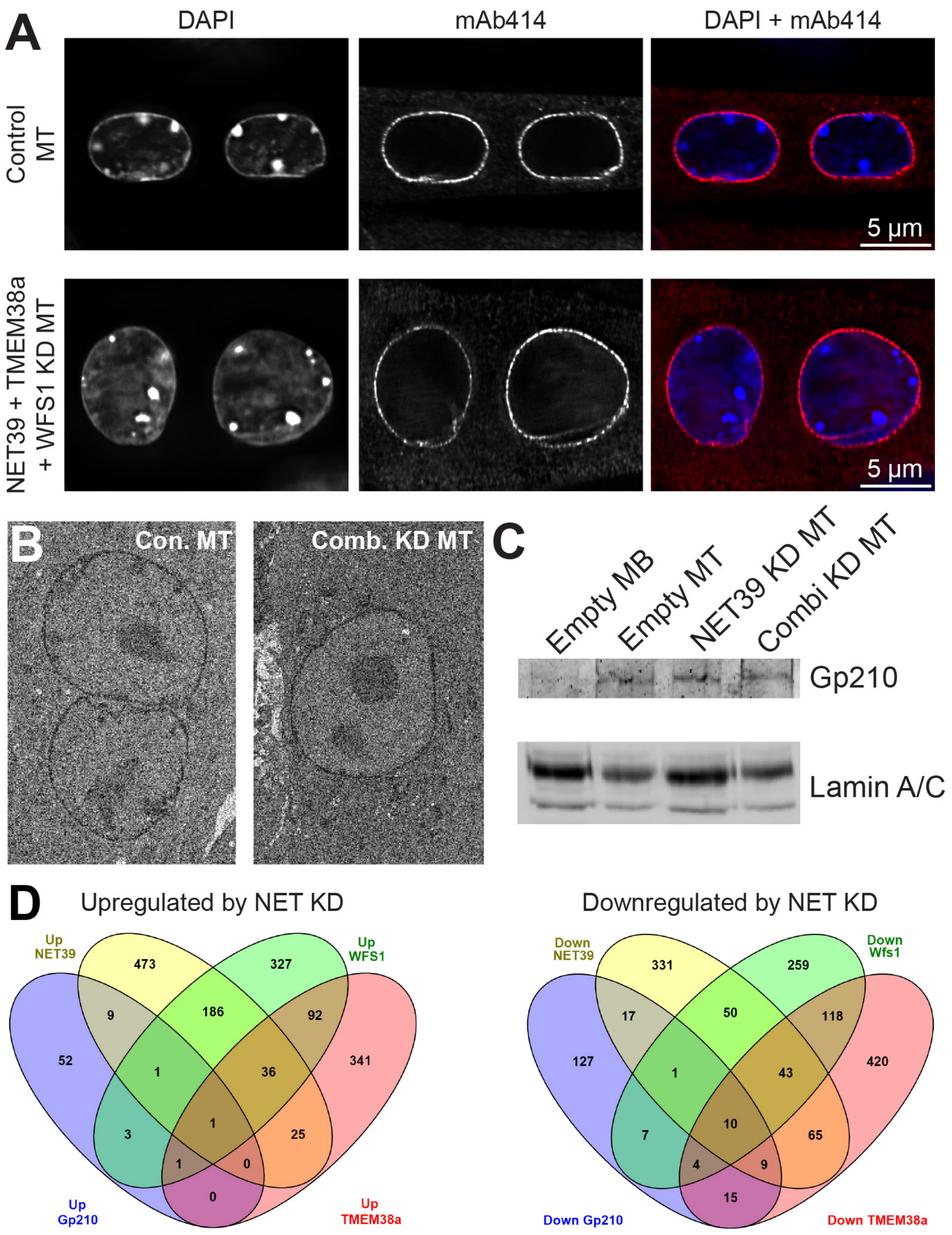


**Figure S5. NPC and Gp210 (Nup210) are Unperturbed in Chromosome Repositioning NET Depleted Cells, Related to Figure 4.** A. Representative micrographs demonstrating relative levels and distribution of NPCs by staining of FG-containing nucleoporins. B. 3D-View electron micrographs of Control and Triple-knockdown MT nuclei. In 20 nuclei observed for each sample, no increased NE ruptures or disruptions were observed. C. Western blot displaying unaltered levels of Gp210 induction and Lamin A/C expression in MBs, empty vector-treated MTs, NET39 shRNA-treated MTs or MTs co-treated with NET39, TMEM38a and WFS1 shRNAs. D. Non-proportional Venn diagrams displaying overlap between genes up- or down-regulated in Gp210-, NET39-, TMEM38a- or WFS1-depleted myotubes. The majority of genes affected by Gp210 are distinct from those affected by individual depletion of chromosome repositioning NETs. Gp210 gene expression data taken from D'Angelo et al 2012.

**A**

P<0.05 | P<0.01 | P<0.001

| Nid1 | Empty MB + GFP-NET39 | Empty MT | NET39 kd MT |
|---|---|---|---|
| Empty MB | 0.000 | 0.000 | 0.008 |
| Empty MB + GFP-NET39 | | 0.182 | 0.078 |
| Empty MT | | | 0.004 |

| Ptn | Empty MB + GFP-NET39 | Empty MT | NET39 kd MT |
|---|---|---|---|
| Empty MB | 0.000 | 0.000 | 0.247 |
| Empty MB + GFP-NET39 | | 0.036 | 0.003 |
| Empty MT | | | 0.000 |

| Msc | Empty MB + GFP-NET39 | Empty MT | NET39 kd MT |
|---|---|---|---|
| Empty MB | 0.000 | 0.000 | 0.120 |
| Empty MB + GFP-NET39 | | 0.141 | 0.000 |
| Empty MT | | | 0.000 |

| DDR2 | Empty MB + TMEM38a-GFP | Empty MT | TMEM38a kd MT |
|---|---|---|---|
| Empty MB | 0.000 | 0.000 | 0.193 |
| Empty MB + TMEM38a-GFP | | 0.541 | 0.000 |
| Empty MT | | | 0.000 |

| Cxcl1 | Empty MB + WFS1-GFP | Empty MT | WFS1 kd MT |
|---|---|---|---|
| Empty MB | 0.001 | 1.000 | 0.000 |
| Empty MB + WFS1-GFP | | 0.015 | 0.043 |
| Empty MT | | | 0.000 |

| Vcam1 | Empty MB + GFP-NET39 | Empty MB + WFS1-GFP | Empty MT | NET39 kd MT | WFS1 kd MT |
|---|---|---|---|---|---|
| Empty MB | 0.000 | 0.002 | 0.000 | 0.383 | 0.004 |
| Empty MB + GFP-NET39 | | 0.260 | 0.369 | 0.001 | 0.165 |
| Empty MB + WFS1-GFP | | | 0.042 | 0.040 | 0.881 |
| Empty MT | | | | 0.000 | 0.023 |
| NET39 kd MT | | | | | 0.062 |

| Bdnf | Empty MB + GFP-NET39 | Empty MB + WFS1-GFP | Empty MT | NET39 kd MT | WFS1 kd MT |
|---|---|---|---|---|---|
| Empty MB | 0.353 | 0.001 | 0.038 | 0.034 | 0.727 |
| Empty MB + GFP-NET39 | | 0.013 | 0.280 | 0.234 | 0.184 |
| Empty MB + WFS1-GFP | | | 0.224 | 0.406 | 0.000 |
| Empty MT | | | | 0.862 | 0.026 |
| NET39 kd MT | | | | | 0.016 |

| Efna5 | Empty MB + GFP-NET39 | Empty MB + TMEM38a-GFP | Empty MT | NET39 kd MT | TMEM38a kd MT |
|---|---|---|---|---|---|
| Empty MB | 0.677 | 0.094 | 0.002 | 1.000 | 0.604 |
| Empty MB + GFP-NET39 | | 0.309 | 0.028 | 0.725 | 0.371 |
| Empty MB + TMEM38a-GFP | | | 0.245 | 0.188 | 0.038 |
| Empty MT | | | | 0.019 | 0.001 |
| NET39 kd MT | | | | | 0.732 |

**B**

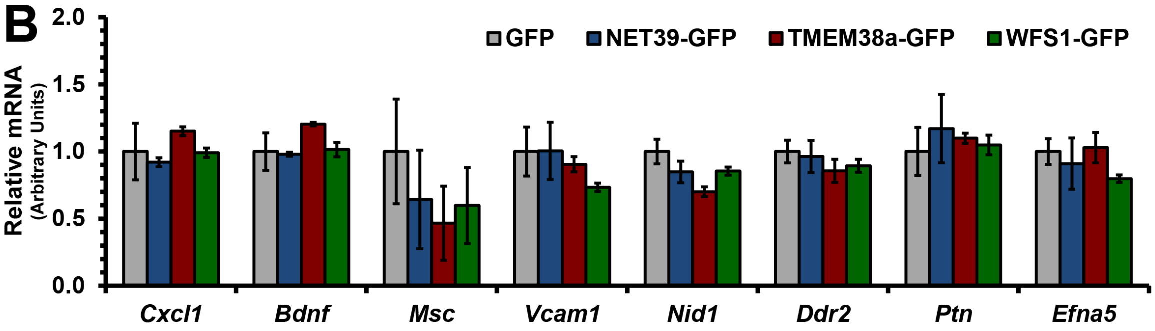


**C**

| Two Tailed t-test | *Cxcl1* | *Bdnf* | *Msc* | *Vcam1* | *Nid1* | *Ddr2* | *Ptn* | *Efna5* |
|---|---|---|---|---|---|---|---|---|
| GFP:NET39-GFP | 0.805 | 0.878 | 0.171 | 0.988 | 0.147 | 0.747 | 0.647 | 0.580 |
| GFP:TMEM38a-GFP | 0.644 | 0.181 | 0.082 | 0.677 | 0.054 | 0.177 | 0.653 | 0.797 |
| GFP:WFS1-GFP | 0.977 | 0.918 | 0.095 | 0.264 | 0.250 | 0.208 | 0.833 | 0.054 |

P<0.05
P<0.01
P<0.001

**Figure S6. Summary of FISH Statistics for Figure 5 and qPCR of NET-Regulated Genes in C2C12 MBs.** A. All statistical comparisons to determine the significance of repositioning for each gene calculated by $\chi^2$ tests with degree of significance highlighted. Tests compared the peripheral to summed internal incidence of the locus in the indicated samples. B. qPCR of NET-regulated genes in MBs stably overexpressing GFP-tagged NETs. For each gene tested here, one of the NETs is normally needed for its repositioning and silencing at the periphery in MTs. However, the NETs could also reposition the genes to the periphery in MBs in the absence of differentiation when stably overexpressed (see Figure 5). However, NET-induced peripheral targeting in the absence of differentiation is is insufficient to repress target genes. As a control, samples were normalized to TMEM194, a NET that had no effect in the repositioning assay (Figure 3). Error bars represent standard deviation across 3 biological repeats. C. Table of statistical comparisons to determine the significance of gene expression changes for each gene calculated by Two Tailed t-test. No significant differences were observed.

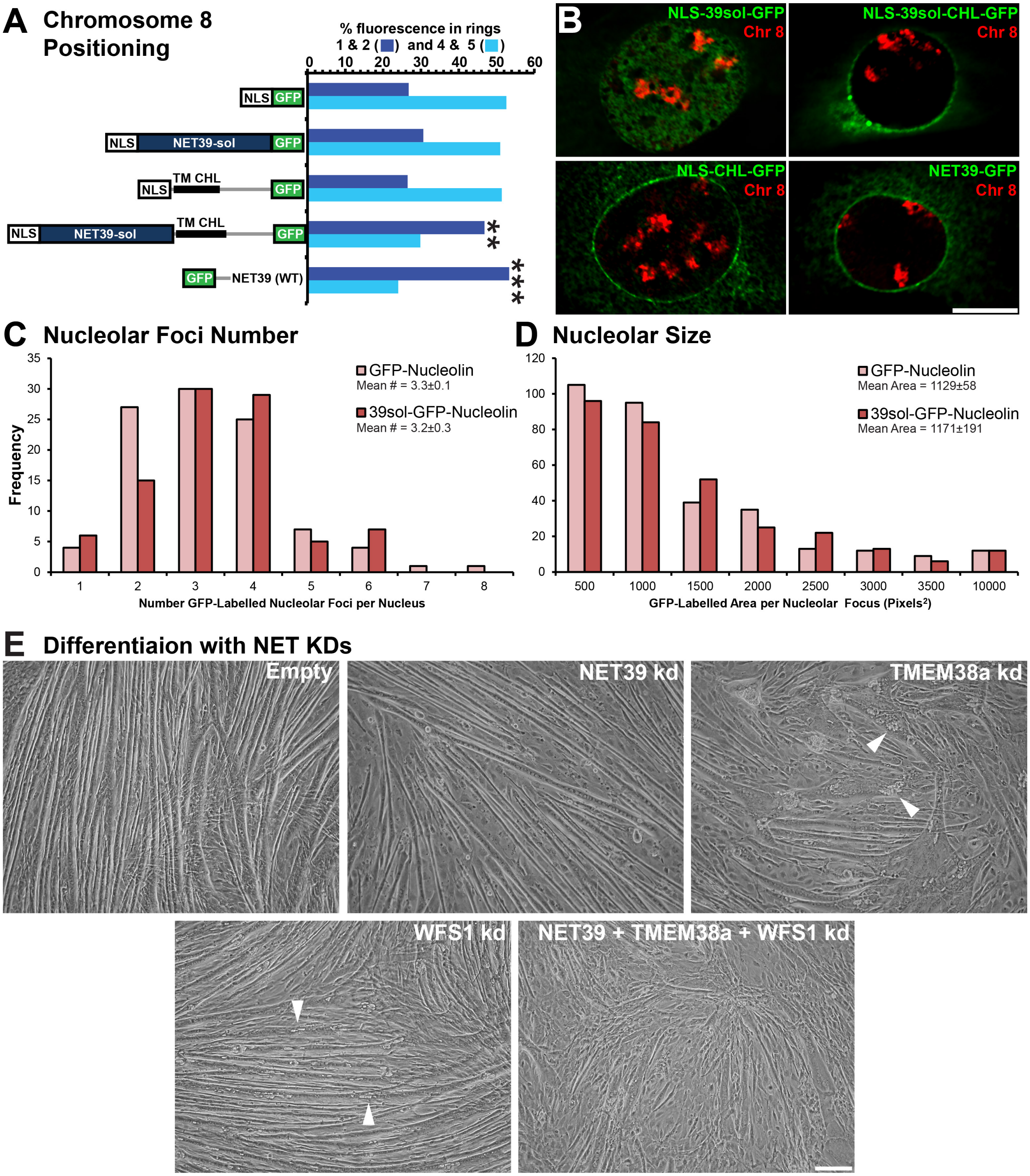


**Figure S7. NET39sol Domain Construct Effects on Chromosome and Gene Position and Differentiation of NET KD MBs, Related to Figures 6 and 7.** A and B. Re-anchoring the NET39sol domain rescues NET39 chromosome 8 repositioning, related to Figure 6. A. Quantification of chromosome 8 position across 50-100 cells in indicated conditions. Chromosome 8 is repositioned to the periphery only when the 39sol domain is anchored to the periphery. Scale bar is 5 µm. $^{**}P < 0.01$ and $^{***}P < 0.001$ comparing the position of the chromosome in the indicated overexpressing cells to the NLS-GFP overexpressing cells using KS tests. B. Example images of chromosome 8 position in NET39-shRNA MBs expressing the indicated constructs. C and D. The Number and Size of GFP-labelled Nucleoli Foci is Unaffected in NET39sol-GFP-Nucleolin Expressing Cells. C. Frequency histogram of the number of

nucleolar foci per nucleus with average size. D. Frequency histogram of the distribution of areas for GFP-labelled nucleolar foci. In both C and D the error margin for a given mean number or the area of a given nucleolar foci reflects the standard deviation between the means of two biological replicates. E. NET-depletion significantly perturbs the morphology and extent of differentiation of MTs, Related to Figure 7. Micrographs of knockdown MTs. Confluent plates of control MBs and NET39, TMEM38a and WFS1 knockdown MBs were induced to differentiate for 6 days. Control MBs form elongated, multinucleated MTs that align in linear arrays. Although NET39-depleted MTs were normal in appearance they were fewer in number. TMEM38a-depleted MTs were smaller and contained vacuoles indicative of large scale damage (indicated by arrows). WFS1-depleted MTs had less of a pronounced phenotype but also displayed visible vacuoles. Combinatorial NET39, TMEM38a and WFS1-depletion massively impaired myogenesis, generating large deformed myotubes that were significantly reduced in number suggesting the loss of each NET has an additive effect. Scale bar 100 µm.

**Supplemental Movie 1. NET Depletion Significantly Alters the Kinetics of Myogenesis, Related to Figure 7.** Time-lapse of Empty-, NET39-, TMEM38a-, WFS1- and combinatorial-shRNA treated MBs differentiating to myotubes over ~5 days using the 10x objective of the IncuCyte ZOOM® system. The kinetics of NET39, TMEM38a and combinatorial knockdown MBs differentiation is severely impaired while the myotubes formed by TMEM38a and cells with the combinatorial knockdown are misshapen and poorly aligned.

**Supplemental Table 1. Table of DamID and Gene Expression Changes in C2C12 cells upon Myogenesis and NET-depletion, Related to Figures 2, 3, 4 and 5.** Information for all genes represented on microarray for gene expression and DamID results presented as summary and raw data values. The table contains a key explaining the various parameters.

# SUPPLEMENTAL EXPERIMENTAL PROCEDURES
**Robson, de las Heras *et al,*.**

## Antibodies

Primary antibodies and their sources are listed below. For visualization of primary antibodies for immunofluorescence, donkey anti-mouse, anti-rabbit and anti-guinea pig secondary antibodies conjugated to a variety of Alexa Fluor® dyes were used (Molecular Probes, Invitrogen). For the detection of biotin- or digoxigenin-labelled probes in FISH experiments, streptavidin (Molecular Probes, Invitrogen) or anti-digoxigenin antibodies (Jackson labs) conjugated to Alexa Fluor® dyes were used, respectively. For Licor Western blotting, IRDye®-conjugated anti-mouse or anti-rabbit antibodies were used (Licor).

**List of Primary Antibodies.**

| Antigen | Host | IF dilution | WB dilution | Band Size | Source |
|---:|---:|---:|---:|---:|---:|
| **H3** | Mouse | N/A | 1:200-1:1000 | 17 kDa | Abcam (10799) |
| **Myh1** | Mouse | 1:200 | 1:500-1:1000 | 200 kDa | Sigma (M1570) clone My-32 |
| **Lamin A/C** | Guinee pig | 1:200 | 1:1000 | 72 kDa | (Schirmer et al., 2001) |
| **Lamin B1** | Guinee pig | 1:400 | 1:1000 | 66 kDa | (Schirmer et al., 2001) |
| **NET39** | Rabbit | N/A | 1:200 | 30 kDa | Proteintech (20635-1-AP) |
| **TMEM38a** | Rabbit | 1:100 | 1:200 | 30 kDa | Millipore (06-1005) |
| **WFS1** | Rabbit | 1:50 | 1:200 | 100 kDa | Proteintech (11558-1-AP) |
| **TMEM214** | Rabbit | 1:50 | 1:200 | 70 kDa | Proteintech (20125-1-A) |
| **Dystrophin** | Mouse | 1:50 | N/A | 271 kDa | Glenn Morris (MANDYS1(3B7)) |
| **V5** | Mouse | 1:200 | 1:200 | N/A | Invitrogen (R960-25) |
| **GFP** | Rabbit | 1:200 | 1:1000 | N/A | Invitrogen (A-11122) |
| **RFP** | Mouse | 1:200 | 1:1000 | N/A | Generated by Dzmitry Batrakou |
| **NPC Mixture** | Mouse | 1:500 | N/A | N/A | Abcam (ab24609) |
| **Gp210** | Guinee pig | 1:200 | 1:500 | 250 kDa | Greber (1990) |

## Plasmid Sources and Construction

Expression constructs for human NET39/PPAPDC3, Tmem38a, WFS1 and Tmem214 were cloned previously by the Schirmer laboratory (Wilkie et al., 2011; Zuleger et al., 2011). Notably, the NET39 variant used in those works and in this one is a longer splice variant than that in the original paper describing this NET (Schirmer et al., 2003). NET depletion experiments employed the pLKO shRNA lentiviral vectors TRCN0000081370 (5'-AGGCTTCATCATCGGCTACTT-3'), TRCN0000124329 (5'-GCCTTCTGTTTCTGTTCTGTA-3') and

TRCN0000248037 (5'ATGCCGAGTGTCATAAGAAAT3') targeting NET39, TMEM38a and WFS1, respectively (Sigma). The empty and non-target shRNA vectors SHC001 and SHC002 were used as reference controls (Sigma). To stably overexpress NETs in the absence of differentiation, GFP, GFP-NET39, TMEM38a-GFP or WFS1-GFP were cloned into a modified pLVX-TRE3G lentiviral vector (Clontech). In this vector, GFP constructs are doxycycline-inducible in pLVX-TET3G treated cells. Doxycyclin-inducible puromycin resistance is achieved simultaneously via an intervening IRES. The lentiviral packaging constructs psPAX2 and pMD2.G were a gift from Justina Cholewa-Waclaw (Adrian Bird Laboratory, WTCCB, Edinburgh).

For nuclear envelope retargeting experiments the 24-131 amino acid fragment containing the previously published transmembrane of chicken hepatic lectin (CHL) (Soullam and Worman, 1993) was inserted into the NLS-39sol-eGFP N3 construct described in Zuleger et al,. 2013. For nucleolin retargeting experiments the NET39 soluble fragment was inserted into a previously published GFP-nucleolin vector (Takagi et al., 2005). pSVK3-3 CHL was a gift from the Worman lab, and GFP-nucleolin a gift from Michael Kastan (Addgene plasmid # 28176).

## Primary Myofiber and Satellite Cell Isolation

Myofibers and satellite cells were isolated from the Extensor Digitorum Longus (EDL) muscle of 6-8 week old male J6 mice as described by Moyle & Zammit and demonstrated visually by Pasut et al. (Moyle & Zammit, 2014; Pasut et al, 2013). Briefly, four 6-8 week old male J6 mice (Central Bioresearch Services, University of Edinburgh) were sacrificed by cervical dislocation and the EDL muscle extracted for dissociation into pre-warmed DMEM supplemented with 0.2% collagenase Type IV (Gibco, 17104-019) and antibiotics for 1.5 – 2 h at 37°C. Individual myofibers were dissociated from the muscle body by titration and separated from the remaining collagenase. For FISH, myofibers were transferred onto coverslips coated in matrigel (Gibco, A1413202), left to adhere for 4 h and then fixed. To isolate satellite cells, myofibers were plated in matrigel-coated 6 well plates for 72 h in DMEM supplemented with 20% FBS, 10% horse serum (Gibco), 1% chick embryo extract and 10 ng/ml basic Fibroblast Growth Factor (PeproTech, 450-33B), 100 units/ml penicillin, 100 μg/ml streptomycin and 10 μg/ml gentamicin. During this time associated satellite cells migrated onto the matrigel-coated plate and proliferated. To remove contaminating fibroblasts cells were trypsinized, pelleted and resuspended in satellite cell medium in a 35 mm dish to settle on the substratum. As fibroblasts but not satellite cells adhere efficiently in the absence of matrigel, after 15 min the media containing any non-adhered satellite cells was removed and cells re-plated. For FISH, satellite cells were then cultured and fixed on matrigel-coated coverslips. To generate MTs, satellite cells cultured on matrigel-coated coverslips were induced to differentiate at confluency using differentiation media as described for C2C12 MBs. Primary MTs were then fixed in 4% formaldehyde, PBS and stained as described below for tissue sections.

## Lentivirus Generation and Transduction

Lentiviruses encoding DamID or pLKO constructs were generated as described in (Salmon and Trono, 2007) with several modifications. Briefly, non-replicative lentiviruses were generated in ~6 million 293FT cells plated in a 8.5 cm diameter tissue culture plate by joint transfection of 2.8 μg pMD2.G, 4.6 μg psPAX2 and 7.5 μg of the construct-specific transfer vector using 36 μl lipofectamine 2000 in 3 ml Optimem as per the manufacturer's instructions. After 16 h 293FT media was replaced. 48 h later the virus containing supernatant was aspirated, cleared of cellular debris by centrifugation for 10 min at 3,500 rpm and followed by filtration through a 0.45 $\mu m^2$ low protein binding PES syringe filter (Millipore, SLHP003RS). Viruses were then concentrated by ultracentrifugation at 55,000 x *g* for 75 min at $4^oC$ in a JA-25.5 rotor and resuspended in an appropriate volume of Opti-mEM. If not used immediately, aliquots were frozen at $-80^{\circ}C$. Transduction was performed in the presence of 10 μg/ml protoamine sulphate. When required, selection was applied 48 h post transduction by addition of 1.5 μg/ml puromycin.

## DamID

DamID was performed as described in (Vogel et al., 2007). Briefly, for undifferentiated samples 40,000 C2C12 MBs were plated 8 h before transduction onto 3.48 cm diameter tissue culture plates and then, following adhesion to the substratum, were transduced with $^{1}/_{10}$ of a Dam methylase encoding virus's preparation in the presence of 10 µg/ml protoamine sulphate in growth medium (Dulbecco's modified Eagle's medium (DMEM) supplemented with 20% fetal calf serum (FBS), 100 units/ml penicillin and 100 µg/ml streptomycin). Transduction was performed overnight, after which virus containing media was replaced and cells left in culture for a further 48 h.

For differentiated samples, 5 days prior to transduction, C2C12 MBs were plated onto 3.48 cm tissue culture plates at 45,000 cells/$cm^2$ in growth medium. 48 h later media was aspirated, cells were washed twice in PBS and differentiation media (DMEM, 2% horse serum (Gibco), 100 units/ml penicillin and 100 µg/ml streptomycin) was added. Media was then replaced 48 h later. Following 72 h of differentiation, $^{1}/_{2}$ of a DamID viral preparation was added in the presence of 10 µg/ml protamine sulphate and 1 µM tetrodotoxin (TTX) (Toxin technologies) to prevent MT contractions. After 24 h, viral containing media was replaced with fresh differentiation media and cells left to differentiate for a further 48 h. For both undifferentiated and differentiated samples, cells were harvested by trypsinization 72 h post-transduction. For differentiated MTs partial trypsinization was performed to selectively enrich the MT population. Briefly, differentiation media was aspirated and cells subsequently washed once in PBS. TrypLE™ Express trypsin diluted 1:5 in PBS was then incubated with washed cells for 1 min at 37°C or until MTs, but not mono-nuclear MBs, were released from the growth surface, after which differentiation medium was added to block further trypsin activity and cell detachment. MTs were then carefully extracted by aspirating the cell-containing supernatant after which plates were gently washed in further differentiation medium to gather remaining MTs. To further deplete the sample of MBs, MTs were then preferentially pelleted by limited centrifugation at 500 rpm for 1 min.

DamID sample processing was then performed as described in Vogel et al. Briefly, DNA was extracted from cells using the DNeasy tissue lysis kit (Qiagen) as per manufacturer's instructions. 2.5 µg of extracted DNA was then digested by *DpnI* (NEB) and, following heat inactivation of *DpnI,* was ligated to the DamID adaptor duplex (dsAdR) generated from the oligonucleotides AdRt (5'-CTAATACGACTCACATAGGGCAGCGTGGTCGCGGCCGA-GGA-3') and AdRb (5'-TCCTCGGCCG-3') after which DNA was further digested by *DpnII*. To amplify DNA sequences methylated by the Dam methylase, 5 µl of *DpnII* digested material was then subjected to PCR in the supplied buffer in the presence of the 1.25 µM Adr-PCR primer (5'-GGTCGCGGCCGAGGATC-3'), 0.2 mM dNTPs and 1X of the Advantage cDNA polymerase (Clontech, cat. no. 639105). PCR was performed as described below.

**PCR program for DNA amplification in DamID.**

| Cycle | Denature | Anneal | Extend |
|---|---|---|---|
| 1 | | | 68°C for 10 min |
| 2 | 94°C for 3 min | 65°C for 5 min | 68°C for 15 min |
| 3-6 | 94°C for 1 min | 65°C for 1 min | 68°C for 10 min |
| 7-23 | 94°C for 1 min | 65°C for 1 min | 68°C for 2 min |

Following PCR, the quality of amplified DNA was confirmed on 2.5% agarose gels. If samples were of sufficient quality DNA was purified on QIAquick PCR purification columns (Qiagen) and then concentrated to the required concentration by precipitation. An average of 6-8 PCR reactions were required per sample to generate the 2 µg of material required of next generation sequencing.

## DamID Sequencing and Analysis

DamID sample libraries were prepared for next generation sequencing by fragmentation followed by ligation to sequencing adaptors. Libraries were then sequenced by 90 bp paired end (90PE) sequencing in reactions with 5 samples per well. As mitochondrial DNA also becomes Dam-methylated and muscle has many mitochondria, ~73% of MT reads were mitochondrial. Thus, 310 million total reads were engaged for the MT lamin B1-dam sample to achieve 80.6 million reads after subtracting mitochondrial sequences. Between 77-100 million reads were achieved for MT Dam and MB and MT Dam-Lamin B1 samples. DamID sequences were aligned to the mouse mm9 genome using the Burrows-Wheeler Aligner software bwa-mem (Li and Durbin, 2009). Subsequent processing was performed using R and Bedtools (Quinlan and Hall, 2010; R Development Core Team, 2010). MB and MT DamID data was quantified at a DpnI fragment level by counting the number of reads that overlapped each DpnI-flanked (GATC) genomic fragment for each pair of Dam-alone and Dam-LaminB1 samples, and expressed as a proportion of the total to account for variation in the number of reads. The log2 ratios between Dam-LaminB1 and Dam-alone were calculated for each DpnI fragment, and the resulting values quantile normalized in R using the BioConductor Limma package (Ritchie et al., 2015) to allow a more quantifiable sample comparison.

## Lamina Associated Domains (LADs) Definition

To identify LADs, the peakfinder software SICER (Wu and Yao, 2013; Zang et al., 2009) was employed with the following parameters: redundancy threshold 1, window size 500 bp, gap size 0, fragment size 150 bp, effective genome fraction 0.75, FDR 0.05. This identified a large number of small peaks with variable density distribution, roughly corresponding to the intensity of the LADs. We considered a gene being in a LAD if it overlapped with at least one SICER peak.

## Identification of Genomic Regions with Altered Peripheral Association

To identify genomic regions with differential frequencies of association with the periphery in MBs vs MTs, a statistical test was devised in which a moving window of 250 DpnI fragments (~100Kb) were run along each chromosome, with a shift of 20 DpnI fragments (~10Kb). From this the average DamID signal the difference between MTs and MBs was calculated within each window. Windows with a signal difference greater than 2-fold were flagged as potential differential regions. These regions were then tested for statistical significance compared to a shuffled sample (x1,000 iterations) using Fisher's exact test. The regions that passed with $p<0.01$ were termed PI or IP regions, to denote regions that exhibited a significant repositioning to the interior (PI) or to the periphery (IP) during differentiation from MBs to MTs.

## Gene Ontology and ChIP-seq Analysis

Genes in the IP class with reduced expression and genes in the PI class with increased expression were analyzed for Biological Process and Cellular Compartment GO-term enrichment using the BioConductor package GOstats (Falcon and Gentleman, 2007). The log2 change in DamID and microarray gene expression values and fold enrichment in ChIP-seq values between MTs and MBs was determined for genes within each of the top 25 enriched terms for both IP and PI classes and plotted accordingly. For DamID the log2 change in lamin B1 signal intensity for each gene represented an average value calculated for a 100 kb window surrounding the gene. For ChIP-seq, the fold enrichment between MTs and MBs was determined across the whole gene body including introns and exons. For ChIP-seq there was only limited information available for C2C12 differentiation, with only H3K4me1, H3K4me2, H3K4me3, H3K27me3, H3K36me3, H3K9Ac, H3K18Ac and H4K12Ac datasets available. These datasets were extracted from GSE25308 and quantification of fold enrichment across gene bodies achieved as described previously (Asp et al., 2011).

## RNA-seq Analysis

RNA-seq expression values were obtained from published ENCODE data (GSM929774 and GSM929775) for C2C12 differentiation by averaging the Fragments Per kb of transcript per Million mapped reads (FPKM) signal across exons for each gene available using Bedtools (Yue et al., 2014). For tissue-wide expression of NETs data were extracted from Uhlen et al., 2015.

## RNA Extraction

RNA extraction was achieved using TRI-reagent (Invitrogen) as per the manufacturer's instructions. For differentiated C2C12 cells, MTs were isolated from undifferentiated MBs by partial trypsinization and centrifugation as described previously. The quality and concentration of the RNA was then assessed using a NanoDrop 2000c spectrophotometer and an Agilent Bioanalyzer (RNA 6000 Nano total RNA kit, Agilent). If not immediately used, RNA was stored at -80°C in separate aliquots to reduce freeze thawing.

## Microarrays

RNA was labelled with biotin using the TotalPrep RNA Amplification Kit (Illumina) following the manufacturer's instructions. The quality of cRNA was then confirmed by an Agilent Bioanalyzer using the RNA 6000 Nano mRNA RNA kit (Agilent). Quality analysis and hybridization of the cRNA was performed at the Wellcome Trust Clinical Research Facility in Edinburgh. The subsequent microarray data were normalized using the free statistics package R (R Development Core Team, 2010). For each analysis, at least three biological replicates were hybridized to Illumina whole genome gene expression arrays (MouseWD6 BeadChip). These arrays have coverage of 48,804 transcripts according to the NCBI RefSeq database release 17 and UniGene release 188. Hybridizations were carried out using an Illumina Beadstation. Microarray data were quantile normalized and analyzed in the R environment using the Bioconductor package Limma (Smyth, 2005). Differentially expressed transcripts were selected with a log2 ratio above 0.5 in absolute value using moderated F-statistics adjusted for a false discovery rate of 5% (Benjamini and Hochberg, 1995).

## Quantitative Real Time PCR (qPCR)

cDNAs were generated using the Thermoscript II RNAse H– Reverse Transcriptase as per manufacturer's instructions. Briefly, following annealing to a polyA primer at 65°C, 5 µg of total RNA was incubated with 1X Thermoscript RNAse H- reaction buffer, 10 U RNAsin, 10 mM DTT, 1 µM dNTPs mix and 200 U Thermoscript II RNAse H– Reverse Transcriptase. Tubes were vortexed, centrifuged briefly, and incubated at 42°C in a thermocycler with the heated lid at 80°C for 2 h. After heat inactivation of the reverse transcriptase by a 15 min incubation at 70°C, RNA was removed by incubating samples with 1 µL of 7 mg/ml RNAse A for 45 min at 37°C. cDNA was diluted with 90 µl of water and the reactions stored at -20°C.

**qPCR Program for Lightcycler 480.**

| Target (°C) | Acquisition mode | Hold (mm:ss) | Ramp rate (°C/s) | Acquisition (per °C) |
|---|---|---|---|---|
| **Preincubation, 1 cycle** | | | | |
| **95** | | 05:00 | | |
| **Amplification, 40 cycles** | | | | |
| **95** | | 00:10 | | |
| **56** | | 00:01 | | |
| **51** | | 00:015 | | |
| **72** | Single | 00:21 | | |
| **Melting curve, 1 cycle** | | | | |
| **95** | | 00:05 | | |
| **65** | Continuous | 01:00 | 2.2 | |
| **97** | | | 0.19 | 3 |
| **Cooling, 1 cycle** | | | | |
| **22** | | | 2.2 | |

For qPCR, 20 µl reactions containing 8.4 µl of diluted cDNA, 800 nM forward and reverse primers and 1x LightCyclerR 480 SYBR Green I Master were carried out in a 96-well LightCycler 480 Multiwell Plate. Polymerase chain reactions were carried out in a LightCycler 480 using the program detailed above. Primers used are listed in below. Expression data was analysed using LightCycler 480 Software v1.5.0.39. Primers for real-time PCR were designed using the IDT RealTime PCR web tool with default parameters except for amplicon size, which was set to 100 bp minimum, 150 bp optimum and 200 bp maximum.

**List of qPCR Primers.**

| Gene | Species | Forward primer (5'-3') | Reverse Primer (5'-3') |
|---|---|---|---|
| **Ptn** | Mouse | AATGTGACCTCAATACCGCC | TCTCCTGTTTCTTGCCTTCC |
| **Vcam1** | Mouse | AGCAAAGACAGGAGACATGG | CAGTAGAGTGCAAGGAGTTCG |
| **Bdnf** | Mouse | ACCAGGTGAGAAGAGTGATG | AGTGTCAGCCAGTGATGTC |
| **Cxcl1** | Mouse | ACCCAAACCGAAGTCATAGC | GGACCCTCAAAAGAAATTGTATAGTG |
| **Msc** | Mouse | CTACGAGGACAGCTATGTGC | GAGAAGGTCCAGAATCCAGTG |
| **Nid1** | Mouse | CTCCAGTATCCTTTCGCTGTG | AGCAGTAATTGTGGCCTTGG |
| **Efna5** | Mouse | TTGGCAATCCTACTGTTCCTC | GTTAGGTGGATCTCTGGTGTTC |
| **Ddr2** | Mouse | CTGTCGGATGAGCAGGTTATC | CAGCTTATACACAGAGTCGGG |
| **TMEM194** | Mouse | TGACCCCAAACTCTTCCTTG | CCTACCAGGATGACGTAAATGG |

## Fluorescence In Situ Hybridization (FISH)

For FISH experiments C2C12 MBs and MTs were cultured on coverslips which were washed in PBS prior to fixation in 4% para-formaldehyde, 1X PBS for 10 min at room temperature. Satellite cells and primary myofibers were cultured on matrigel cultured coverslips and fixed identically. After aging coverslips for several days, cells were permeabilized for 6 min with 0.2% Triton-X-100 in PBS, followed by 3 washes in PBS. If antibody staining was required coverslips were blocked with 2% BSA prior to sequential incubations with primary and secondary antibodies. After washing, antibodies were fixed for 45 s in 2% paraformaldehyde, PBS. Cells were next pre-equilibrated in 2X SCC and treated with RNase A (100µg/ml) at 37ºC for 1 h. Following washing in 2X SCC, cells were dehydrated with a 70%, 85% and 100% ethanol series. Coverslips were then air dried, heated to 70ºC and submerged into 85ºC preheated 70% formamide, 2X SSC (pH 7.0) for 21 min. A second ethanol dehydration series was then performed using -20ºC 70% ethanol for the first step. Coverslips were air dried and 150-300 ng biotin-/digoxigenin-/fluorophore-labelled probe was added in hybridization buffer (50% formamide, 2X SSC, 1% Tween20, 10% Dextran Sulphate) containing 6 µg human Cot1 DNA (Invitrogen) and sheared salmon sperm DNA and incubated at 37°C for 24 h in a humidified chamber. For gene specific FISH, probes were generated from BACs by end labelling while chromosome paints were purchased pre-labelled. After incubation, the coverslips were washed four times for 5 min each in 4X SSC at 50ºC followed by four times for 5 min each in 0.1X SSC at 65ºC. Coverslips were then pre-equilibrated in 4X SSC, 0.1% Tween-20 and blocked with 4% BSA before incubating for 1 h at room temperature with Alexa Fluor®conjugated-Steptavidin/anti-dioxigenin antibodies and 4,6-diamidino-2 phenylindole, dihydrochloride (DAPI) at 2 µg/ml. Coverslips were subsequently washed 3 times in 4X SSC, 0.1% Tween-20 at 37ºC and mounted on slides in Vectashield (Vector Labs). Mouse chromosome 8 and human chromosome 5 were purchased from Metasystems and Cambridge Bioscience, respectively. BACs were ordered as indicated below.

**List of FISH probes.**

| Locus | Chr | BAC | Source |
|---|---|---|---|
| ***Ttn*** | 2 | BMQ379B2 | Source Biosystems |
| ***Efna5*** | 17 | BMQ-206B15 | Source Biosystems |
| ***Nid1*** | 13 | BMQ-452C6 | Source Biosystems |
| ***Ptn*** | 6 | BMQ-358G8 | Source Biosystems |
| ***Msc*** | 1 | CH29-615N9 | Source Biosystems |
| ***DDR2*** | 1 | BMQ-350O7 | Source Biosystems |
| ***Cxcl1*** | 5 | BMQ-214F8 | Source Biosystems |
| ***Bdnf*** | 2 | BMQ-60G18 | Source Biosystems |
| ***Vcam1*** | 3 | CH29-72H24 | BACPAC |
| ***Chromosome 8*** | 8 | N/A | Metasystems (000000-0528-839) |

## Determination of FISH-labelled Gene and Chromosome Position

The position of the loci and chromosomes was determined using previously designed macros written in Visual Basic and run on Image Pro Plus (available on request). Briefly, the macro calculates the DAPI-identified nuclear cross-sectional area and then sequentially erodes 20% of this in 4 stages to generate 5 shells of roughly equal area. The shell containing the locus was then determined and the number of events in each shell summed. The percentage of total events in each shell was then calculated in excel. For chromosomes the fraction of chromosome in each bin was calculated. Due to the larger size of chromosomes bins 1 and 2 were termed peripheral and bins 4 and 5 were termed internal. Only cells which had the locus/chromosome in focus at the mid-plane of the nucleus were analyzed.

## Tissue Sections and Immunofluorescence Staining

The tissue samples of gastrocnemius muscle were provided by Dr. Benedikt Schoser (Friedrich-Baur Institute, Dept. of Neurology at the Ludwig-Maximilians-University of Munich, Germany). Liver and Brain sections were isolated from male J6 mice sacrificed by cervical dislocation. Tissue blocks were snap frozen using liquid nitrogen in OCT mounting medium and mounted on cryostat chucks using OCT mounting medium (Tissue-Tek). Sections were cut to a thickness of 10 µm using a cryostat (Leica CM1900) and mounted on SuperFrost Plus (VWR) slides. Slides were immediately placed on dry ice and stored at -80$^{o}$C. Primary myotubes stained were grown in tissue culture on matrigel-coated coverslips. For staining, sections were first equilibrated at RT and OCT mounting medium (Tissue-Tek) removed. Sections were incubated in blocking solution (1% Fish gelatine (G-7765, Sigma) in TBS + 0.1% Tween) for 30 min followed by primary antibodies overnight at 4$^{o}$C. Sections were washed 3 x 5 min using TBS + 0.1% Tween and incubated with secondary antibodies and DAPI for 1 h and washed as previous. Sections were then mounted in Vectashield (Vector Laboratories).

## 3view Electron Microscopy

MBs were seeded and differentiated to MTsin glass-bottomed dishes (MatTek) for 6 days. MTs were then fixed (2% Paraformaldehyde, 2% Glutaraldehyde) and stained with heavy metals: reduced osmium tetroxide, tannic acid, osmium tetroxide, uranyl acetate and lead aspartate. Samples were dehydrated in a graded series of ethanol before embedding in Agar 100 resin. Following polymerisation at 60°C for 48 h, hardened resin samples were trimmed using an ultra-microtome and mounted onto a pinhead. Serial block-face EM was performed using a Gatan 3View. Full reconstruction of samples was achieved using 600 x 50 nm sections per sample.

## Determination of MT Fusion Frequency

Empty vector- and NET shRNA-treated MBs were differentiated on coverslips for 6 days and subsequently fixed in 4% formaldehyde, PBS. All nuclei were then stained with DAPI while MTs were selectively stained using an anti-myosin heavy chain 1 (Myh1) antibody as described in the above sections. 5-10 fields across 3 biological replicates were then imaged and the number of nuclei with significant signal overlap with Myh1 staining were counted and determined to reside within MTs and those without were determined to represent MBs. The fraction of total nuclei present within MTs was then determined.

## Microscopy

Images were acquired on a Nikon TE-200 microscope using a 1.45 NA 100x objective, Sedat quad filter set, PIFOC Z-axis focus drive (Physik Instrubments) and a CoolSnapHQ High Speed Monochrome CCD camera (Photometrics) run by Metamorph image acquisition software.

## IncuCyte™ Visualization of MT Formation

Empty vector- and NET shRNA-treated MBs were plated in 6 well plates at confluency and induced to differentiate after 48 h by addition of differentiation medium in an IncuCyte ZOOM® system. MTs were then allowed to form over the next 4 days with media changed every 48 h. Cells were imaged every 30 min over 16 fields per well using the IncuCyte™ 10x objective. For Supplemental Movie 1 a single field per condition was selected and the collected images assembled into a movie using the IncuCyte ZOOM® system software.

## Western Blotting

Protein samples were prepared from cells after directly lysing in TRIzol™ following the manufacturer's protocol. Protein concentration was quantified following resuspension in 20 mM Tris, pH 8.0, 1% SDS using the Pierce® BCA Protein Assay Kit. Cell lysates were separated on 8-12% Tris-glycine-SDS or Bis-Tris gels. Subsequently the gels were transferred onto nitrocellulose membranes (Odyssey 926-31092) by means of semi-dry transfer (BIO-RAD). After transfer the membrane was blocked in western blot blocking buffer (5% milk powder in PBS with 0.05% Tween-20) for 30 min. Subsequently, the membrane was incubated with the primary antibody diluted in Western blot blocking buffer at the dilutions indicated in the Antibodies section above for 60 min at room temperature or overnight at 4°C. Six washes in PBS, 0.05% Tween-20 were then followed by incubation with the secondary antibody conjugated to an IRDye® for 60 min at room temperature. After 6 washes in PBS, 0.05% Tween20, membranes IRdye®-conjugated antibodies were detected on a Li-Cor Odyssey Quantitative Fluorescence Imager.

# SUPPLEMENTAL REFERENCES


Asp, P., Blum, R., Vethantham, V., Parisi, F., Micsinai, M., Cheng, J., Bowman, C., Kluger, Y., and Dynlacht, B.D. (2011). Genome-wide remodeling of the epigenetic landscape during myogenic differentiation. Proceedings of the National Academy of Sciences of the United States of America *108*, E149-158.

Benjamini, Y., and Hochberg, Y. (1995). Controlling the false discovery rate: a practical and powerful apporach to multiple testing. Journal Royal Statistical Society Series B *57*, 289-300.

Falcon, S., and Gentleman, R. (2007). Using GOstats to test gene lists for GO term association. Bioinformatics *23*, 257-258.

Greber, U.F., Senior, A., and Gerace, L. (1990). A major glycoprotein of the nuclear pore complex is a membrane-spanning polypeptide with a large lumenal domain and a small cytoplasmic tail. Embo Journal 9, 1495-1502.

Li, H., and Durbin, R. (2009). Fast and accurate short read alignment with Burrows-Wheeler transform. Bioinformatics *25*, 1754-1760.

Quinlan, A.R., and Hall, I.M. (2010). BEDTools: a flexible suite of utilities for comparing genomic features. Bioinformatics *26*, 841-842.

R Development Core Team (2010). R: A language and environment for statistical computing. (Vienna, Austria, R Foundation for Statistical Computing).

Ritchie, M.E., Phipson, B., Wu, D., Hu, Y., Law, C.W., Shi, W., and Smyth, G.K. (2015). limma powers differential expression analyses for RNA-sequencing and microarray studies. Nucleic Acids Reearch *43*, e47.

Salmon, P., and Trono, D. (2007). Production and titration of lentiviral vectors. Current protocols in human genetics / editorial board, Jonathan L Haines  [et al] *Chapter 12*, Unit 12 10.

Schirmer, E.C., Florens, L., Guan, T., Yates, J.R.r., and Gerace, L. (2003). Nuclear membrane proteins with potential disease links found by subtractive proteomics. Science *301*, 1380-1382.

Schirmer, E.C., Guan, T., and Gerace, L. (2001). Involvement of the lamin rod domain in heterotypic lamin interactions important for nuclear organization. The Journal of Cell Biology *153*, 479-489.

Smyth, G.K. (2005). Limma: linear models for microarray data. In Bioinformatics and Computational Biology Solutions Using R and Bioconductor, R. Gentleman, V. Carey, S. Dudoit, R. Irizarry, and W. Huber, eds. (New York, Springer), pp. 397-420.

Soullam, B., and Worman, H.J. (1993). The amino-terminal domain of the lamin B receptor is a nuclear envelope targeting signal. The Journal of Cell Biology *120*, 1093-1100.

Takagi, M., Absalon, M.J., McLure, K.G., and Kastan, M.B. (2005). Regulation of p53 translation and induction after DNA damage by ribosomal protein L26 and nucleolin. Cell *123*, 49-63.

Uhlen, M., Fagerberg, L., Hallstrom, B.M., Lindskog, C., Oksvold, P., Mardinoglu, A., Sivertsson, A., Kampf, C., Sjostedt, E., Asplund, A.*, et al.* (2015). Proteomics. Tissue-based map of the human proteome. Science *347*, 1260419.

Vogel, M.J., Peric-Hupkes, D., and van Steensel, B. (2007). Detection of in vivo protein-DNA interactions using DamID in mammalian cells. Nature Protocols *2*, 1467-1478.

Wilkie, G.S., Korfali, N., Swanson, S.K., Malik, P., Srsen, V., Batrakou, D.G., de las Heras, J., Zuleger, N., Kerr, A.R., Florens, L.*, et al.* (2011). Several novel nuclear envelope transmembrane proteins identified in skeletal muscle have cytoskeletal associations. Molecular & cellular proteomics : Molecular Cellular Proteomics *10*, M110 003129.

Wu, F., and Yao, J. (2013). Spatial compartmentalization at the nuclear periphery characterized by genome-wide mapping. BMC genomics *14*, 591.

Yue, F., Cheng, Y., Breschi, A., Vierstra, J., Wu, W., Ryba, T., Sandstrom, R., Ma, Z., Davis, C., Pope, B.D., *et al.* (2014). A comparative encyclopedia of DNA elements in the mouse genome. Nature *515*, 355-364.

Zang, C., Schones, D.E., Zeng, C., Cui, K., Zhao, K., and Peng, W. (2009). A clustering approach for identification of enriched domains from histone modification ChIP-Seq data. Bioinformatics *25*, 1952-1958.

Zuleger, N., Kelly, D.A., Richardson, A.C., Kerr, A.R., Goldberg, M.W., Goryachev, A.B., and Schirmer, E.C. (2011). System analysis shows distinct mechanisms and common principles of nuclear envelope protein dynamics. The Journal of Cell Biology *193*, 109-123.
